# Repurposing Itraconazole and Hydroxychloroquine to Target Lysosomal Homeostasis in Epithelial Ovarian Cancer

**DOI:** 10.1158/2767-9764.CRC-22-0037

**Published:** 2022-05-04

**Authors:** Stefano Marastoni, Ainhoa Madariaga, Aleksandra Pesic, Sree Narayanan Nair, Zhu Juan Li, Zvi Shalev, Troy Ketela, Ilaria Colombo, Victoria Mandilaras, Michael Cabanero, Jeff P. Bruce, Xuan Li, Swati Garg, Lisa Wang, Eric X. Chen, Sarbjot Gill, Neesha C. Dhani, Wenjiang Zhang, Melania Pintilie, Valerie Bowering, Marianne Koritzinsky, Robert Rottapel, Bradly G. Wouters, Amit M. Oza, Anthony M. Joshua, Stephanie Lheureux

**Affiliations:** 1 Princess Margaret Cancer Centre, University Health Network, Toronto, Ontario, Canada.; 2 Division of Medical Oncology & Hematology, Princess Margaret Cancer Centre, Toronto, Ontario, Canada.; 3 Autonomous University of Barcelona, Barcelona, Spain.; 4 Department of Pathology, Toronto General Hospital, Toronto, Ontario, Canada.; 5 Department of Biostatistics, Princess Margaret Cancer Centre, University Health Network, Toronto, Ontario, Canada.; 6 Institute of Medical Science, University of Toronto, Toronto, Ontario, Canada.; 7 Department of Medical Biophysics, University of Toronto, Toronto, Ontario, Canada.; 8 Department of Radiation Oncology, University of Toronto, Toronto, Ontario, Canada.; 9 Kinghorn Cancer Centre, Department of Medical Oncology, St Vincents Hospital, Sydney, Australia.; 10 Garvan Institute of Medical Research, Sydney, Australia.

## Abstract

Drug repurposing is an attractive option for oncology drug development. Itraconazole is an antifungal ergosterol synthesis inhibitor that has pleiotropic actions including cholesterol antagonism, inhibition of Hedgehog and mTOR pathways. We tested a panel of 28 epithelial ovarian cancer (EOC) cell lines with itraconazole to define its spectrum of activity. To identify synthetic lethality in combination with itraconazole, a whole-genome drop-out genome-scale clustered regularly interspaced short palindromic repeats sensitivity screen in two cell lines (TOV1946 and OVCAR5) was performed. On this basis, we conducted a phase I dose-escalation study assessing the combination of itraconazole and hydroxychloroquine in patients with platinum refractory EOC (NCT03081702). We identified a wide spectrum of sensitivity to itraconazole across the EOC cell lines. Pathway analysis showed significant involvement of lysosomal compartments, the trans-golgi network and late endosomes/lysosomes; similar pathways are phenocopied by the autophagy inhibitor, chloroquine. We then demonstrated that the combination of itraconazole and chloroquine displayed Bliss defined synergy in EOC cancer cell lines. Furthermore, there was an association of cytotoxic synergy with the ability to induce functional lysosome dysfunction, by chloroquine. Within the clinical trial, 11 patients received at least one cycle of itraconazole and hydroxychloroquine. Treatment was safe and feasible with the recommended phase II dose of 300 and 600 mg twice daily, respectively. No objective responses were detected. Pharmacodynamic measurements on serial biopsies demonstrated limited pharmacodynamic impact. *In vitro*, itraconazole and chloroquine have synergistic activity and exert a potent antitumor effect by affecting lysosomal function. The drug combination had no clinical antitumor activity in dose escalation.

**Significance:**

The combination of the antifungal drug itraconazole with antimalarial drug hydroxychloroquine leads to a cytotoxic lysosomal dysfunction, supporting the rational for further research on lysosomal targeting in ovarian cancer.

## Introduction

Epithelial ovarian cancer (EOC) is the most lethal gynecologic malignancy in developed countries (1). It consists of five subtypes—high- and low-grade serous, endometrioid, clear cell, and mucinous carcinomas (2). Treatment for advanced EOC is multimodal; integrating surgery, platinum-based chemotherapy and maintenance therapy, which are tailored according to histologic subtype, stage, and patient characteristics. Contemporary systemic treatments in recurrent EOC include chemotherapy, antiangiogenic agents and PARP inhibitors (3). Ultimately, platinum-resistant EOC—defined as disease progression occurring in <6 months from last dose of platinum—remains an aggressive disease with limited effective treatment options, and a response to chemotherapy of approximately 10%–15% (3, 4).

An emerging strategy to expand the therapeutic armamentarium against ovarian cancer is drug repurposing. A widely used antifungal drug, itraconazole has been investigated in several cancer types (5–7). The current understanding of the mechanisms of action of itraconazole include: (i) potent antiangiogenic and antilymphangiogenic activity (8, 9), (ii) modulating Hedgehog and Wnt/β-catenin signaling (10, 11), (iii) inhibiting the mitochondrial protein voltage-dependent anion channel 1 (VDAC1) (12), and (iv) altering cholesterol trafficking by direct binding and inhibition of the lysosomal protein and cholesterol transporter Niemann–Pick disease, type C1 (NPC1; ref. 13). These latter two effects have been demonstrated to result in inhibition of the mTOR pathway (14). A number of preclinical and clinical studies have suggested that patients with prostate, lung, and basal cell carcinoma may benefit from treatment with itraconazole monotherapy or in combination with chemotherapy (5–7, 15–17). In platinum-resistant EOC, two retrospective studies in patients with recurrent ovarian cancer have suggested the beneficial effects of itraconazole in combination with taxane-based chemotherapy (18, 19). We were stimulated to understand which pathways can be targeted to increase itraconazole efficacy and pursue more potent combinations.

Recently, genome-scale clustered regularly interspaced short palindromic repeats (CRISPR)-Cas9–based screens have proven to be a valuable and robust approach to uncovering novel understanding of cell biology and drug discovery. Chemogenetic profiling using CRISPR-Cas9 screens performed in combination with small-molecule inhibitors allows the identification of gene abnormalities that enhance or suppress the activity of chemical compounds. This knowledge provides understandings into drug mechanism of action, genetic vulnerabilities, and resistance mechanisms, all of which help stratify patients and improve drug efficacy (20). Using this approach, we defined genomic targets associated with lysosomal function and dynamics as sensitizing ovarian cancer cells to nontoxic concentrations of itraconazole.

Lysosomes are membrane-enclosed intracellular organelles that are fundamental for cellular homeostasis, specifically the degradation of proteins that have been internalized by cells through endocytosis and phagocytosis. During endosomal maturation to lysosomes, hydrolases are produced in the endoplasmic reticulum (ER) and transported to the Golgi apparatus, where they receive the lysosomal-targeted mannose-6-phosphate (M6P) tag (21, 22). In the maturation process, the compartmental pH drops, and more than 50 acid hydrolases are activated, maintained by the action of vacuolar H^+^-ATPase, to degrade molecules delivered via endocytic, phagocytic, and autophagic pathways (22, 23). Lysosome homeostasis is an attractive target for cancer therapy as lysosomes have been reported to be associated with chemoresistance, survival under physiologic stress, increased invasion and metastasis and cancer progression (22–24). Interestingly, high expression of the lysosome-associated membrane protein-1 (LAMP1) has been reported as a poor prognostic marker in patients with EOC (25).

Among several drugs that target lysosomes, only hydroxychloroquine is being currently examined in clinical trials for cancer (23). Chloroquine, and its derivative hydroxychloroquine, accumulate in acidic compartments of the cells, increasing the pH leading to hydrolase inhibition and lysosomal dysfunction (22, 23, 26, 27). We found that nontoxic concentrations of chloroquine increase the cytotoxic effects of itraconazole in EOC. We thereafter explored the combination of itraconazole and hydroxychloroquine in a phase I clinical trial in platinum-resistant EOC (NCT03081702).

## Materials and Methods

### Cell Lines and Cell Culture

Ovarian cancer cells were kindly provided by Dr. Rottapel in 2015 (originally obtained by Ben Neel, Graham Fletcher, Anne-Marie Mes-Massons, Patricia Tonin, Gordon Millsand James Brenton) and were cultured in either OSE (Wisent), RPMI1640 supplemented with 10 mmol/L HEPES (Life Technologies), DMEM (Life Technologies), or DMEM/F12 (Wisent), with 10% FBS (Gibco), depending on the cell type, as reported previously (28). More detailed information is provided in the Supplementary Materials and Methods. HEK293T cells were obtained in 2013 from ATCC (CRL-3216, RRID:CVCL_0063). All cell lines were cultured at 37°C in a 5% CO_2_ humidified incubator and were cultured for a maximum of 15 passages. Regular cell-line authentication was done at The Centre for Applied Genomics (TCAG, http://www.tcag.ca) using the GenePrint10 System (Promega Corporation) according to the manufacturer's instructions. All cell lines were routinely tested to confirm the absence of *Mycoplasma* using the Mycoalert Detection Kit (Lonza).

### Antibodies, Drugs, and Reagents

Anti-Cas9 (sc-517386, RRID:AB_2800509) antibody was purchased from Santa Cruz Biotechnology. Anti-beta Tubulin (ab6046, RRID:AB_2210370) antibody, anti-p62 (ab56416, RRID:AB_945626) were purchased from Abcam. Anti-eIF4E (#610270, RRID:AB_397665) was purchased from BD Biosciences. Anti-LAMP1 (#9091, RRID:AB_2687579), anti-Rab7 (#9367, RRID:AB_1904103) and anti-CC3 (#9661, RRID:AB_2341188) were purchased from Cell Signaling Technology. Anti-*VPS54* antibody (#13327-1-AP, RRID:AB_2304414) was purchased from Proteintech. Anti-Ki-67 (clone: MIB1, M7240, RRID:AB_2142367) was purchased from Agilent Dako.

Secondary antibodies used for Western blotting were purchased from Licor. Secondary antibodies used from immunofluorescence, Hoechst (NucBlue Live Ready Probes) and DAPI were purchased from Thermo Fisher Scientific.

Itraconazole and chloroquine were purchased from Sigma-Aldrich. Alamar blue cell viability reagent was purchased from Thermo Fisher Scientific. Incucyte Caspase-3/7 Green Dye for Apoptosis was purchases from Sartorius (Essen Bioscience, #4440). Puromycin and blasticidin solutions were purchased from Invivogen.

Filipin III from Streptomyces filipinensis reagent (F4767-5MG) was purchased from Sigma-Aldrich. Lysosomal Intracellular Activity Assay Kit was purchased from Biovision. Polybrene Infection/Transfection reagent was purchased from Milllipore. The QIAamp Blood Maxi Kit used for genomic isolation of CRISPR screen samples was purchased from QIAGEN.

Genomic DNA (gDNA) isolation kit was purchased from Norgen Biotech Corp. High-fidelity master mix used for PCR was purchased from New England Biolabs. All-Prep DNA/RNA/miRNA universal Co-Isolation kit was purchased from QIAGEN. UltraView Detection Kit was purchased from Vantana.

### CRISPR Screen

To produce the whole-genome single-guide RNA (sgRNA) library with TKOV1 lentivirus (29), 293T cells were transfected with psPAX2 (lentiviral packaging; Addgene #12260), pMD2.G (VSV-G envelope; Addgene #12259), and TKOV1 (Toronto KnockOut CRISPR Library, version 1; Addgene #1000000069) as reported previously (20).

To transduce the OVCAR5 and TOV1946 cells, the TKOV1 virus was added with 8 μg/mL polybrene in 15 cm dishes. Cells were selected with puromycin at 48 hours postinfection. After selection cells were grown for 5 days to stabilize and then divided in triplicate. Itraconazole was added to separate replicates at a final concentration of 1 μmol/L, with one set of replicates receiving no drug treatment. Subconfluence cell cultures were trypsinized, counted, and replated, and the excess cell pellets were frozen at − 20 °C as a timepoint. Once at least eight doublings were reached from T0, the screens were terminated, and pellets frozen at − 20 °C. Coverage of screens was kept at 400 cells per gRNA.

gDNA was isolated from the frozen cell pellets using the QIAamp Blood Maxi Kit (Qiagen), submitted for PCR and next-generation sequencing and analyzed as reported previously (20).

### Alamar Blue Assay

Cell viability was determined using the Alamar blue assay according to the manufacturer's protocol. Briefly, cells were plated in 96-well plates (2–4 × 10^3^ cells/100 μL/well) and let attach overnight. Then they were treated with serial dilutions of itraconazole at a final concentration of 0–40 μmol/L with or without chloroquine 5–10 μmol/L. Cells were tested after 5 days by adding Alamar blue solution to each well, incubating for 6 hours and measuring absorbance with a microplate reader (FLUOstar Omega, BMG Labtech) at a test wavelength of 550 nm. All the experiments were performed independently three times.

### DNA Sequencing of *C18orf8* (*RMC1*)

gDNA from CRISPR and control cells was isolated from 1 million cells pellets using the gDNA Isolation Kit from Norgen Biotech Corp. DNA purity and concentration was determined using Nanodrop (Thermo Fisher Scientific). Then, a region in Exon2 (targeted by *C18orf8* sgRNA) was amplified using the Q5 High-Fidelity 2X Master Mix (New England Biolabs) and the following primers: forward primer CTTGCTGCTTTTCCCTCTCA, reverse primer ACCTAAATGAGATGGGATTCCT. *C18orf8*-specific PCR band was isolated from agarose gel using the QIAquick Gel Extraction kit (QIAGEN) and eluted in water. Samples were submitted for sequencing using the above primers to the ACGT Corp (http://acgtcorp.com/).

### Western Blotting

Proteins were extracted from cell lines by RIPA buffer (NORGEN Biotek Corp) with protease and phosphatase inhibitors (Roche). After centrifugation at 10,000 × *g* supernatants were boiled in Laemmli buffer for 10 minutes and proteins were resolved by SDS-PAGE in Bolt 4%–12% Bis-Tris Plus Gels (Thermo Fisher Scientific). Proteins were subsequently transferred onto polyvinylidene difluoride membrane (Thermo Fisher scientific) and blocked for 1 hour with Odyssey Blocking Buffer in TBS (LI-COR), incubated overnight with primary antibodies Anti-Cas9 (sc-517386), Anti-beta Tubulin (ab6046), anti-eIF4E (#610270) anti-*VPS54* antibody (#13327-1-AP), and for 1 hour at room temperature in IRDye 680RD and IRDye 800CW conjugated IgG (LI-COR). Western blots were visualized using the Odyssey Infrared Imaging System (LI-COR Biosciences). Anti-beta Tubulin (ab6046) and anti-eIF4E (#610270) were used as loading control.

### Lysosomal Assay and Immunofluorescence

Lysosomal function was tested using the Lysosomal Intracellular Activity Assay Kit (Biovision) according to the manufacturer's instructions. LAMP1 immunofluorescence was used to check differences in lysosomal pattern among the different conditions. Detailed information is included in the Supplementary Materials and Methods.

### Bioinformatic Analysis

Itraconazole activity area was calculated using GraphPad Prism v6. Cell lines with normalized activity area at least 0.8 SDs above the mean were defined as sensitive to the compound, whereas those with activity area at least 0.8 SDs below the mean were defined as resistant. Cell lines with activity area within 0.8 SDs of the mean were considered to be intermediate (30, 31). The drugZ algorithm was used to identify chemogenetic interactions from CRISPR screen as reported previously (20). Venny software (https://bioinfogp.cnb.csic.es/tools/venny/) was used to generate Venn diagrams. Pathway analysis on significant CRISPR screen hits (FDR and *P* < 0.05) was performed using pathDIP (http://ophid.utoronto.ca/pathDIP/; ref. 32). Itraconazole inhibitory percentage activity and heatmaps were generated using SynergyFinder 2.0 (https://synergyfinder.fimm.fi/) and Excel. Itraconazole/chloroquine synergy score was calculated on the basis of Bliss reference model. Calculation of synergy scores were obtained using SynergyFinder 2.0 as reported previously (33).

### Study Design HYDRA-1 (ClinicalTrials.gov, NCT03081702)

A rolling-six phase I design was used to assess the combination of itraconazole (PrMint-itraconazole, itraconazole capsules, Mint Pharmaceuticals Inc.) and hydroxychloroquine, (PrMint-hydroxychloroquine, hydroxychloroquine sulfate capsules, Mint Pharmaceuticals Inc.), in patients with platinum-resistant or refractory EOC. The study was conducted in accordance with ethical guidelines and approved by the Institutional Review Board. A written informed consent was obtained from all patients. Women received itraconazole 300 mg twice daily (twice daily) with hydroxychloroquine as per dose-escalation schedule (200 mg twice daily in dose level (DL)1; 400 mg twice daily in DL2; 600 mg twice daily in DL3), continuously in a 28-day cycle. The fixed dose of itraconazole was determined in previous phase II trials (6), and because there is no known interaction based on metabolism and pharmacokinetics or overlapping toxicities, a rapid dose escalation of hydroxychloroquine, through a rolling-six design was incorporated (34–36). Dose-limiting toxicities (DLT) were defined as grade ≥4 anemia, neutropenia or thrombocytopenia, febrile neutropenia, grade ≥3 diarrhea or rash and grade ≥2 ocular toxicity, other grade ≥3 related toxicity or grade 2 adverse events that do not recover within 2 weeks after optimal treatment during the initial 28 days of treatment (protocol in Supplementary Materials and Methods). The calculated sample size was between 6 and 18.

Tumor assessment occurred every 8 weeks (± 1 week) by CT scan. Toxicity was assessed by Common Terminology Criteria version 4.0. Pretreatment and on-treatment biopsies (on cycle 1, days 8 to 14) were mandatory.

The primary objective was establishment of MTD; secondary objective was objective response rate by RECIST v1.1, and progression-free survival (PFS).

### Patients

Enrolled patients were ≥ 18 years old with an Eastern Cooperative Oncology Group performance status of 0–1, and platinum resistant or refractory EOC. Eligible patients had adequate blood and marrow function (hemoglobin ≥90 g/L, absolute neutrophils ≥1.5 × 10^9^/L, platelets ≥100 × 10^9^/L, bilirubin within normal limits, aspartate aminotransferase ≤2.5 × institutional upper limit of normal, alanine transaminase, serum creatinine within normal limits or creatinine clearance ≥60 mL/minute). Women on cytochrome P450 3A4 (CYP3A4) inhibitors/inducers or on statins were ineligible. Patients with a known glucose-6-phosphate dehydrogenase deficiency or a known retinopathy were ineligible. Patients with a clinical indication for treatment with itraconazole or hydroxychloroquine, and those with intestinal malabsorption or active bowel obstruction were excluded. There was no limitation regarding prior number of lines of therapy.

### Clinical Trial Exploratory Objective Methods

IHC was performed using the BenchMark XT automated stainer (Ventana Medical System) with antigen retrieval (CC1, Tris/Borate/EDTA pH8.0, #950-124) for 64 minutes. The Ki-67 (clone: MIB1, M7240, Dako) dilution was 1:100 with 60-minute incubation. The anti-CC3 (D175, #9661, Cell Signaling Technology) dilution was 1:500 with 32-minute incubation. The anti-LAMP1 (#9091, Cell Signaling Technology) dilution was 1:1,000 with 60-minute incubation. The anti-P62 (2C11, WH000887M1, Sigma) dilution was 1:4,000 with 32-minute incubation. The Ventana'’s ultraView Detection Kit (#760-500) was utilized and the slides were counterstained with Gill-modified hematoxylin.

The H-score method was used to assess immunoreactivity for cleaved caspase 3 (CC3), p62, and LAMP1. In brief, the H-score is obtained by the formula: 3 × percentage of strong staining + 2 × percentage of moderate staining + percentage of weak staining, resulting in a range from 0 to 300. The Ki-67 proliferation index was visually estimated to the nearest 5% increment.

IHC slides were scanned at a 20× magnification using the Aperio Scanscope AT2 Whole Slide Scanner (Leica) and IHC images were obtained using Aperio ImageScope software (Leica).

### Tissue Pharmacokinetic Analyses

Tumor itraconazole and hydroxychloroquine concentrations were determined with itraconazole-D9 and hydroxychloroquine-D4 as the internal standard. The high-performance liquid chromatography (HPLC) system was interfaced to a SCIEX TRIPLE QUAD 6500+ mass spectrometer operating in the negative electrospray ionization mode. Data collection, peak integration, and calculation were performed using Analyst 1.7 software (Sciex).

### Statistical Analysis

All dose–response graphs were generated using GraphPad Prism v6. Statistical analysis on the CRISPR screen results was done as reported previously (20). Hits with a *P* value and FDR <0.05 were selected for pathway analysis. Pathway analysis on CRISPR screen significant hits using PathDIP software uses Fisher exact test and corrects raw *P* values for multiple hypothesis testing based on Bonferroni and FDR (BH method) methods (32). Statistical significance of differences among groups (apoptosis, synergy scores in *VPS54* and *C18orf8* knockout cells and median normalized groups in lysosomal function and size) was assessed using the one-way ANOVA. To utilize all individual measurements of lysosomal diameter and function, the linear mixed effect modeling was applied with lysosomal function and size (diameter) as dependent variable and treatment, the type of the cells (resistant or sensitive) and their interaction as explanatory variable. The repeats and cell type (COV318, OVCAR5, TOV21G, TOV1946, TOV3133G) were random variables with repeats nested within the cell type. To stabilize the variance, a log transformation was applied to both lysosomal function and size measurements. Histograms and Swimmer plots were generated using R software. Patient clinical features and response details were described using summary statistics, such as medians, ranges, frequencies, and proportions. PFS analysis was conducted using the Kaplan–Meier method for all patients. Median and confidence interval were reported to assess PFS. Treatment-related toxicity was evaluated using frequencies and proportions of adverse events based on severities and attributions.

### Data Availability Statement

The data generated in this study are available upon request from the corresponding author.

## Results

### Evaluation of Therapeutic Potential of Itraconazole in Ovarian Cancer

**FIGURE 1:**
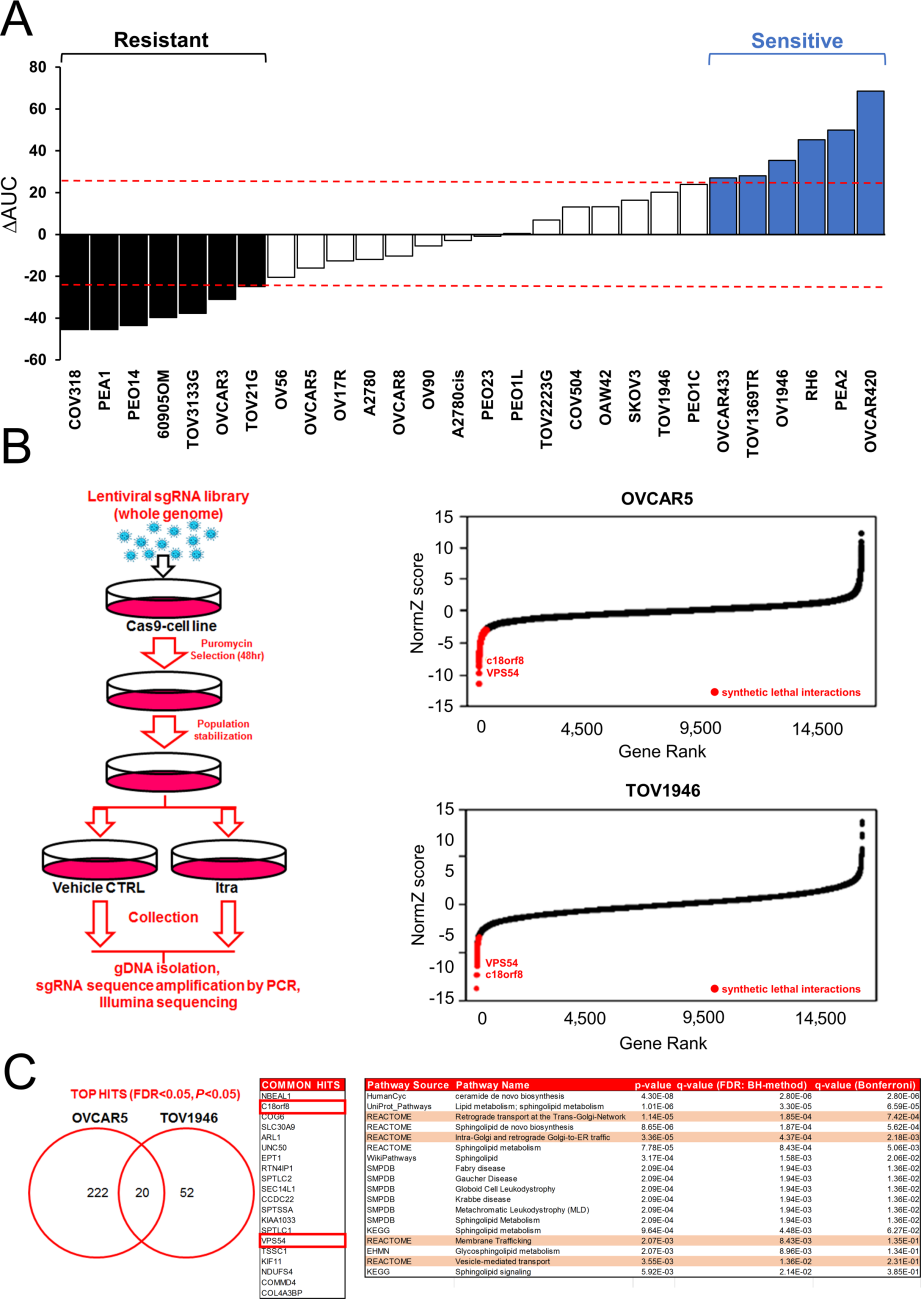
Vesicular trafficking is an important regulator of itraconazole resistance. **A,** Activity area analysis showing the activity of itraconazole in panel of 28 ovarian cancer cell lines. Cells were treated for 5 days with 0–40 μmol/L itraconazole (*n* = 3 biological replicates; see Supplementary Fig. S1). **B,** Left, schematic representation of itraconazole chemogenetic CRISPR screen. Right, graphs showing DrugZ-calculated normZ score in OVCAR5 and TOV1946 cells. Synergistic/synthetic lethal interactions are reported in red at FDR and *P* value < 0.05. **C,** Left-middle, Venn diagram showing overlap between the top hits from OVCAR5 and TOV1946 CRISPR screens and table showing common hits. Right, bioinformatic analysis showing pathways involved in the regulation of itraconazole sensitivity using PathDIP annotated database.

To explore the therapeutic potential of itraconazole in the treatment of EOC, we screened a panel of 28 cells lines with 5 days of exposure. Quantitative scoring of differential itraconazole sensitivity was calculated using the activity area method (corresponding to the area over the drug–response curve) as reported previously (refs. 30, 31; Fig. 1A; Supplementary Fig. S1), and a threshold of 0.8 SD on the mean value was employed to identify the spectrum of sensitive and resistant cell lines, resulting in seven resistant, 15 intermediate, and 6 sensitive cell lines (31). These data suggest that itraconazole has a cytotoxic effect in a subgroup of ovarian cancer cells.

### Lysosomal Compartments as Important Regulators of Itraconazole Resistance Identified through a CRISPR Screen

To identify genes and pathways involved in sensitizing cells to itraconazole, we performed a whole genome drop-out CRISPR screen in two cell lines of intermediate sensitivity (TOV1946 and OVCAR5). We stably expressed Cas9 (Fig. 1B; Supplementary Fig. S2A) and verified its activity (Supplementary Fig. S2A and S2B). Infected cells were treated with nontoxic concentrations of itraconazole (1 μmol/L; Fig. 1B; Supplementary Fig. S2C). Analysis of the synthetically lethal hits using DrugZ algorithm (20) with both a FDR and *P* value <0.05, reported 72 genes for TOV1946 and 242 genes for OVCAR5 whose deletion increased sensitivity to itraconazole (Supplementary Data S1 and S2). A total of 20 genes were common in the two screens. To identify common clinically actionable pathways in the two screens, we carried out a pathway analysis (32) on overlapping genes and found common pathways related to vesicular trafficking and the dynamics between the trans-golgi network (TGN) and late endosomal/lysosomal compartments (LE/L; Fig. 1C). To validate our CRISPR screen results, we selected two genes involved in these pathways for further analysis, *VSP54* and *C18orf8*, previously reported to have a role in lysosomal biology and dynamics.

**FIGURE 2:**
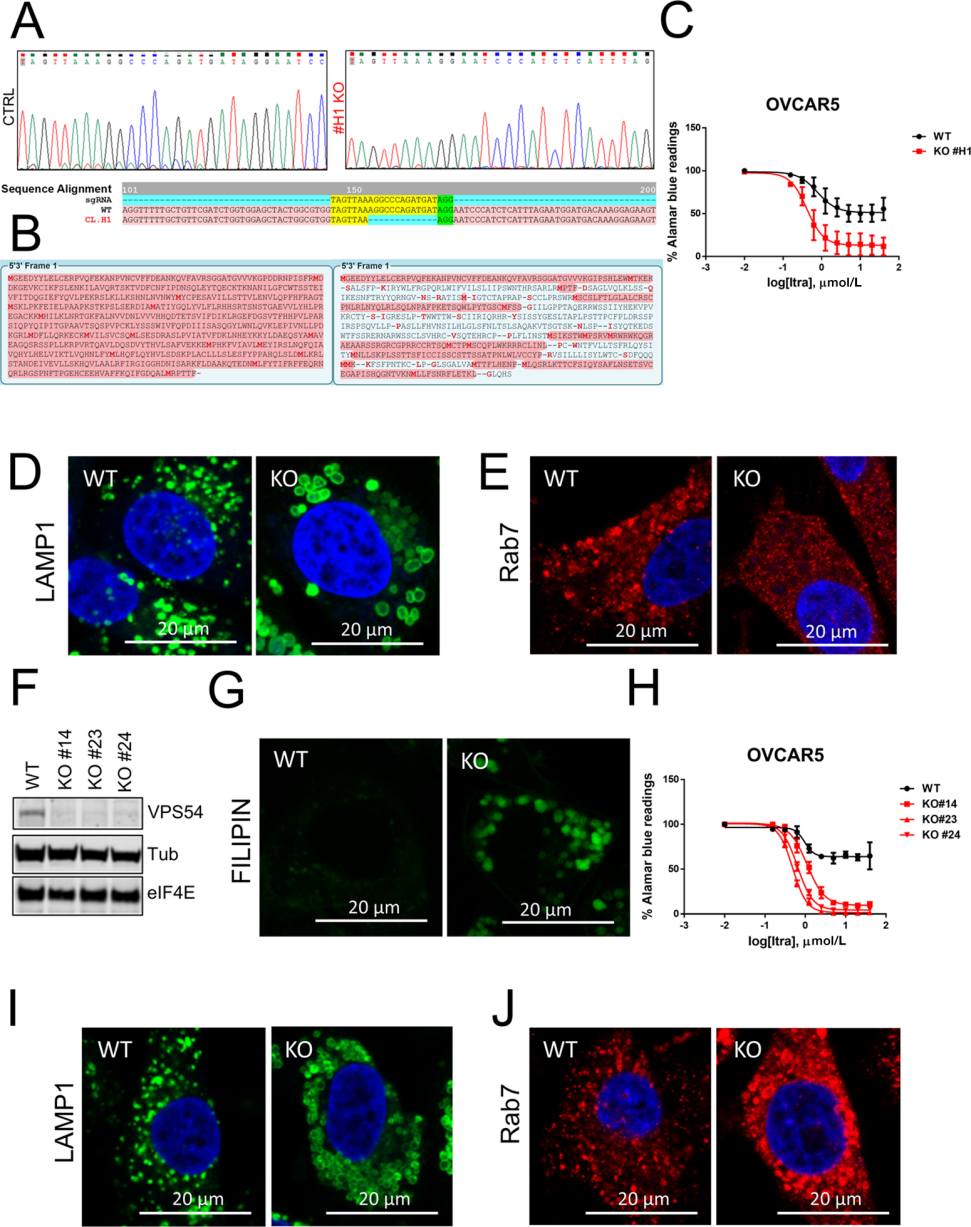
*C18orf8* and *VPS54* knockout cells are more sensitive to itraconazole (Itra). **A,** Sequencing results and alignment of PCR amplicons reporting a homozygous deletion in the exon2 of *c18orf* gene upstream the AGG PAM sequence (reported in green) in the *C18orf8* knockout clone H1. **B,** Protein translation of wild-type (left) versus knockout (right) *C18orf8*. The knockout protein sequence showed the insertion of a premature stop codon. **C,** Alamar blue results showing increased sensitivity to itraconazole in *C18orf8* knockout cells (red) compared with control (black; *n* = 3 biological replicates). **D** and **E,** LAMP1 and Rab7 staining of wild-type and *C18orf8* knockout cells. **F,** Western blotting analysis showing knockout of *VPS54* in three independent clones of OVCAR5. B-tubulin and eIF4E were used as a loading control. **G,** FILIPIN staining showing intracellular cholesterol accumulation in *VPS54* knockout cells compared with controls (#14 was used as a representative clone). **H,** Alamar blue results showing increased sensitivity to itraconazole in *VPS54* knockout cells (red) compared with controls (black; *N* = 3 biological replicates). **I–J,** LAMP1 and Rab7 staining of wild-type and *VPS54* knockout cells.

*C18orf8*, also known as *RMC1*, is a component of the CCZ1-MON1 complex that is required for recruitment of Rab7 at LE/L and lysosomal maturation. Knockdown of *C18orf8* has been shown to impair lysosomal maturation, induce lysosomal enlargement and dysfunction and inhibit autophagy (37). Consistent with the CRISPR screen, stable knockout of *C18orf8* (Fig. 2A and B; Supplementary Fig. S3A) strongly sensitized OVCAR5 and TOV1946 to itraconazole (Fig. 2C; Supplementary Fig. S3B), dramatically increased lysosomal size (as measured by LAMP1) and decreased maturation as shown by the distinct diffuse cytoplasm staining of Rab7 in knockout cells compared to the weak punctate pattern in control conditions (Fig. 2D and E; Supplementary Fig. S3C).

Vacuolar protein sorting-associated protein 54 (*VPS54*) is a component of the Golgi-associated retrograde protein (GARP) complex, which regulates retrograde transport from late endosomes to TGN and is particularly important for recycling of the mannose 6 phosphate receptor (M6PR) required for proper delivery of lysosomal proteins (38). Knockdown of GARP VPS subunits leads to cholesterol accumulation in the lysosomes as a reflection of impaired delivery to the LE/L of cholesterol transporters and lysosomal enlargement and dysfunction. Knockout of *VPS54* lead to increased sensitivity to itraconazole, cholesterol accumulation (as measured by FILIPIN staining) and lysosomal enlargement (Fig. 2F–I; Supplementary Fig. S3D–S3G). Absence of VPS54 did not influence lysosomal maturation as knockout cells showed a Rab7 dotted pattern in enlarged vesicular structures (Fig. 2J; Supplementary Fig. S3G). Taken together, these results indicate that genes involved in lysosomal flux and function can be important regulators of itraconazole resistance and impairment of these pathways can strongly sensitize ovarian cancer cells to this drug.

### Synergistic Effects of Itraconazole and the Lysosomotropic Drug Chloroquine

**FIGURE 3:**
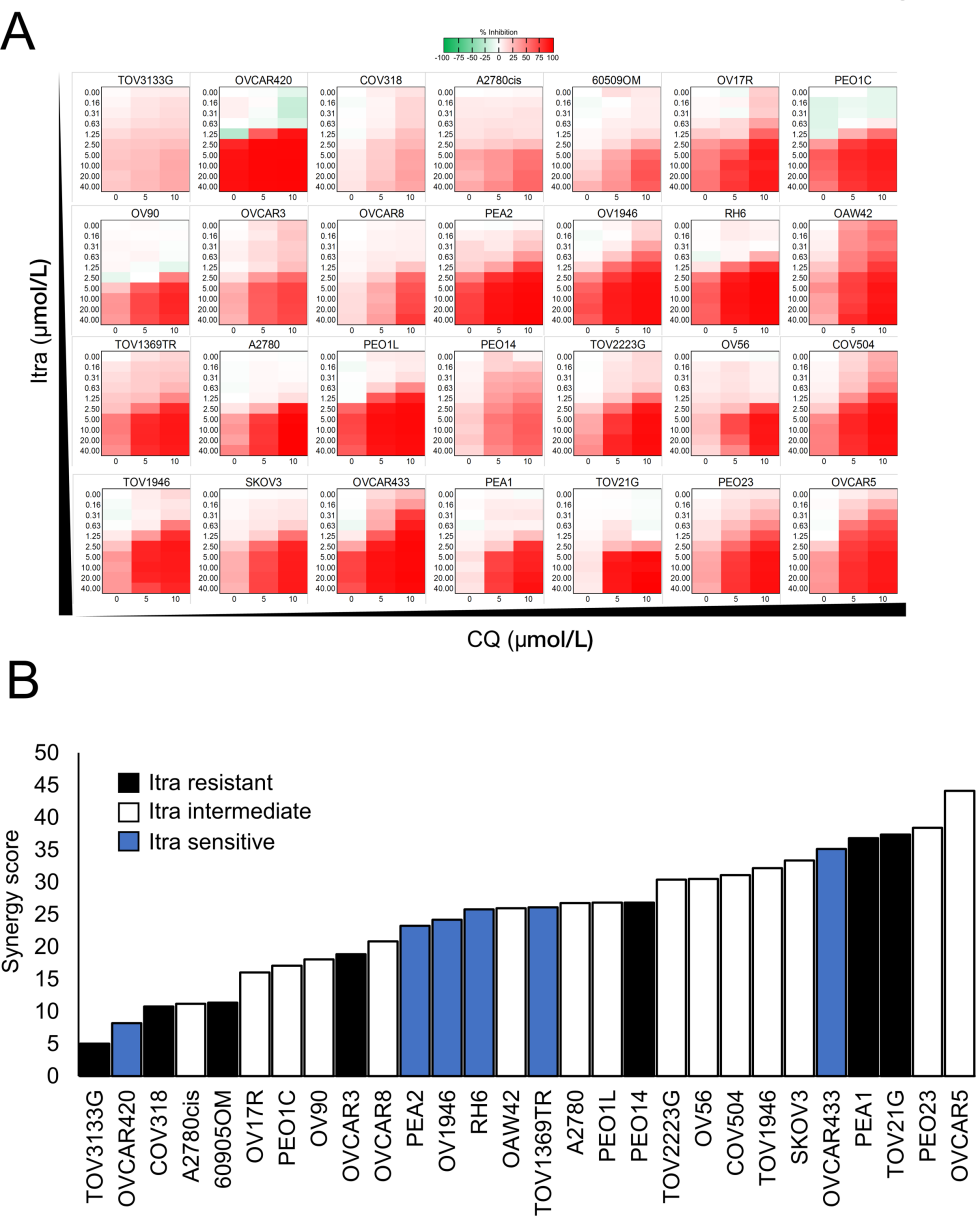
Itraconazole (Itra) synergizes with chloroquine (CQ) in a panel of ovarian cancer cell lines. **A,** Heatmaps showing inhibitory effect of itraconazole alone (0–40 μmol/L) or in combination with chloroquine (5–10 μmol/L) in a panel of 28 ovarian cancer cell lines. **B,** Waterfall plot showing the synergy scores calculated using a Bliss independence model of combinations of itraconazole and chloroquine. Synergy score values are ranked from low to high synergy (*n* = 3 biological replicates; see Supplementary Fig. S4).

We next postulated that drugs capable of phenocopying the effects of knocking out these two genes would result in an increased sensitivity to itraconazole. The antimalarial drug chloroquine and its derivative, hydroxychloroquine are drugs that have been repurposed in several cancer-related clinical trials for their anti-inflammatory properties and for targeting autophagy at lysosomal level by deacidifying lysosomal compartments (27). Similar to *VPS54* knockdown, chloroquine was shown to impair recycling of M6PR from late endosomes to TGN and to induce lysosomal enlargement and dysfunction (39). We thus tested and quantified the effects of combining itraconazole with chloroquine in the above cell lines using the Bliss synergy calculation (40). Each cell line demonstrated a different spectrum of synergistic activity of itraconazole/chloroquine combinations, ranging from low to high synergy (Fig. 3A and B, Supplementary Fig. S4) with TOV1946 and OVCAR5 previously used in itraconazole sensitivity CRISPR screen, being among the cells that showed the highest levels of synergy (Fig. 3B; Supplementary Fig. S4). Overall, 5 and 10 μmol/L chloroquine concentration were not toxic (Supplementary Fig. S5A).The itraconazole/chloroquine cytotoxic effect was due to an increase in apoptotic rate in itraconazole/chloroquine-treated cells (Supplementary Fig. S5B). Moreover, cell lines such as TOV21G which were exquisitely resistant to itraconazole alone (Fig. 1), become very sensitive to the combination with itraconazole and chloroquine (Fig. 3B; Supplementary Fig. S4).

### CRISPR Validation

As expected, the itraconazole/chloroquine combined effect was observed but significantly reduced in *C18orf8* and *VPS54* knockout cell lines (OVCAR5 and TOV1946) compared with controls (Supplementary Fig. S6A–S6D).

To better understand the biological effects of cotreatment with itraconazole and chloroquine, we compared lysosomal function and pattern in selected cell lines that displayed high levels of Bliss defined synergy (TOV21G, TOV1946, OVCAR5) or were resistant to the combination (COV318, TOV31333G; Fig. 3). Lysosomal pattern and morphology was assessed by examining LAMP1, an abundant lysosomal membrane protein that localizes to lysosomes and late endosomes (21, 23) while lysosomal function was assessed using a lysosomal intracellular functional activity assay based on a self-quenching substrate that becomes fluorescent once internalized and exposed to the lysosomal enzymatic activity (41).

**FIGURE 4:**
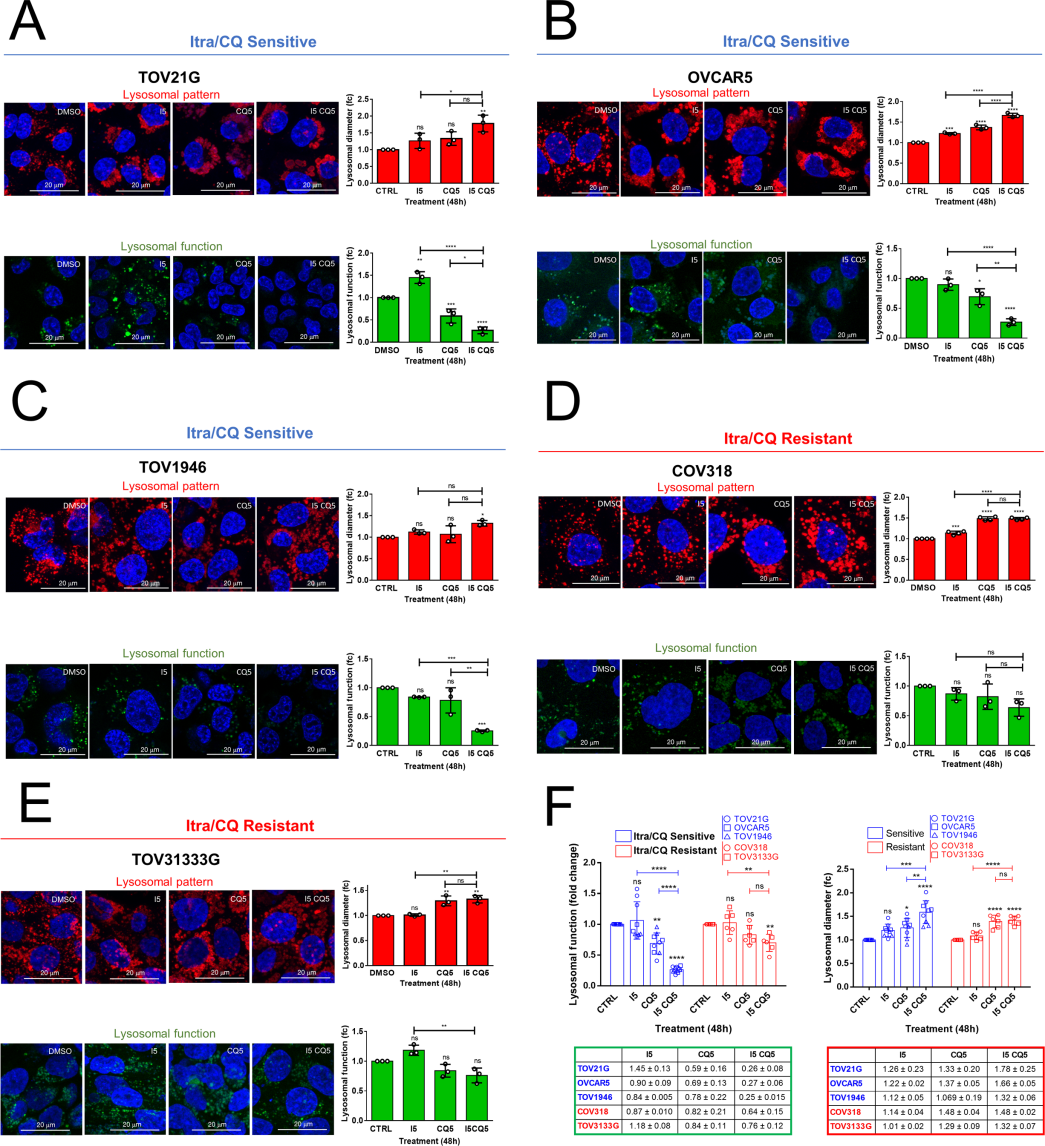
Itraconazole/chloroquine (Itra/CQ) combination induces lysosomal dysfunction in sensitive cells. **A–E,** Top, representative pictures showing analysis of lysosomal pattern by LAMP1 immunofluorescence and relative quantification of lysosomal size based on lysosomal diameter of itraconazole/chloroquine–sensitive (TOV21G, OVCAR5, and TOV1946) and -resistant cells (COV318 and TOV3133G) treated with itraconazole 5 μmol/L (I5) ± chloroquine 5 μmol/L (CQ5; red histograms, each dot represents a single experiment). Bottom, representative pictures showing analysis of lysosomal function and relative quantification of treated cells from three independent experiments (green histograms). **F,** Graph and table showing the change in lysosomal function and lysosomal size of itraconazole/chloroquine–sensitive (blue) and -resistant (red) cells treated with I5 ± CQ5. Each dot represents respectively the median value of lysosomal fluorescence and lysosomal diameter value distribution from independent experiments (*n* = 3 biological replicates). ^*^, *P* < 0.05; ^**^, *P* < 0.01; ^***^, *P* < 0.001; ^****^, *P* < 0.0001; ns, not significant.

In all cell lines, we observed a chloroquine-induced lysosomal enlargement and a trend toward a reduction in lysosome function. However, only in itraconazole/chloroquine-sensitive cells, did we observe a combined effect in lysosomal enlargement induction in parallel with a significantly reduced lysosomal function (Fig. 4A–C). In contrast, in the resistant cell lines (COV318, TOV3133G), we found that despite drug treatment, the cell lines retained more than 60% of lysosomal function (Fig. 4D and E), with no significant difference of itraconazole/chloroquine combination compared with chloroquine treatment. We further analyzed these observations (Fig. 4F) and applied mixed-effect modeling (Supplementary Tables S1 and S2) that demonstrated significant changes across all comparisons except for lysosome function between itraconazole and DMSO control.

Taken together, these results indicate that there is an association between cytotoxicity and degree of lysosomal dysfunction induced by the combination of itraconazole/chloroquine *in vitro*. To explore the safety and utility of this drug combination, we undertook a phase I clinical trial.

## Phase I Clinical Trial (HYDRA-1, NCT03081702)

### Baseline Demographics

Between 2017 and 2019, 13 patients were enrolled and two withdrew consent to participate prior to the initiation of the therapy. Median age was 54 (range, 44–77). Of the 11 patients, 10 were evaluable for efficacy. Histology was high-grade (91%, *N* = 10) and low-grade (9%, *N* = 1) serous ovarian cancer, and median prior lines of systemic therapy was seven (range, 3–9).

### Dosage, Safety, and Clinical Activity

**FIGURE 5:**
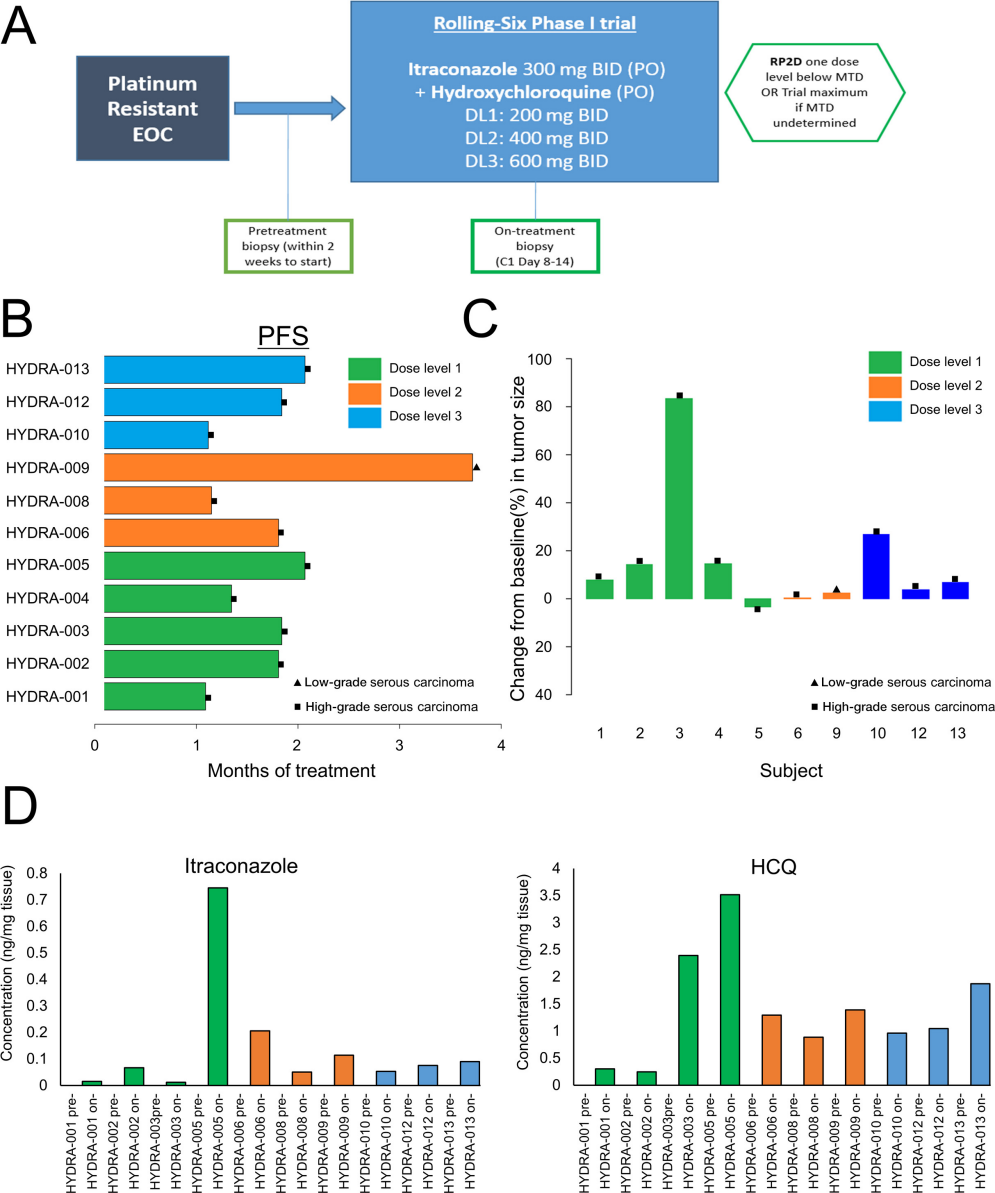
Itraconazole/hydroxychloroquine (HCQ) combination employed in a phase I clinical trial. **A,** Phase I rolling-six study design. EOC, epithelial ovarian cancer; BID, twice daily; DL, dose level; DLT, dose-limiting toxicity; PO, orally. **B,** Swimmer plot showing PFS in patient enrolled in the HYDRA clinical trial. Different dose levels are reported in green (DL1), orange (DL2), and blue (DL3). Different types of tumor are labeled with a triangle (low-grade serous carcinoma) or a square (high-grade serous carcinoma). **C,** Graph showing change (%) from baseline in tumor size in HYDRA patients according RECIST 1.1. **D,** Graphs showing intratumor detection and quantification of itraconazole (left) and chloroquine (CQ; right) in HYDRA patients pretreatment and on-treatment. Measurements were done using HPLC/MS-MS method.

Five patients were enrolled at DL1, and three at DL2 and DL3, and all were evaluable for DLTs (Fig. 5A and B; Supplementary Table S3). Median duration of therapy was 1.8 months (DL1), 1.5 months (DL2), 1.5 months (DL3). The most frequent treatment-related toxicity was nausea in 36% of the patients (grade 1), followed by diarrhea, vomiting, fatigue, and dry skin in 27% of patients (grade 1–2; Supplementary Table S4 and S5). Other grade ≥3 adverse events were grade 3 hypokalemia and grade 4 QTc prolongation (in 1 patient, DL3). A DLT, based on the protocol definition, was seen in DL2 defined as grade 3 hypertension, which resolved with appropriate oral blood pressure medication. Given that the DLT was considered manageable with optimal treatment, it was deemed safe to escalate to DL3. There were no treatment discontinuations due to toxicity. Treatment was held in 1 patient due to intolerable grade 2 fatigue and grade 2 muscle weakness without creatine kinase elevation (DL1). The recommended phase II dose was itraconazole 300 mg twice daily and hydroxychloroquine 600 mg twice daily (DL3).

No objective responses or CA125 responses according to Gynecological Cancer Intergroup criteria, were seen. Median cycles of treatment per patient was two (range, 2–4). Median PFS was 1.6 months (95% confidence interval, 1–1.7 months; Fig. 5B and C), and 1 patient with low-grade serous histology had stable disease for 3.7 months.

### Pharmacokinetic Tumor Assessment

Pretreatment and on-treatment biopsies (baseline, at cycle 1 day14) were available for 10 patients. The highest concentrations of both drugs were detected in 1 patient, HYDRA-005 that received treatment at DL1, with an intratumoral concentration of 0.745 ng/mg of tissue for itraconazole and 3.5 ng/mg tissue for hydroxychloroquine (Fig. 5D; Supplementary Fig. S7A).

### Pharmacodynamic Tumor Assessment

**FIGURE 6:**
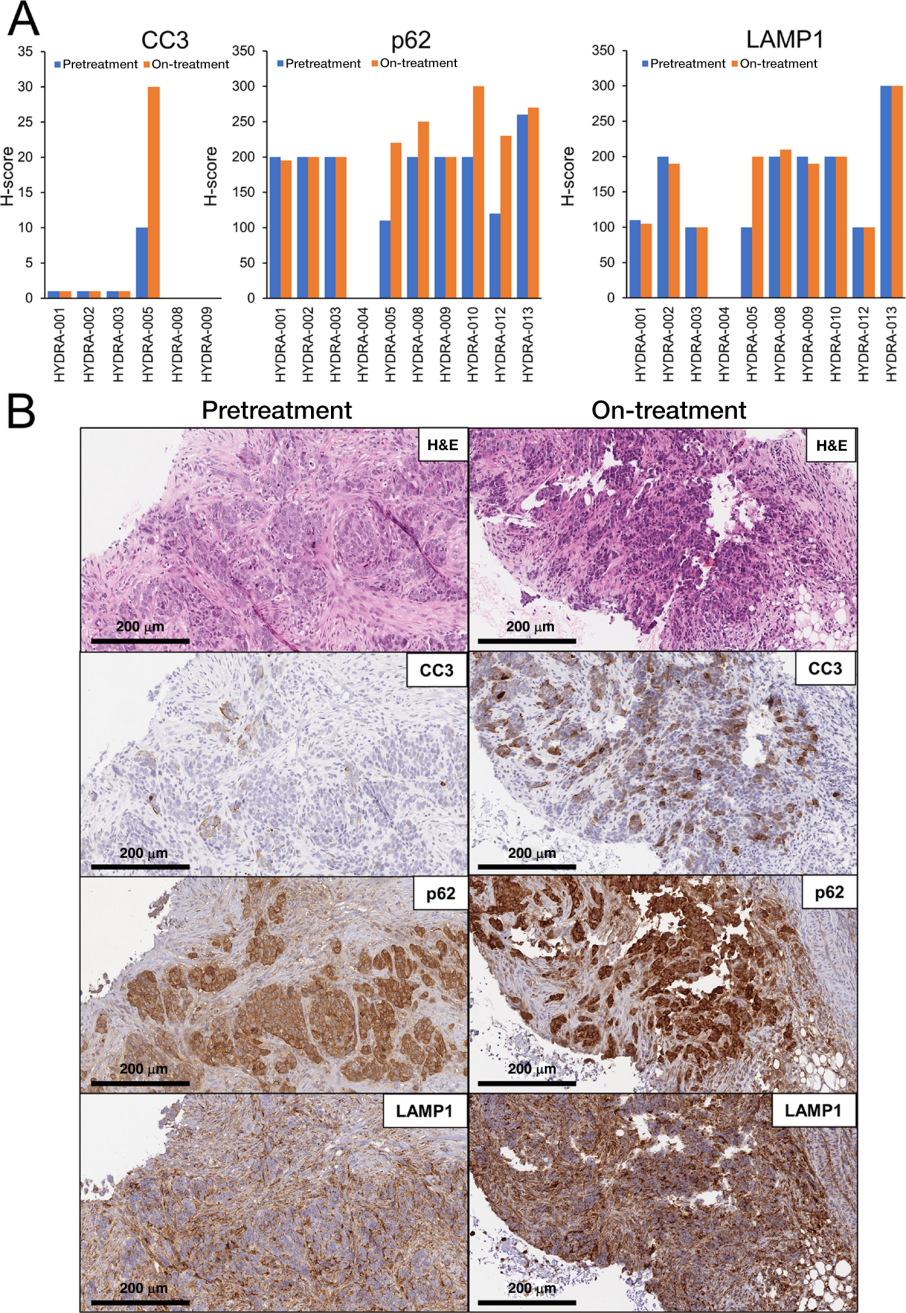
Itraconazole/chloroquine combined effect on apoptosis induction, p62 and LAMP1 accumulation. **A,** graphs showing IHC quantification of CC3, p62, and LAMP1 in HYDRA patients slides pretreatment and on-treatment (respectively, blue and orange). **B,** Representative pictures of IHC staining [hematoxylin–eosin (H&E), CC3, p62, and LAMP1] in patient HYDRA-005 pretreatment and posttreatment.

Pretreatment and on-treatment biopsies were available for IHC analysis in 10 patients. No significant changes were detected in the overall population in terms of IHC markers (autophagy, apoptosis, and lysosomal markers), morphology, mitosis, and proliferation index (Ki-67). To explore the pharmacodynamic effect of the drugs, we stained the tumor sections for LAMP1, as well as p62 (a marker that correlates with impaired autophagy). The expression of LAMP1 increased in 1 patient (HYDRA-005) in the on-treatment biopsy compared with the baseline biopsy. This patient also had an increase in the autophagy-related protein (p62), previously shown to accumulate in tumors treated with chloroquine (42), and apoptosis-related protein (CC3; Fig. 6). Interestingly, this patient had higher intratumor drug concentrations, and a decrease in size in the target lesions (Fig. 5C and D; Supplementary Fig. S7A); however, there was no correlation with clinical benefit. In addition, in all patients nonsignificant differences were observed in Ki-67 staining in tumor tissues (Supplementary Fig. S7B).

## Discussion

Despite recent advances, there remains a need for platinum resistant and refractory EOC. In this article, we present a proof-of-concept study of itraconazole and chloroquine/hydroxychloroquine in the treatment in EOC from *in vitro* to phase I clinical trial. Thereby, we identified a novel mechanism to target lysosomal homeostasis as a potential target in the treatment of malignancy.

Briefly, based on previous studies showing the beneficial effects of itraconazole in patients with EOC (5, 18, 19), we identified and validated synthetically lethal genes involved in vesicular trafficking and dynamic exchanges between TGN and lysosomes such as *C18orf8* as well as members of the GARP complex (*VPS51*, *VPS52*, *VPS53*, and *VPS54*). Extrapolating from similar documented phenotypes, we hypothesized that the antimalarial lysosomotropic drugs (chloroquine, and its derivative hydroxychloroquine), would be synthetically lethal with itraconazole. We observed a synergistic effect in several cell lines, including those with resistance to itraconazole alone (Fig. 3; Supplementary Fig. S4). Although there was no association with synergy with clinical characteristics such as platinum sensitivity, we did correlate the cytotoxic activity of the combination with the extent of relative inactivation of the lysosome through a lysosomal functional assay.

To validate our preclinical findings, we conducted a phase I dose-escalation study assessing the combination of itraconazole and hydroxychloroquine in platinum-resistant or -refractory EOC. This combination was safe at the determined dose but did not lead to clinically significant antitumor activity, suggesting that further optimization of both pharmacodynamics and pharmacokinetics may be required to realize the potential of the preclinical findings.

The limitations of both our preclinical and clinical studies are significant. Preclinically, we were limited in access to EOC models such as three-dimensional spheroids and organoids (43). We were unable to define the crucial enzymatic functions of the lysosome that were impaired by the combination of itraconazole and hydroxychloroquine, and which one of the pleiotropic activities of itraconazole is involved in the synergistic response. Of note, itraconazole can target lysosomes by binding and inhibiting the cholesterol transporter NPC1 (14), suggesting a possible role of itraconazole in contributing to lysosomal dysfunction. Moreover, cationic amphiphilic drugs which include chloroquine/hydroxychloroquine, have been widely reported to impair the action of lysosomal enzymes, induce a phenomena of aberrant phospholipid accumulation in lysosomes named phospholipidosis, and result in lysosomal enlargement and laminar body inclusions that are associated with lysosomal dysfunction (44). It is thought that subsequent cytotoxicity is related to the release of lysosomal cathepsins (45). Interestingly, some azoles like itraconazole have also been reported to induce a similar lysosomal effect (46) and it may be the combined effect of both drugs accounts for the synergy in cytotoxicity that we observed. Future studies, including gene expression profile of cells treated with the two drugs, alone or in combination, will be aimed at better characterizing the exact mechanisms of synergy, cell death and *de novo* or acquired resistance.

Despite its limitations, our preclinical data demonstrated the utility of a bedside-to-bench approach, by analyzing the potential of clinically identified drug (itraconazole) then revalidating the findings in the laboratory to understand the mechanism of action and redevelop them. Although clinical activity was not seen, the women on trial were heavily pretreated with a median of seven prior lines of treatment, materially limiting the possibility they may respond to the novel, nontoxic combination. In addition, (excluding patient 005), generally the tissue concentration of itraconazole/hydroxychloroquine was insufficient to achieve an antitumor effect at all dose levels, albeit assayed early at day 14 given that itraconazole is thought to accumulate in tissue (47). Moreover, bioavailability studies have shown that hydroxychloroquine steady-state levels are reached after 6 months of therapy (48). At the time of study design, the early timepoint was thought to reflect the need to ensure that most women remained on study at the time of biopsy.

Importantly, the drug combination was well tolerated, with the most common adverse events being predicted from the mechanisms of action of both itraconazole and hydroxychloroquine. For example, the inhibition of 11β‐hydroxysteroid dehydrogenase 2 leading to hypertension is a well-known dose-related toxicity of itraconazole (49). Other common toxicities such as diarrhea and liver function abnormalities were generally of low grade and reversible (5). There remains the need to develop pharmacologic formulation and develop predictive biomarkers of activity to thoroughly evaluate the potential of this drug combination to offer women a nonchemotherapeutic avenue to treat EOC. Given the absence of clinical activity detected in this population in the phase I trial, a phase II trial was not conducted.

In conclusion, using drug repurposing, we identified a combination of FDA-approved drugs that have a preclinical therapeutic potential in EOC, likely by targeting lysosomal function and affecting pathways associated with chemoresistance. Lysosomal targeting for cancer has been considered for nearly 40 years (50), although was thought to have limited therapeutic ratio until recently (51). Our work highlights the difficulties in drug repurposing and offers a template for future research into lysosomal targeting in EOC to validate this as tractable target for cancer therapy.

## Supplementary Material

Supplementary Materials and Methods - List of cell lines
- Lentiviral constructs, lentivirus generation and infection
- Apoptosis Assay
- FILIPIN staining
- Lysosomal assay and immunofluorescence

Clinical Trial Protocol Detailed protocol of the HYDRA-1 (NCT03081702) Trial. TITLE: A phase I/II trial investigating the tolerability, toxicity and efficacy of hydroxychloroquine and itraconazole in patients with advanced platinum-resistant epithelial ovarian cancer (EOC) (HYDRA-1 study).

Supplementary Figures 1-7 Supplementary Figure 1: Itra dose response in ovarian cancer cell lines. - Supplementary Figure 2: Analysis on ovarian cancer cell lines used in the Itra-sensitizing CRISPR screen (OVCAR5 and TOV1946). - Supplementary Figure 3: c18orf8 and VPS54 Knockout effects on the TOV1946 cell line. - Supplementary Figure 4: Itra+CQ dose responses. - Supplementary Figure 5: 5/10 uM CQ response and apoptosis assay results. - Supplementary Figure 6: Itra+CQ dose response in c18orf8 and VPS54 knockout cells and calculation of synergy scores. - Supplementary Figure 7: Intratumoural Itra/CQ detection and Ki-67 analysis in patient samples.

Supplementary Tables 1-5 Supplementary Tables 1 and 2 show respectively the fold change of lysosomal function and size in treated sensitive/resistant cells obtained by mixed effect modelling. Supplementary Table 3 shows patients enrolled per dose-level (DL) and dose limiting toxicities (DLTs). Supplementary Tables 4 and 5 show the summary of all and treatment related adverse effects.

Supplementary File 1 CRISPR screen OVCAR5. Results of the Itra sensitizing CRISPR screen on the OVCAR5 cell line using drugZ algorithm.

Supplementary File 2 CRISPR screen TOV1946. Results of the Itra sensitizing CRISPR screen on the TOV1946 cell line using drugZ algorithm.
