# Supplementary material for: Repurposing Itraconazole and Hydroxychloroquine to Target Lysosomal Homeostasis in Epithelial Ovarian Cancer
Source: Cancer Res Commun. 2022 May 4;2(5):293–306. doi: 10.1158/2767-9764.CRC-22-0037 (PMC9981200; doi:10.1158/2767-9764.CRC-22-0037)
Supplement: Supplementary Materials and Methods — - List of cell lines - Lentiviral constructs, lentivirus generation and infection - Apoptosis Assay - FILIPIN staining - Lysosomal assay and immunofluorescence [file crc-22-0037-s01.docx]

**Supplementary Materials and Methods**

**List of cell lines**

| **CELL LINE**^1^ | **RRID** | **ORIGINAL SOURCE** | **LAST STR authentication** |
| --- | --- | --- | --- |
| 60905OM | CVCL_4065 | Ben Neel | 30-Oct-20 |
| A2780 | CVCL_0134 | Graham Fletcher | 30-Oct-20 |
| A2780CIS | CVCL_1942 | Graham Fletcher | 30-Oct-20 |
| COV318 | CVCL_2419 | HPACC | 30-Oct-20 |
| COV504 | CVCL_2424 | HPACC | 4-Dec-21 |
| OAW42 | CVCL_1615 | ATCC | 30-Oct-20 |
| OV17R | CVCL_2672 | HPACC | 30-Oct-20 |
| OV1946 | CVCL_4375 | Anne-Marie Mes-Massons | 14-Jul-20 |
| OV56 | CVCL_2673 | HPACC | 30-Oct-20 |
| OV90 | CVCL_3768 | Patricia Tonin | 18-Aug-21 |
| OVCAR3 | CVCL_0465 | ATCC | 14-Jul-20 |
| OVCAR420 | CVCL_3935 | Gordon Mills | 18-Aug-21 |
| OVCAR433 | CVCL_0475 | Gordon Mills | 18-Aug-21 |
| OVCAR5 | CVCL_1628 | Gordon Mills | 30-Oct-20 |
| OVCAR8 | CVCL_1629 | Gordon Mills | 14-Jul-20 |
| PEA1 | CVCL_2682 | James Brenton | 18-Aug-21 |
| PEA2 | CVCL_2683 | James Brenton | 18-Aug-21 |
| PEO14 | CVCL_2687 | James Brenton | 18-Aug-21 |
| PEO1C | CVCL_2686 | James Brenton | 18-Aug-21 |
| PEO1L | CVCL_2686 | James Brenton | 18-Aug-21 |
| PEO23 | CVCL_2689 | James Brenton | 18-Aug-21 |
| RH6 | Derivative of OV90^2,3^ | Patricia Tonin | 30-Oct-20 |
| SKOV3 | CVCL_0532 | ATCC | 6-Dec-21 |
| TOV1369TR | CVCL_9T17 | Anne-Marie Mes-Massons | 18-Aug-21 |
| TOV1946 | CVCL_4062 | Anne-Marie Mes-Massons | 30-Oct-20 |
| TOV21G | CVCL_3613 | Anne-Marie Mes-Massons | 30-Oct-20 |
| TOV2223G | CVCL_4063 | Anne-Marie Mes-Massons | 11-Nov-21 |
| TOV3133G | CVCL_4064 | Anne-Marie Mes-Massons | 14-Jul-20 |
| HEK293T | CVCL_0063 | ATCC | 25-Feb-22 |

**Lentiviral constructs, lentivirus generation and infection**

Lenti‐Cas9‐2A‐Blast (Plasmid #73310) was purchased from Addgene. Lentivirus containing control LacZ and sgRNA targeting the essential genes PSMB1 and PSMD2 coding for proteosomal subunits were kindly provided by the Princess Margaret Genomic Centre (Toronto, ON, Canada). Lentiviral particles were generated by co-transfection of 293T cells with packaging plasmids psPAX2 (Addgene #12260) and pMD2.G (Addgene #12259) together with Lenti‐Cas9‐2A‐Blast (Addgene #73310) using Lipofectamine 3000 (Thermo Fisher Scientific) according to the manufacturer’s instructions. Virus supernatant was harvested 48- and 72-hours post-transfection. Cell lines were transduced with lentiviral supernatant in the presence of 8 μg/ml polybrene. Infected cells were selected for 48 hrs in puromycin containing media (OVCAR5 3.5 μg/ml and TOV1946 2 μg/ml) or 7 days in 5 μg/ml blasticidin containing media. Lentiviral particles used to knock out *c18orf8* and *VPS54* were purchased from Horizon discovery (Edit-R Human All-in-one Lentiviral *c18orf8* and *VPS54* sgRNA, #VSGH11936-247734196 and #VSGH11936-247699202 respectively) and cells were infected according to the manufacturer’s instructions.

**Apoptosis Assay**

Apoptosis was measured using the Incucyte Caspase-3/7 Green Dye. The dye was added to the treated cells and images were acquired after three days with confocal microscope (Zeiss LSM700). Nuclei were labeled with Nucblue (Hoechst) according to the manifacturer’s instructions. Calculation of the apoptotic rate was done using Fiji software^4^.

**FILIPIN staining**

20 x10^3^ cells were plated onto 8 well cell imaging coverglass chamber slides (Eppendorf). After 48 hours of treatment cells were washed 3 times with PBS and fixed with freshly prepared 4% paraformaldehyde for 20 minutes at room temperature followed by 10 minutes incubation with 20 mM Ammonium Chloride in PBS for 10 min to quench the paraformaldehyde. After 3 washes with PBS cells were stained with FILIPIN (Sigma-Aldrich) at a final concentration of 50 ug/ml for 1 h RT. After rinsing cells images were taken using confocal microscope (Zeiss LSM700) equipped with an UV laser.

**Lysosomal assay and immunofluorescence**

Lysosomal function was performed using the Lysosomal Intracellular Activity Assay Kit (Biovision) according to the manifacturer’s instructions. Briefly, cells were plated onto 8 well cell imaging coverglass chamber slides, let adhere overnight and treated with drugs for 48 hours. Then medium was removed and replaced with 0.5% FBS medium with or without drugs supplemented with Self-Quenched Substrate for 3 hours in the incubator. Nuclei were stained incubating cells for 5 minutes with Hoechst 33342 dye (NucBlue® Live Ready Probes, Thermo Fisher Scientific) and washed twice with the Lysosomal Assay Buffer. Images were taken using confocal microscope (Zeiss LSM700) with 488 nm excitation filter and fluorescence intensity was calculated using Fiji software^4^.

For LAMP1 staining cells were blocked and permeabilized with 0.5% BSA and 0.1% saponin in PBS for 1 hour and incubated overnight with primary antibody (LAMP1, Cell Signaling Technology #9091) in blocking Buffer. After incubation with Alexa-Fluor647 conjugated anti-rabbit (Thermo Fisher Scientific) for 1 hour and with DAPI (Thermo Fisher Scientific) 1µg/ml for 5 minutes cells were washed with PBS and analyzed by confocal microscopy. Lysosome area was calculated using the Fiji particle analysis algorithm, software^4^ and lysosomes were approximated as circles. In both experiments at least 50 cells per condition were analyzed per single experiment. All experiments were repeated independently 3 times.

**References:**

1. Medrano M, Communal L, Brown KR, et al. Interrogation of Functional Cell-Surface Markers Identifies CD151 Dependency in High-Grade Serous Ovarian Cancer. *Cell Rep*. 2017;18(10):2343-2358.

2. Cody, N. A. L. *et al.* Transfer of chromosome 3 fragments suppresses tumorigenicity of an ovarian cancer cell line monoallelic for chromosome 3p. *Oncogene* **26**, 618–632 (2007).

3. Al Habyan, S., Kalos, C., Szymborski, J. & McCaffrey, L. Multicellular detachment generates metastatic spheroids during intra-abdominal dissemination in epithelial ovarian cancer. *Oncogene* **37**, 5127–5135 (2018).

4. Schindelin, J. *et al.* Fiji: an open-source platform for biological-image analysis. *Nat. Methods* **9**, 676–682 (2012).
