# Supplementary material for: Repurposing Itraconazole and Hydroxychloroquine to Target Lysosomal Homeostasis in Epithelial Ovarian Cancer
Source: Cancer Res Commun. 2022 May 4;2(5):293–306. doi: 10.1158/2767-9764.CRC-22-0037 (PMC9981200; doi:10.1158/2767-9764.CRC-22-0037)
Supplement: Clinical Trial Protocol — Detailed protocol of the HYDRA-1 (NCT03081702) Trial. TITLE: A phase I/II trial investigating the tolerability, toxicity and efficacy of hydroxychloroquine and itraconazole in patients with advanced platinum-resistant epithelial ovarian cancer (EOC) (HYDRA-1 study). [file crc-22-0037-s02.pdf]

Protocol #: HYDRA-1
Version / Date: Amendment 1 / 17 Nov 2017

TITLE: A phase I/II trial investigating the tolerability, toxicity and efficacy of hydroxychloroquine and itraconazole in patients with advanced platinum-resistant epithelial ovarian cancer (EOC) (HYDRA-1 study)

Coordinating Center: Drug Development Program
Princess Margaret Cancer Centre

Principal Investigator: Dr. Stephanie Lheureux
700 University Ave.
Toronto, Ontario, M5G 2M9
Telephone: 1-416-946-2818
Fax: 1-416-946-4467
stephanie.lheureux@uhn.ca

Co-Investigators: Dr. Amit Oza
700 University Ave
Toronto, Ontario, M5G 2M9
Telephone: 1-416-946-2818
Fax: 1-416-946-4467

Dr. Neesha Dhani
700 University Ave
Toronto, Ontario, M5G 2M9
Telephone: 1-416-946-2260
Fax: 1-416-946-6546

Dr. Marcus Butler
700 University Ave
Toronto, Ontario, M5G 2M9
Telephone: 1-416-946-4585
Fax: 1-416-946-6546

Research Fellows: **Ilaria Colombo (primary)**
700 University Ave.
Toronto, Ontario, M5G 2M9
Telephone: 1-416-946-2818
Fax: 1-416-946-4467
ilaria.colombo@uhn.ca

Dr. Sunu Cyriac
700 University Ave.
Toronto, Ontario, M5G 2M9
Telephone: 1-416-946-2818
Fax: 1-416-946-4467

Dr. Yeh Chen Lee
700 University Ave.
Toronto, Ontario, M5G 2M9
Telephone: 1-416-946-2818
Fax: 1-416-946-4467

Dr. Luisa Bonilla
700 University Ave.
Toronto, Ontario, M5G 2M9
Telephone: 1-416-946-2818
Fax: 1-416-946-4467

Affiliate Scientist: Dr. Anthony Joshua
Princess Margaret Hospital
610 University Ave.
Toronto, Ontario, M5G 2M9
anthony.joshua@uhn.ca

Dr. Bradly Wouters
610 University Ave.
Toronto, Ontario, M5G 2M9
bwouters@uhnresearch.ca

Statistician:
Lisa Wang
610 University Ave. Suite 10-507
Toronto, Ontario
M5G 2M9
Telephone: 1-416-946-4501 ext: 4883
Fax: N/A
Lisa.wang@uhnresearch.utoronto.ca

Study Coordinator:
Avalon D’souza
700 University Ave, 7th floor
Toronto, Ontario
M5G 1X6
Telephone: (416) 946-4501 ext. 3910
Fax: 416.946.2016
Email: avalon.dsouza@uhn.ca

Investigational Agent(s): Itraconazole (Janssen Pharmaceuticals), Hydroxychloriquine (Apotex Pharmaceuticals)

SCHEMA

Part 1: Rolling six phase I cohort

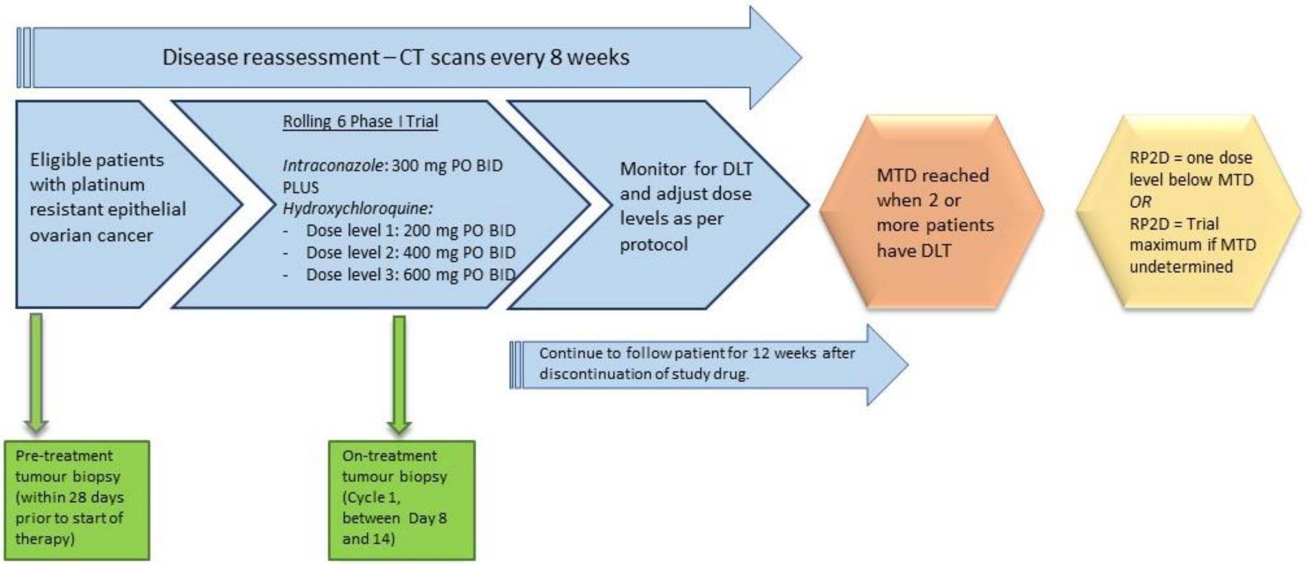

Part 2: Simon 2-stage phase II cohort

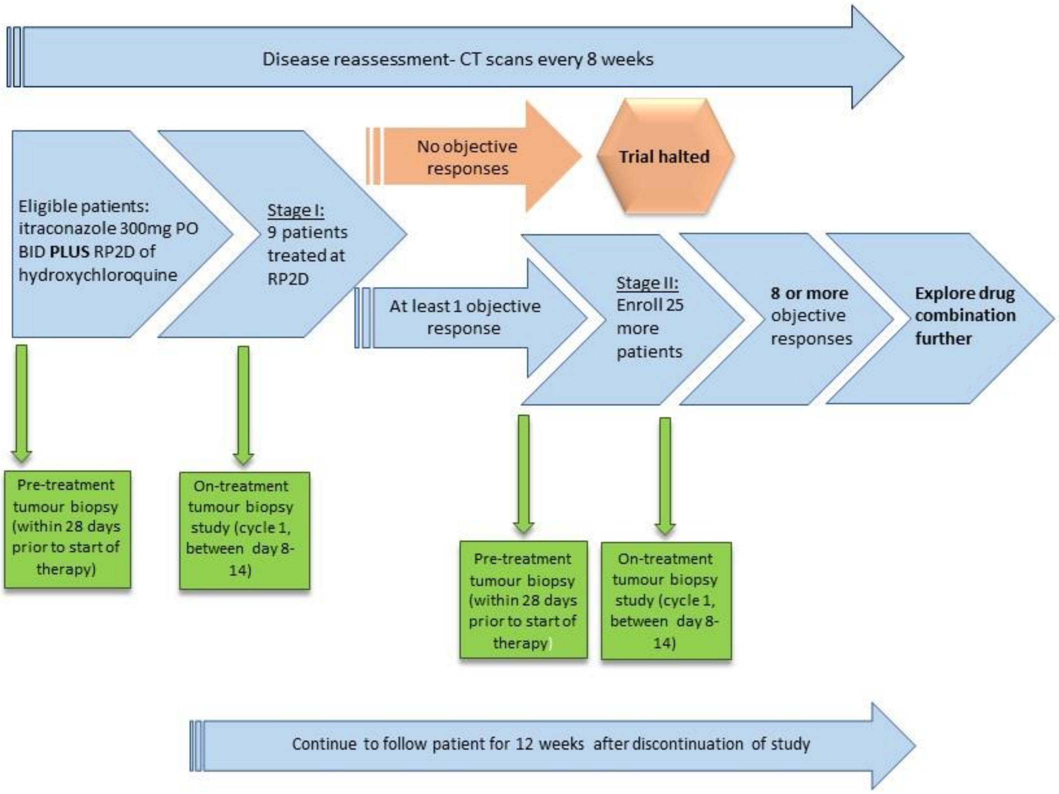

SYNOPSIS

Title of study:**:** A phase I/II trial investigating the tolerability, toxicity and efficacy of hydroxychloroquine and itraconazole in patients with advanced platinum-resistant epithelial ovarian cancer (EOC) (HYDRA-1 study)
Objectives:<br>**Primary Objectives**<br>• To determine the maximum tolerated dose or maximum administered dose (if no toxicity observed) of combination therapy of itraconazole and hydroxychloroquine for the recommended phase 2 cohort dose.<br><br>**Secondary Objective**s<br>• To investigate the overall response rate (ORR) with combination itraconazole and hydroxychloroquine in platinum-resistant epithelial ovarian cancer (EOC).<br>• To investigate the median progression-free survival (PFS) with combination itraconazole and hydroxychloroquine in platinum-resistant epithelial ovarian cancer (EOC).<br>•
Study Design: Phase I- rolling 6 phase I design<br>Phase II- Simon 2-stage phase II design
Number of patients:<br>Phase I: 6-18 patients<br>Phase II: 9-34 patients
<u>Main inclusion criteria:</u><br>• Patients aged 18 years or older with histologically or cytologically confirmed epithelial ovarian cancer.<br>• Platinum-resistant or refractory disease defined as a radiological or clinical progression less than six months after having finished platinum-based chemotherapy.<br>• ECOG performance status equal to or less than 1.<br>• Patients must have clinically or radiographically documented measurable disease.

<u>Main exclusion criteria:</u><br><br>• Patients who have not recovered (≤ grade 1) from adverse events related to previous treatments.<br>• Patients with any other prior malignancy from which the patient has been disease free for less than 3 years, with the exception of adequately treated and cured basal or squamous cell skin cancer, superficial bladder cancer, carcinoma in situ of any site or any other cancer.<br>• Patients with a known G6PD deficiency due to the risk of hemolytic anemia with the use of hydroxychloroquine.<br>• Patients with known retinopathy due to the risk of worsening retinopathy with hydroxychloroquine.<br>• Uncontrolled intercurrent illness including, but not limited to, ongoing or active infection, symptomatic or asymptomatic congestive heart failure, unstable angina pectoris, cardiac arrhythmia, or psychiatric illness/social situations that would limit compliance with study requirements.<br>• Patients with chronic Hepatitis B or hepatitis C infections or HIV<br>• Patients who already have a clinical indication for treatment with itraconazole (e.g. chronic candidiasis or other fungal infection) or hydroxychloroquine (e.g. lupus).
Intervention:<br>Itraconazole 300 mg PO BID daily x 28 day cycle with hydroxychloroquine as per dose escalation schedule x28 day cycle until progression of disease or dose-limiting toxicity
Correlatives:<br>Pre-treatment biopsies will be performed up to 28 days prior to the start of treatment. On-treatment biopsies will be obtained between days 8 and 14 of cycle 1 of treatment.<br><br>Immunofluorescence (IF) assays will be employed to examine co-localization of free cholesterol (FILIPIN stain) with late endosomes/ lysosomes (LAMP1 stain). IHC will also be employed to assess the presence of NPC1, SCP2 and STARD3 as markers of cholesterol trafficking. Bodipy lipid staining will be performed to detect levels of free and esterified cholesterol.<br><br>RNA will be extracted for a custom PCR array of genes involved in lipoprotein signaling, cholesterol transport, synthesis and metabolism, and autophagy.<br><br>IHC LC3B, p62, caspase 3 cleavage products, gamma-H2AX and Ki67 as markers of autophagy, apoptosis and proliferation. IHC for CD31 and HIF-1α will be done to investigate the role of angiogenesis.<br><br>Next generation sequencing for *mTOR, AKT-PI3K pathway* genes as well as will be performed to investigate their role in resistance and authophagy.<br><br>Different laboratories will be involved for correlatives analysis:

- Princess Margaret Cancer Centre Labs, Toronto, Ontario;<br>- University Health Network Labs, Toronto, Ontario;<br>- Ontario Institute for Cancer Research (OICR), Toronto, Ontario;<br>- Princess Margaret, OICR Translational Genomic Labs, Toronto, Ontario.
Statistics:<br>Phase II sample size is based on the Simon 2-stage phase II design taking into account the following statistics:<br><br><u>Stage 1 of accrual</u>: 9 response evaluable patients will be entered in the first stage. Using response hypotheses of H_0 <15 % and H_a >35%, we would reject the drug at the end of the first stage of accrual if no objective responses were seen. Otherwise, an additional 25 patients will be accrued. Patients treated at RP2D in the phase 1 part of the study will be considered for the statistical analysis of the phase 2 stage 1. A total (between phase 1 and phase 2 stage 1) of 9 response evaluable patients treated at RP2D are required.<br><br><u>Stage 2 of accrual</u>: An additional 25 patients will be accrued. We would accept the drug as active if 8 or more objective responses are observed from 25 patients accrued.<br><br><u>Significance level and power</u>: The procedure described above test the null hypothesis that the response rate is 15% versus alternating hypotheses that the response rate is 35%. The significance level (*i.e.*, the probability of rejecting H0 when it is true) is α=0.05 and the power (*i.e.*, the probability of deciding the regimen is active) is 0.81 when true response rate is 20%. The expected sample size with this design is 19 when the null hypothesis is true and 25 when the alternative hypothesis is true.

**Dose Escalation Schedule**		
**Dose Level**	**Dose**	
	itraconazole (mg)	***hydroxychloroquine (mg)***
Level -1	300 mg PO BID	200 mg PO OD
Level 1	300 mg PO BID	200 mg PO BID
Level 2	300 mg PO BID	400 mg PO BID
Level 3	300 mg PO BID	600mg PO BID

TABLE OF CONTENTS

SCHEMA....3

SYNOPSIS....5

1. OBJECTIVES....10
   1.1 Primary Objectives....10
   1.2 Secondary Objectives....10
   1.3 Exploratory Objectives....10

2. BACKGROUND....10
   2.1 Platinum-Resistant High Grade Serous Ovarian Cancer....10
   2.2 Background Therapeutic Information....12
   2.3 Correlative Studies Background....15
   2.4 Impact on Cancer Care....17

3. PATIENT SELECTION....18
   3.1 Inclusion Criteria....18
   3.2 Exclusion Criteria....19
   3.3 Pregnancy....20
   3.4 Inclusion of Women and Minorities....20

4. REGISTRATION PROCEDURES....20
   4.1 General Guidelines....20
   4.2 Registration Process....20

5. TREATMENT PLAN....21
   5.1 Agent Administration....21
   5.2 Definition of Dose Limiting Toxicity....22
   5.3 Rolling Six Design....23
   5.4 General Concomitant Medication and Supportive Care Guidelines....25
   5.5 Duration of Therapy....25
   5.6 Duration of Follow Up....26
   5.7 Criteria for Removal from Study....26

6. DOSING DELAYS/DOSE MODIFICATIONS....27

7. ADVERSE EVENTS....30
   7.1 List of Adverse Events and Reporting Requirements....30
   7.2 Adverse Event Characteristics....34
   7.3 Serious Adverse Event Reporting....35
   7.4 Routine Adverse Event Reporting....36
   7.5 Documentation of Adverse Events....37
   7.6 Follow-Up of AEs and SAEs....37

7.7 Pregnancy....37
7.8 Investigator Notifications....37
7.9 Data Safety and Monitoring Board....38

8. PHARMACEUTICAL INFORMATION....38
8.1 Investigational Agents....38

9. CORRELATIVE, AND SPECIAL STUDIES....41
9.1 Laboratory Correlative Studies....41

10. STUDY CALENDAR....45

11. MEASUREMENT OF EFFECT....47
11.1 Antitumor Effect – Solid Tumors....47

12. DATA REPORTING / REGULATORY REQUIREMENTS....53
12.1 Data Collection and Reporting....53
12.2 Source Documents....53
12.3 Retention of Patient Records and Study Files....53
12.4 Site and Study Closure....54

13. STATISTICAL CONSIDERATIONS....55
13.1 Study Design/Endpoints....55
13.2 Sample Size/Accrual Rate....56
13.3 Stratification Factors....57
13.4 Analysis of Secondary Endpoints....57
13.5 Reporting and Exclusions....57

REFERENCES....59

APPENDIX A PERFORMANCE STATUS CRITERIA....62

APPENDIX B INFORMATION ON POSSIBLE DRUG INTERACTIONS....63

APPENDIX C PROHIBITED DRUGS....65

APPENDIX D SAMPLE WALLET CARD....69

APPENDIX E SAMPLE PILL DIARY....70

APPENDIX F DATA MANAGEMENT GUIDELINES....72

1. OBJECTIVES

1.1 Primary Objectives

- To determine the maximum tolerated dose or maximum administered dose (if no toxicity observed) of combination therapy of itraconazole and hydroxychloroquine for the recommended phase 2 cohort dose.

1.2 Secondary Objectives

- To investigate the overall response rate (ORR) with combination itraconazole and hydroxychloroquine in platinum-resistant epithelial ovarian cancer (EOC).
- To investigate the median progression-free survival (PFS) with combination itraconazole and hydroxychloroquine in platinum-resistant epithelial ovarian cancer (EOC).

1.3 Exploratory Objectives

- Pre- and post-treatment biopsied will be performed to investigate the effect of treatment on the following:
    - Mechanism of cytotoxicity *in vivo* of combination therapy itraconazole and hydroxychloroquine
    - Effect on apoptosis/proliferation
    - Impact on cholesterol metabolism using Bodipy staining on tumour biopsy and peripheral blood mononuclear cells (PBMCs)
    - Effect on angiogenesis
    - RNA sequencing will be performed to investigate the changes of cholesterol-trafficking proteins and autophagy-related proteins with treatment

- To explore the impact of PI3K-AKT and mTOR mutations on autophagy and cholesterol-trafficking, next generation sequencing will be used on on-treatment biopsy tissue

- To prospectively explore the validity of previously identified putative biomarker genes through pre-clinical CRISPR-Cas9 drop out screens in patients undergoing treatment with itraconazole and hydroxychloroquine

- Future validation of any as yet unknown biomarkers of interest, or mechanisms of action of the combined chemotherapy that are discovered during the course of the above mentioned objectives

2. BACKGROUND

2.1 Platinum-Resistant High Grade Serous Ovarian Cancer

Since the first chemotherapeutic agents were discovered over 70 years ago, the mainstay of systemic cancer treatment remains chemotherapy. With the advent of targeted therapy, many more agents have been added to the anti-cancer therapy arsenal; however, whether it is to

chemotherapy or targeted therapy, drug resistance poses a major barrier to the treatment of advanced cancers.

Epithelial ovarian cancer (EOC) is the second most common of the gynecological malignancies, representing approximately 4-6% of all cancers in females. It is also the most lethal as it represents the 5th most common cause of cancer death in women in Canada [1]. EOC is often diagnosed at a late stage and although initial treatment with surgery and platinum chemotherapy may lead to a complete response, these cancers often relapse and are less likely to respond to further therapy. A small subset of patients will also have chemotherapy-refractory disease at the onset. Resistance to platinum chemotherapy, defined as relapse within less than six months of having completed chemotherapy, portends a poor prognosis [2]. Though chemotherapy can temporarily control disease, response rates in the platinum-resistant setting are poor and treatment is not curative.

2.1.1 Autophagy as a Mechanisms of Resistance in EOC

Among the postulated mechanisms for resistance to platinum chemotherapy is the induction of autophagy [3-5]. Autophagy is a mechanism that occurs normally in cells to help recycle old cytoplasmic components and preserve energy in a low nutrient state. It is also thought to help prevent cancer by ridding cells of damaged mitochondria, which could accumulate reactive oxygen species leading to DNA damage and carcinogenesis [6]. Monoallelic loss of the essential autophagy gene, *Beclin1*, has been found in 40 -75% of human breast, prostate, and ovarian cancers, suggesting that autophagy may play a role in preventing these tumours [7]. Yet, in EOC, it is posited that autophagy helps to sustain cancer cells [8]. A recent study showed that the levels of autophagy are significantly higher in the cisplatin resistant ovarian cancer cell line A2780 and that the combined treatment of cisplatin with the autophagy inhibitor 3-methyladenine increased the cell death. Moreover, the knockdown of *Beclin 1* was shown to increase cisplatin-induced cell death and apoptosis [9]. Autophagy inhibitors given in combination with chemotherapy have been found to suppress tumour growth and trigger cell death to a greater extent than chemotherapy alone, both in vitro and in vivo [10]. Such inhibitors include the anti-malarial drug chloroquine (CQ) and its derivative, hydroxychloroquine (HCQ). Taken together, autophagy may represent a major mechanism for chemoresistance and thus, represents a potential novel therapeutic target.

2.1.2 Cholesterol-trafficking and Metabolic Dysregulation in Cancer Cells

Cholesterol is tightly controlled in the cell as it is an important component of the cell membrane and is responsible for certain signal transduction pathways as well through its involvement with lipid rafts [11]. Cancer cells depend on de novo lipid synthesis for the generation of fatty acids to meet the energy requirements for increased tumour growth. Abnormal lipid metabolism, leading to increased lipid synthesis, is found to play an important role in the pathogenesis of malignancies, including ovarian cancer. In particular, altered lipid metabolism is detected in ovarian cancer patients during early and late stages of disease, including patients with recurrent

disease, when compared to healthy controls [3]. The triazole antifungal drug itraconazole is thought to alter the cholesterol-trafficking mechanism in the cell through inhibition of proteins such as OSBP1/2, two members of the oxysterol binding protein family (OSBPs) as well as SCP-2 and STARD3 which are responsible for recruitement of cholesterol to the autophagolysosome and cell membrane, respectively [12, 13]. Alterations in the trafficking mechanism lead to an accumulation of cholesterol in the endosomes and lysosomes, resulting in cell death [11, 14].

2.1.3 Pre-clinical Data of the Synergy of Autophagy Inhibition and Altered Cholesterol-Trafficking as a Cytotoxic Strategy in Platinum-Resistant EOC

Using a high-throughput screen, we have identified that itraconazole, a triazole antifungal drug, enhances the effect of blocking autophagy in cancer cells. Preliminary results obtained in prostate cancer cells from the Wouters-Koritzinsky lab showed a dramatic increase in the accumulation (toxic levels) of free cholesterol in cells treated with itraconazole plus chloroquine. In addition, data showed that other autophagy inhibitors such as pantoprazole sensitize cells to itraconazole, as shown by reduced growth rates. Itraconazole has been reported to alter cholesterol-trafficking, angiogenesis, mTOR and Hedgehog pathways and to have anticancer activity [11, 12, 15-17]. A number of clinical trials have shown that patients with prostate, lung, and basal cell carcinoma have benefited from treatment with itraconazole [15, 18]. In particular, the efficacy of itraconazole in improving prognosis in patients with recurrent or persistent ovarian cancer has been reported [18, 19].
Of relevance, our preliminary screening on 32 ovarian cell lines indicates a significant activity of these two drugs also in this context. This suggests the combination of itraconazole and chloroquine-based drugs may work in other types of cancer.

2.2 Background Therapeutic Information

2.2.1 Itraconazole

Mechanism of action

Itraconazole is a triazole anti-fungal drug used in the prevention and treatment of fungal infections such as aspergillosis, blastomycosis, candidiasis, histoplasmosis, and in some dermatological and nail infections. Its mechanism of action mediated through a decrease of ergosterol synthesis, required for membrane integrity of fungal cells, via inhibition of the lanosterol 14 alpha-demethylase (14DM) catalyst [20, 21].

Clinical Trials in Oncology

Itraconazole has been investigated in multiple tumour types. A phase 2 non-comparative randomized study investigated a low-dose and high-dose schedule of itraconazole monotherapy in men with castration-resistant metastatic prostate cancer [22]. The low dose (200 mg/day) arm closed early after accruing 17 patients because of a pre-specified futility rule. The high dose arm (600 mg/day) was completed, and accrued 29 patients. The PFS at 24 weeks was found to be 11.8% for the low dose arm, and 48% for the high dose arm. The median PFS, a secondary

endpoint, was 11.9 weeks and 35.9 weeks in the two arms, respectively. One patient in the low dose arm and two patients in the high dose arm experienced partial response according RECIST version 1.1 criteria. Toxicity was greater in the high dose arm, with the most common side effects including fatigue, nausea, anorexia, rash, and a syndrome of hypokalaemia, hypertension, and edema. Although there were no grade 4 toxicities, 4 (14%) patients in the high dose arm came off study because of toxicity (one not drug related).

A phase 2 trial of itraconazole added to pemetrexed in non-squamous non-small cell lung cancer (NSCLC) in second line therapy showed that there was some benefit of adding itraconazole to chemotherapy although this was not significant [23]. Patients were randomised to pemetrexed with or without itraconazole at a dose of 200 mg/day; however, the trial had to complete early because of pemetrexed became available in the first line setting, decreasing the intended accrual of 112 patients to 23 (15 in the combination arm and 8 in the pemetrexed alone arm). Primary end points included the PFS rate at three months. At three months, the PFS rate for the combination arm was 67%, versus 29% on the pemetrexed arm (P=0.11). Median PFS was 5.5 months for the combination compared to 2.8 months for pemetrexed (HR=0.399, P=0.089).
A small phase 2 trial in basal cell carcinoma (BCC) investigated single agent itraconazole at doses of 200mg PO BID versus 100mg PO BID [24]. Primary end points were changes in proliferative and Hedgehog-related biomarkers. Secondary end points included change in tumour size for a subset of patients with multiple non-biopsied tumours. Reductions were found in tumour cell proliferation (as measured by Ki67 staining) of 45% (P=0.04), Hedgehog pathway activity (as measured by GLI1 mRNA) of 65% (P=0.03), and reduced tumour area of 24% (95% CI, 18.2–30.0%). Of eight patients with multiple non-biopsied tumours, four achieved partial response, and four had stable disease. The regimen was generally tolerable with one patient withdrawing due to grade 2 fatigue, and one patient because of congestive heart failure from previous chemotherapy with Adriamycin.

A retrospective analysis looked at patients with platinum-resistant ovarian cancer treated with itraconazole in addition to chemotherapy as part of regional practices [19]. Eighteen of 55 patients received itraconazole as part of a second or third line protocol with chemotherapy at a dose of 400–600mg, oral solution administered on days -2 to 2 or 3, on a two-week cycle. The response rate was 18% (10 out of 55 patients), with the response rate for itraconazole with chemotherapy being 32% (6 out of 19 patients) and in the chemotherapy alone arm, only 11% (4 out of 36) (P=0.06). The median PFS for itraconazole plus chemotherapy was 103 days (95% CI > 84 days) and 53 days for the chemotherapy alone arm (95% CI = 38–88 days), (P = 0.014).

Itraconazole pharmacokinetics

Absorption
Itraconazole is rapidly absorbed after oral administration and peak plasma concentrations of the unchanged drug are reached within 2 to 6 hours following an oral dose [25].

Distribution
Itraconazole is highly lipophilic and in plasma it is bound to protein (99.8%) with albumin being the main binding component (99.6% for the hydroxy-metabolite) [25]. It has also a marked

affinity for lipids. Itraconazole is distributed in a large apparent volume in the body (> 700 L), suggesting its extensive distribution into tissues: concentrations in lung, kidney, liver, bone, stomach, spleen and muscle were found to be two to three times higher than corresponding concentrations in plasma. Brain to plasma ratios were about 1 as measured in beagle dogs [26]. The uptake into keratinous tissues, skin in particular, is up to four times higher than in plasma.

Itraconazole is reported to be keratinophilic, tending to accumulate in skin, hair and nails, where it may persist at therapeutic levels long after completion of treatment (stratum corneum: 2-4 weeks, nails: 6-9 months) [27]. Delivery to skin occurs mainly via the sebum, small amounts are excreted in sweat and incorporated into the basal layer of the epidermis by passive diffusion from the blood. The drug reaches the nails through the nail matrix and the nail bed.

Clinical trials measuring itraconazole in vaginal tissue, skin and nails have shown that adequate tissue levels (0.11 g/ml) are maintained up to 3 days, 2 weeks and 6 months after therapy is stopped for vaginal candidiasis, dermatomycosis and onychomycosis, respectively [28].

Metabolism
Since itraconazole is extensively metabolised by the liver, drugs that induce cytochrome P450 may accelerate its elimination and thus lower itraconazole plasma concentrations [29]. Concurrent use of potentially hepatotoxic drugs with itraconazole should either be avoided or closely monitored.

One of the main metabolites of itraconazole is hydroxy-itraconazole, which has in vitro antifungal activity comparable to itraconazole. Plasma concentrations of hydroxy-itraconazole are reported to be about twice those of itraconazole. In vitro studies indicate CYP 3A4 is the major enzyme that is involved in the metabolism of itraconazole [20].

Elimination
Itraconazole is excreted as inactive metabolites to about 35% in urine within 1 week and to about 54% with faeces. Renal excretion of the parent drug accounts for less than 0.03% of the dose, whereas faecal excretion of unchanged drug varies between 3 – 18% of the dose. Itraconazole clearance decreases at higher doses due to saturable hepatic metabolism [30].

2.2.2 Hydroxychloroquine

Mechanism of Action

Hydroxychloroquine interferes with digestive vacuole function within sensitive malarial parasites by increasing the pH. It is hypothesized to inhibit autophagy by acting on the autophagolysosome [7]. This has been demonstrated in a phase I trial of hydroxychloroquine and temsirolimus in patients with advanced solid tumors and melanoma. In this study, hydroxychloroquine was administered in incremental levels with a fixed dose of temsirolimus and an increase in autophagic vacuoles was demonstrated with electron microscopy in peripheral blood mononuclear cells [31].

Clinical Trials in Oncology
Clinical trials have been conducted to investigate the efficacy and safety of chloroquine and its derivatives in combination with traditional chemotherapy. In a study of chloroquine in adjuvant therapy for treatment of glioblastoma multiforme, the mean survival time of patients receiving chloroquine was significantly longer than patients treated with conventional therapy [32].

Safety issues have been raised when combining hydroxychloroquine with chemotherapy. A phase I study using high-dose hydroxychloroquine and dose-intense temozolomide to treat advanced solid malignancies was considered to be safe and tolerable [31]. A similar conclusion has been drawn in a study which combined temsirolimus and hydroxychloroquine for solid malignancies. The 1200 mg/day hydroxychloroquine with temsirolimus was demonstrated to be safe and effective in the clinic [33]. However, another study using hydroxychloroquine in combination with radiation therapy and temozolomide for newly diagnosed glioblastoma indicated that hydroxychloroquine 600 mg/day was the maximum tolerated dose, but autophagy was not achieved consistently in patients and overall survival was not significantly improved [34].

Pharmcokinetics of Hydroxychloroquine

Absorption of hydroxychloroquine has been found to be incomplete and variable (~70% [range: 25 to 100%]) [35]. Hydroxychloroquine is bound to protein in the blood with approximately 40% bound to primarily albumin. The half-life of elimination is approximately 40 days and is primarily metabolized in the liver by cytochrome CYP 2D6 [36]. Hepatic metabolites include bidesethylchloroquine, desethylhydroxychloroquine, and desethylchloroquine. Approximately 15% to 25% is excreted in the urine.

2.2.3 Combination Therapy of Itraconazole and Hydroxychloroquine

Itraconazole and hydroxychloroquine have already been studied extensively (independently of each other) in early phase clinical trials and tolerable doses either as single agents or in combination with other therapies have been established. By extrapolating from the phase 2 prostate cancer study by Antonarakis et al, the dose of itraconazole will be fixed at 600mg daily [22]. Given that there is no putative interaction based on metabolism and pharmacokinetics, a rapid dose escalation of hydroxychloroquine will be attempted.

2.3 Correlative Studies Background

2.3.1 Elucidating the Pharmacodynamic Effect of Hydroxychloroquine and Itraconazole Combination on Tumour Cellular Metabolism: Impairment of cholesterol-trafficking.

Given that the cytotoxic effect of the combination therapy is thought to be mediated via the impairment of cholesterol-trafficking, we will focus on examining genes related to lipoprotein signaling and cholesterol transport, synthesis and metabolism. Tumour and blood samples (archival, pre- and on-treatment) will be obtained from women with advanced EOC.

To determine the biologic mechanisms underlying hydroxychloroquine and itraconazole, tumour biopsies obtained prior to and during treatment will be examined by FILIPIN staining. This will assist in determining accumulation of free cholesterol. Immunofluorescence (IF) assays will be employed to examine co-localization of free cholesterol with late endosomes/ lysosomes (LAMP1). IHC will also be employed to assess the presence of other related proteins including but not limited to NPC1, SCP2 and STARD3. NPC1 is a protein that is found to be aberrant in Neimann-Pick Type C disease which affects cholesterol trafficking. Recently, this same protein has been found to be inhibited by itraconazole causing a block in the cholesterol trafficking pathway and subsequent inhibition of mTOR [37]. SCP2 and STARD3 are thought to be two of the major proteins associated with cellular cholesterol trafficking. SCP2 is responsible for binding cytoplasmic cholesterol and transporting it to the plasma membrane. STARD3 is a transmembrane protein that is responsible for cholesterol efflux [13]. Free and esterified cholesterol will be assessed in pre- and on-treatment tumour tissue by Bodipy staining.

Whole mRNA sequencing will be done on RNA extracted from tumour tissue to determine whether a modification in certain transcripts affects the synthesis or uptake of cholesterol has occurred as a result of hydroxychloroquine and itraconazole.

In tandem, pre- and on-treatment blood will be analyzed colorimetric/flow cytometry to characterize/quantify free and esterified cholesterol accumulation.

We will examine the consequence of impairments in cholesterol trafficking in normal cells. Serum lipid levels will be monitored; and peripheral blood mononuclear cells will be evaluated for lipid accumulation.

2.3.2 Evaluate the Impact of Hydroxychloroquine and Itraconazole on Autophagy

Hydroxychloroquine has previously been shown to inhibit autophagy whereas itraconazole has been shown to do the opposite. Our pre-clinical studies have shown that hydroxychloroquine and itraconazole work synergistically to trap cholesterol in autophagolysosomes, thereby inducing the accumulation of toxic levels of cholesterol within the cell and causing cellular death.

To investigate the role of autophagy in this process, LC3B and p62, both proteins involved in autophagy will be investigated using IHC. Presence of both these proteins indicates a basal autophagy level and inhibition of autophagy will lead to decreased detection [38].

2.3.3 Evaluate Impact of Hydroxychloroquine and Itraconazole Combination Therapy on Proliferation and Apoptosis

To determine the extent to which autophagy contributes to the survival of EOC cells, and conversely, the extent to which inhibition of autophagy contributes to cell death, we will investigate dynamic changes in markers of cell proliferation or death. We examine this using IHC markers of apoptosis (e.g., caspase 3 cleavage products) and proliferation (e.g., ki67).

Gamma-H2AX, a marker of DNA damage, will also be assessed via IHC.

2.3.4 Evaluate Impact of hydroxychloroquine and Itraconazole Upon Angiogenesis in Platinum-resistant EOC

Angiogenesis seems to play an important role in the dissemination of EOC and anti-angiogenic agents such as bevacizumab have been shown to increase response rates to chemotherapy as well as prolong PFS and OS in a select patient population [39, 40]. Interestingly, the downregulation of autophagy has also been described to increase angiogenesis [41]. The interplay between anti-autophagy agents such as hydroxychloroquine and angiogenesis remain largely uninvestigated. However, itraconazole is thought to downregulate angiogenesis [6, 11, 15, 42], and thus, the combination of these specific agents may offer a solution to the escape mechanisms cancer cells may use to circumvent inhibition of autophagy. To investigate the impact of hydroxychloroquine and itraconazole therapy on angiogenesis, IHC will be performed on tumour tissue for CD31 as a marker of endothelial cells as well as HIF-1α, which is induced by hypoxia and mediates VEGF activity and angiogenesis [43, 44]. Investigating the impact of this therapy on angiogenesis will help to further characterize the mechanism of action of the combination treatment.

2.3.5 Determine the Impact of Alterations in Genes and Gene Products Related to Cell Proliferation and Autophagy in Platinum-resistant EOC and Response to Combination hydroxychloroquine and Itraconazole Therapy

Canonical pathways such as PI3K-AKT and mTOR are often altered in cancer and contribute to resistance to therapy. These pathways serve to enhance nutrient uptake and macromolecule biosynthesis and support cellular proliferation. These have also been described to negatively regulate autophagy [45]. We will use next generation sequencing to study the impact of mutations in genes involved with cell metabolism and proliferation thePI3K -AKTand mTOR pathways.

2.3.6 Validation of Putative Biomarker Genes

Patient tissue will be used to create cell lines and investigate the effect of knocking out putative biomarker genes essential to autophagy and cholesterol trafficking using CRSPR/Cas 9 drop out screens. These screens will be used to prospectively validate these genes as important players in the mechanism of action of the combination therapy of hydroxychloroquine and itraconazole.

2.4 Impact on Cancer Care

Should the combination of hydroxychloroquine and itraconazole be found to be a safe, tolerable and demonstrate anti-cancer activity in vivo, this allows for a novel and cost-effective strategy for the treatment of chemotherapy-resistant EOC as well as potentially other chemotherapy-resistant cancers.

3. PATIENT SELECTION

3.1 Inclusion Criteria

3.1.1 Patients aged 18 years or older with histologically or cytologically confirmed epithelial ovarian cancer.

3.1.2 Platinum-resistant or refractory disease defined as a radiological or clinical progression less than six months after having finished platinum-based chemotherapy.

3.1.3 ECOG performance status equal to or less than 1.

3.1.4 Patients must have clinically or radiographically documented measurable disease. Radiographic measurable disease is defined as at least one site of disease that is unidimensionally measurable as follows [46]:

- CT-scan, physical exam ≥10 mm
- Chest X-ray ≥20 mm
- Lymph node short axis ≥15 mm

- All radiology studies must be performed within 28 days prior to registration (35 days if negative).

3.1.5 All systemic therapy must have been completed ≥ 4 weeks prior to enrollment with radiologic evidence of disease progression.

3.1.6 Life expectancy should be more than 3 months.

3.1.7 Patients receiving any medications or substances that are inhibitors or inducers of CYP3A4 are ineligible. Because the lists of these agents are constantly changing, it is important to regularly consult a frequently-updated list such as http://medicine.iupui.edu/clinpharm/ddis/table.aspx; medical reference texts such as the Physicians' Desk Reference may also provide this information.

3.1.8 Laboratory Requirements - within 7 days prior to enrollment:

Haematology:	hemoglobin	≥90 g/L
	absolute neutrophils	≥1.5 × 10^9/L
	platelets	≥100 × 10^9/L
Biochemistry:	bilirubin	within normal limits
	AST(SGOT) /ALT(SGPT)	≤2.5 × institutional upper limit of normal
	serum creatinine	within normal limits
	or	
	creatinine clearance	≥60 mL/min/1.73 m^2 for patients with creatinine levels

above institutional normal

3.1.9 Subjects with treated and asymptomatic brain metastases are eligible. Patients that received palliative radiation (for brain metastases) are eligible if they have been asymptomatic for at least 2 weeks with use of maintenance steroid therapy, and last received radiation at least 4 weeks prior to start of therapy.

3.1.10 Patients must have the ability to understand and willing to sign a written informed consent document.

3.2 Exclusion Criteria

3.2.1 Patients who have not recovered (≤ grade 1) from adverse events related to previous treatments are excluded with the exception of alopecia and lymphopenia. Peripheral sensory neuropathy must be at grade 2 or less.

3.2.2 Patients with any other prior malignancy from which the patient has been disease free for less than 3 years, with the exception of adequately treated and cured basal or squamous cell skin cancer, superficial bladder cancer, carcinoma in situ of any site or any other cancer.

3.2.3 Patients with a history of allergic reactions attributed to compounds of similar chemical or biologic composition to itraconazole or hydroxychloroquine.

3.2.4 Patients with a known G6PD deficiency due to the risk of hemolytic anemia with the use of hydroxychloroquine.

3.2.5 Patients with known retinopathy due to the risk of worsening retinopathy with hydroxychloroquine.

3.2.6 Uncontrolled intercurrent illness including, but not limited to, ongoing or active infection, symptomatic or asymptomatic congestive heart failure, unstable angina pectoris, cardiac arrhythmia, or psychiatric illness/social situations that would limit compliance with study requirements. Patients with previous history of cardiac failure are excluded.

3.2.7 Patients on treatment with statin that cannot be interrupted for the duration of the study.

3.2.8 Patients with chronic Hepatitis B or hepatitis C infections should be excluded because of potential effects on hepatic function and/ or drug interactions.

3.2.9 Patients with known Human Immunodeficiency Virus (HIV) infection.

3.2.10 Patients with intestinal malabsorption or active bowel obstruction.

3.2.11 Patients who already have a clinical indication for treatment with itraconazole (e.g. chronic candidiasis or other fungal infection) or hydroxychloroquine (e.g. lupus).

3.3 Pregnancy

Both itraconazole and hydroxychloroquine are contraindicated in pregnancy. Due to potential teratogenic effects, women of child-bearing potential must agree to use adequate contraception (hormonal or barrier method of birth control; abstinence) prior to study entry and for the duration of study participation. Should a woman become pregnant or suspect she is pregnant while she or her partner is participating in this study, she should inform her treating physician immediately.

3.4 Inclusion of Women and Minorities

Women of all races and ethnic groups are eligible for this trial. This study is designed to include minorities as appropriate. However, the trial is not designed to measure differences in intervention effects. The population of Southern Ontario is ethnically diverse and the proportion of different ethnic groups in the community is provided in the table below. Universal access to health care will ensure that there is no discrimination on the basis of race or gender (Guide to Canadian Human Rights Act: www.chrc-ccdp.ca/public/guidechra.pdf). Individual hospital registries and databases do not routinely collect racial data, under the direction of the Canadian Human Rights Code.

4. REGISTRATION PROCEDURES

4.1 General Guidelines

The Study Coordinator at the Princess Margaret Hospital Consortium Central Office will enter eligible patients on study centrally. The required forms (Registration Checklist) will be provided upon site activation.

Following registration, patients should begin protocol treatment within 72 hours. Issues that would cause treatment delays should be discussed with the Principal Investigator (cc the central office study coordinator). If a patient does not receive protocol therapy following registration, the patient’s registration on the study may be cancelled. The Study Coordinator should be notified of cancellations as soon as possible.

4.2 Registration Process

Prior to registering a patient, each institution must have submitted all necessary regulatory documentation to the Drug Development Program Central Office. The registration checklist will only be sent once this has been received.

No patient can receive protocol treatment until registration with the Central Office has taken place. All eligibility criteria must be met at the time of registration. There will be no exceptions. Any questions should be addressed with the Central Office prior to registration.

To register a patient, the following documents are to be completed by the research nurse or data manager and sent / faxed to the Central Office Study Coordinator:

- Signed patient consent form
- Registration Checklist signed by the investigator

To complete the registration process, central office will review the checklist and once eligibility has been confirmed:

- Assign a patient study number
- Assign the patient a dose
- Register the patient on the study
- Fax or e-mail the confirmation worksheet with the patient study number and dose to the participating site

To ensure immediate attention is given to the faxed checklist, each site is advised to also call the study coordinator listed on the front sheet. Patient registration will be accepted between the hours of 9am to 5pm Monday to Friday, excluding Canadian statutory holidays when the central office will be closed.

5. TREATMENT PLAN

5.1 Agent Administration

Treatment will be administered on an outpatient basis. Reported adverse events and potential risks are described in Section 7. Appropriate dose modifications are described in section 6. No investigational or commercial agents or therapies other than those described below may be administered with the intent to treat the patient's malignancy.

The patient will be requested to maintain a medication diary of each dose of medication. The medication diary will be returned to clinic staff at the end of each course.

Regimen Description					
**Agent**	**Premedications / Precautions**	**Dose**	**Route**	**Schedule**	**Cycle Length**
itraconazole	Take with food	300 mg	PO	twice daily	28 days (4 weeks)
hydroxychloroquine	Take with food or milk Antacids should not be taken within 4 hours before or 4 hours after dosing with hydroxychloroquine	** tablet	PO	** once or twice daily	
**Doses as appropriate for assigned dose level.					

5.2 Definition of Dose Limiting Toxicity

Toxicity will be graded using CTCAE version 4.0, with dose limiting toxicities being assessed during the first 28 days of treatment (cycle 1). Any dose limiting toxicity must be a toxicity that is considered related to study drug. Patients is considered evaluable for DLT assessment if < 7 days of full dose (according the specific dose level) treatment have been omitted. If at any day the treatment will not be assumed at full dose, it will be considered as omitted.

Dose limiting toxicity is defined as follows:

<u>Haematologic:</u>

- Absolute neutrophil count (ANC) <0.5 x 10^9/L
- Febrile Neutropenia (ANC <1.0 x 10^9/L, fever ≥38.5^{o}C)
- Platelets <25 x 10^9/L
- Bleeding felt to be due to thrombocytopenia

<u>Non-Haematologic</u>:

- Diarrhea ≥ Grade 3 despite optimal loperamide use.
- Rash ≥ Grade 3 or grade 2 if medically concerning or unacceptable to the patient.
- Other grade ≥3 effects thought to be treatment related.
- Missing >14 days of treatment for toxicity reasons.

<u>Ocular Toxicity</u>

Any occurrence of ocular toxicity that is greater than grade 1, including but not limited to retinopathy is considered to be a dose-limiting toxicity and the patient must discontinue the study medications.

Any Toxicity
Patients experiencing adverse events ≥ grade 2 that do not recover within 2 weeks, despite optimal supportive management and treatment interruption.

Management and dose modifications associated with the above adverse events are outlined in Section 7.

5.3 Rolling Six Design

The rolling six design allows for accrual of 2 to 6 patients concurrently on the same dose level.

The dose of hydroxychloroquine will be escalated in fixed increments according to the dose escalation scheme outlined below.

**Dose level**	**Dose of itraconazole (total daily dose)**	**Dose of HYDROXYCHLOROQUINE (total daily dose)**	**Minimum number of patients**	**Maximum number of patients**
-1	300 mg BID (600 mg total)	200 mg OD (200 mg total)	--	6
1 (starting)	300 mg BID (600 mg total)	200 mg BID (400 mg total)	2	6
2	300 mg BID (600 mg total)	400 mg BID (800 mg total)	2	6
3	300 mg BID (600 mg total)	600 mg BID (1200 mg total)	2	6

5.3.1 Specific Dose Escalation and De-Escalation Rules for this Trial

- Starting dose level is 1.
- The dose level -1 block will be used if and only if dose level 1 is too toxic (i.e. if dose level 1 is above the MTD).

Determination of which dose level to enroll a patient into will be made according to the table below (adapted from [47]). The decision is based on the number of patients currently enrolled on a specific dose level, how many of them experienced DLTs, and how many patients are still pending data on DLTs experienced.

	DLT Data			Enrolling Dose Level*	
# Currently Enrolled	# Patients with DLTs	# of Patients without DLTs	# of Patients w/Data Pending	When n is lower than Trial Maximum	When n is at Trial Maximum
2	0, 1	Any	Any	n	
2	2	0	0	n - 1	
3	0	0, 1, 2	3, 2, 1	n	
3	0	3	0	n + 1	
3	1	0. 1	2, 1	n	
3	1	2	0	n	
3	≥ 2	Any	Any	n - 1	
4	0	0, 1, 2	4, 3, 2	n	n
4	0	3	1	n	n
4	0	4	0	n + 1	n
4	1	0, 1	3, 2	n	n
4	1	2	1	n	n
4	1	3	0	n	n
4	≥ 2	Any	Any	n - 1	n - 1
5	0	0, 1, 2	5, 4, 3	n	n
5	0	3, 4	2, 1	n	n
5	0	5	0	n + 1	n
5	1	0, 1	4, 3	n	n
5	1	2	2	n	n
5	1	3, 4	1, 0	n	n
5	≥ 2	Any	Any	n - 1	n - 1
6	0	0, 1, 2	6, 5, 4	Accrual hold	Accrual hold
6	0	3, 4	3, 2	Accrual hold	Accrual hold
6	0	5, 6	1, 0	n + 1	RP2D
6	1	0, 1	5, 4	Accrual hold	Accrual hold
6	1	2	3	Accrual hold	Accrual hold
6	1	3,4	2, 1	Accrual hold	Accrual hold
6	1	5	0	n + 1	RP2D
6	≥ 2	Any	Any	n - 1	n - 1

Abbreviations: DLT - dose limiting toxicity; RP2D - recommended phase 2 dose

* n is the current dose level of patients enrolled; n + 1 and n - 1 represent dose level escalation and de-escalation, respectively.

5.3.2 Recommended Phase II Dose

The MTD is defined as the dose at which ≥ 2/3 or ≥2/6 patients experience dose limiting toxicity.

The dose one level below the MTD will be considered the RP2D. If the maximum tolerated dose is not reached on the phase I trial, the maximum administered dose will be the RP2D, which will be used for the purposes of the phase 2 cohort.

Up to a total of 6 patients may be treated at the recommended dose to assure information on the safety profile at that dose is complete.

5.4 General Concomitant Medication and Supportive Care Guidelines

Because there is a potential for interaction of itraconazole and hydroxychloroquine with other concomitantly administered drugs through the cytochrome P450 system, the case report form must capture the concurrent use of all other drugs, over-the-counter medications, or alternative therapies. The Principal Investigator should be alerted if the patient is taking any agent known to affect or with the potential to affect selected CYP450 isoenzymes.

The patient may not take any other anti-cancer or investigation therapy while on study. Radiation therapy for palliative reasons may be permitted for palliative measures such a relief from pain or bleeding while the patient is on study, except for brain metastases. Itraconazole will be held for one week prior to radiotherapy. Patients who are found to have brain metastases requiring radiotherapy while on study will be discontinued from study treatment.

For list of prohibited drugs refer to Appendix C

5.4.1 Patients with Diabetes Mellitus

Patients with diagnosis of diabetes mellitus treated with insulin or oral hypoglycemic drugs needs to carefully monitor blood sugar level due to increased risk of hypoglycemia when treated with itraconazole and/or hydroxychloroquine. Patients with diabetes will be required to monitor and record the capillary blood sugar levels at least three times a day. Dose of insulin or oral hypoglycemic drugs will be adjusted accordingly.

5.5 Duration of Therapy

In the absence of treatment delays due to adverse event(s), treatment may continue as long as patient is benefitting or until one of the following criteria applies:

- Disease progression as defined by RECIST 1.1
- Clinical progression
- Women who become pregnant or are breast feeding
- Sexually active subjects who refuse to use medically accepted forms of contraception (e.g. male condom, female condom) during the study and for 30 days following

discontinuation of study treatment
- Intercurrent illness that prevents further administration of treatment
- Unacceptable adverse event(s)
- Significant non-compliance with the protocol schedule in the opinion of the investigator
- Patients requiring a delay in treatment beyond the allowed time frame
- Patients requiring more than 2 dose reductions of hydroxychloroquine or itraconazole
- Patient decides to withdraw from the study
- General or specific changes in the patient's condition render the patient unacceptable for further treatment in the judgment of the investigator
- Termination of the protocol by a regulatory agency or Sponsor-Investigator, or study medication can no longer be provided

5.6 Duration of Follow Up

Patients will be followed for 12 weeks after removal from study or until death, whichever occurs first. Patients removed from study for unacceptable adverse event(s) will be followed every 4 weeks by the clinic team until resolution or stabilization of the adverse event.

		**Follow-Up Period**			
		30-37 Days from the End of Study Drug Administration	Every 4 weeks (after the off study visit), up to 12 weeks	Until resolution or stabilization of treatment related adverse event(s)	RECIST 1.1 every 12 weeks from last scan until PD
**Reason Patients Removed from study**	Objective Disease Progression	X	X	X (FU every 4 weeks)	
	Clinical Progression/ Symptomatic Deterioration	X	X	X (FU every 4 weeks)	
	Adverse Events or clinically significant lab value	X	X	X (weekly FU for 4-weeks, then monthly)	X
	All other patients	X	X	X (FU every 4 weeks)	X

5.7 Criteria for Removal from Study

Patients will be removed from study when any of the criteria listed below applies:
- Patient death
- Follow up complete
- Termination of the study by regulatory authorities
- Termination of the study by the Sponsor-Investigator

The reason for study removal and the date the patient was removed must be documented in the Case Report Form.

6. DOSING DELAYS/DOSE MODIFICATIONS

Doses will be reduced for haematological and other adverse events. Dose adjustments are to be made according to the greatest degree of toxicity. Adverse events will be graded using the NCI Common Terminology Criteria for Adverse Events Version 4.0 (CTCAE).

When a dose is held due to adverse events, the cycle shall continue even if no study medication is received.

The guidelines which follow outline dose adjustments for several of these toxic effects. <u>If a patient experiences several adverse events and there are conflicting recommendations, please use the recommended dose adjustment that reduces the dose to the lowest level.</u>

The major adverse effects of itraconazole and hydroxychloroquine are listed in section 7.

Dose reduction for itrcozanole as specified in Table below:

**Dose Level**	**itraconazole**
1	*300 mg PO BID*
-1	*200 mg PO BID*
-2	*100mg PO BID*

For dose adjustment of hydroxychloroquine, please refer to dosing guidelines from the phase I component of trial in section 5.2.

Note that patients cannot dose reduce below 200mg OD for hydroxychloroquine and 100 mg BID for itraconazole. Any patients who experience adverse events requiring hydroxychloroquine dose reduction below 200 OD mg or itraconazole below 100 mg BID will stop study related therapy.

**<u>Nausea</u>**	**Management/Next Dose for itraconazole**	**Management/Next Dose for hydroxychloroquine**
≤ Grade 1	No change in dose	No change in dose
Grade 2	Hold* until ≤ Grade 1. Resume at same dose level.	Hold* until ≤ Grade 1. Resume at same dose level.
Grade 3	Hold* until < Grade 2. Resume at one dose level lower, if indicated.**	Hold* until < Grade 2. Resume at one dose level lower, if indicated.**
Grade 4	Off protocol therapy	Off protocol therapy
*Patients requiring a delay of >2 weeks should go off protocol therapy.<br>**Patients requiring > two dose reductions should go off protocol therapy.		
Recommended management: Antiemetics and intravenous hydration if required.		

**<u>Vomiting</u>**	**Management/Next Dose for itraconazole**	**Management/Next Dose for hydroxychloroquine**
≤ Grade 1	No change in dose	No change in dose
Grade 2	Hold* until ≤ Grade 1. Resume at same dose level.	Hold* until ≤ Grade 1. Resume at same dose level.
Grade 3	Hold* until < Grade 2. Resume at one dose level lower, if indicated.**	Hold* until < Grade 2. Resume at one dose level lower, if indicated.**
Grade 4	Off protocol therapy	Off protocol therapy
*Patients requiring a delay of >2 weeks should go off protocol therapy.<br>**Patients requiring > two dose reductions should go off protocol therapy.		
Recommended management: Antiemetics and intravenous hydration if required.		

**<u>Diarrhea</u>**	**Management/Next Dose for itraconazole**	**Management/Next Dose for hydroxychloroquine**
≤ Grade 1	No change in dose	No change in dose
Grade 2	Hold* until ≤ Grade 1. Resume at same dose level.	Hold* until ≤ Grade 1. Resume at same dose level.
Grade 3	Hold* until < Grade 2. Resume at one dose level lower, if indicated.**	Hold* until < Grade 2. Resume at one dose level lower, if indicated.**
Grade 4	Off protocol therapy	Off protocol therapy
*Patients requiring a delay of >2 weeks should go off protocol therapy.<br>**Patients requiring > two dose reductions should go off protocol therapy.		
Recommended management: Loperamide antidiarrheal therapy<br>Dosage schedule: 4 mg at first onset, followed by 2 mg with each loose motion until diarrhea-free for 12 hours (maximum dosage: 16 mg/24 hours)<br>Adjunct anti-diarrheal therapy is permitted and should be recorded when used.		

**<u>Neutropenia</u>**	**Management/Next Dose for itraconazole**	**Management/Next Dose for hydroxychloroquine**
≤ Grade 1	No change in dose	No change in dose
Grade 2	Hold* until ≤ Grade 1. Resume at same dose level.	Hold* until ≤ Grade 1. Resume at same dose level.
Grade 3	Hold* until < Grade 2. Resume at one dose level lower, if indicated.**	Hold* until < Grade 2. Resume at one dose level lower, if indicated.**
Grade 4	Off protocol therapy	Off protocol therapy
*Patients requiring a delay of >2 weeks should go off protocol therapy.<br>**Patients requiring > two dose reductions should go off protocol therapy.		

**<u>Anemia</u>**	**Management/Next Dose for itraconazole**	**Management/Next Dose for hydroxychloroquine**
≤ Grade 1	No change in dose	No change in dose
Grade 2	No dose reduction or delay for anemia is required. The investigator can	

<table>
<tr><th><u>Anemia</u></th><th>Management/Next Dose for itraconazole</th><th>Management/Next Dose for hydroxychloroquine</th></tr>
<tr><td>Grade 3</td><td colspan="2" rowspan="2">elect to dose reduce or hold the treatment, if considered clinically indicated. Blood transfusion is allowed at each time during the study (screening, treatment and follow-up).</td></tr>
<tr><td>Grade 4</td></tr>
</table>

<u>Thrombocytopenia</u>	Management/Next Dose for itraconazole	Management/Next Dose for hydroxychloroquine
≤ Grade 1	No change in dose	No change in dose
Grade 2	Hold* until ≤ Grade 1. Resume at same dose level.	Hold* until ≤ Grade 1. Resume at same dose level.
Grade 3	Hold* until < Grade 2. Resume at one dose level lower, if indicated.**	Hold* until < Grade 2. Resume at one dose level lower, if indicated.**
Grade 4	Off protocol therapy	Off protocol therapy

*Patients requiring a delay of >2 weeks should go off protocol therapy.
**Patients requiring > two dose reductions should go off protocol therapy.

<u>Retinopathy</u>	Management/Next Dose for itraconazole	Management/Next Dose for hydroxychloroquine
Grade 1	No change in dose	Hold* until asymptomatic. Resume at one dose level lower.
Grade 2	Off protocol therapy	Off protocol therapy
Grade 3	Off protocol therapy	Off protocol therapy
Grade 4	Off protocol therapy	Off protocol therapy

*Patients requiring a delay of >2 weeks should go off protocol therapy.
Patient should have immediate consultation with an ophthalmologist.

<u>Other non-hematological toxicity</u>	Management/Next Dose for itraconazole	Management/Next Dose for hydroxychloroquine
≤ Grade 1	No change in dose	No change in dose
Grade 2	Hold* until ≤ Grade 1. Resume at same dose level.	Hold* until ≤ Grade 1. Resume at same dose level.
Grade 3	Hold* until < Grade 2. Resume at one dose level lower, if indicated.**	Hold* until < Grade 2. Resume at one dose level lower, if indicated.**
Grade 4	Off protocol therapy	Off protocol therapy

*Patients requiring a delay of >2 weeks should go off protocol therapy.
**Patients requiring > two dose reductions should go off protocol therapy.

This table only applies to non-hematological adverse events considered related to the study medication and considered clinically significant as per investigator judgment. Clinically non-significant, treatable or reversible lab abnormalities including, but not limited to alkaline phosphatase or gamma-glutamyl transferase, uric acid, or electrolytes abnormalities does not require dose modifications.

7. ADVERSE EVENTS

The adverse drug reactions are ranked by frequency, using the following convention:

- Common (≥ 1/100 to < 1/10)
- Uncommon (≥ 1/1,000 to < 1/100)
- Rare (≥ 1/10,000 to < 1/1,000)
- Very rare (< 1/10,000)
- Not known (cannot be estimated from the available data)

7.1 List of Adverse Events and Reporting Requirements

This study will utilize the CTCAE version 4.0 for toxicity and Adverse Event reporting. A copy of the CTCAE version 4.0 can be downloaded from the CTEP home page (http://ctep.cancer.gov). All appropriate treatment areas should have access to a copy of the CTCAE version 4.0.

7.1.1 Expected Adverse Events and Protocol-Specific Expedited Adverse Event Reporting for Investigational Agent(s)

Adverse event (AE) monitoring and reporting is a routine part of every clinical trial. The following list of AEs (Sections 7.1.1.1 and 7.1.1.2) and the characteristics of an observed AE (Section 7.2) will determine whether the event requires expedited reporting as an SAE in addition to routine reporting.

In addition, hospitalizations for routine procedures, protocol treatment, blood sampling, investigations and tissue biopsies are <u>*NOT*</u> *considered SAE in this protocol.*

7.1.1.1 Expected Adverse Events using Itraconazole

**Blood and Lymphatic System Disorders**	Very rare: Leucopenia and neutropenia, thrombocytopenia
**Immune System Disorders**	Very rare: Serum sickness, angioneurotic oedema, anaphylactic, anaphylactoid and allergic reactions
**Metabolism and Nutrition Disorders**	Rare: Hypertriglyceridemia, hypokalaemia
**Nervous System Disorders**	Very rare: Peripheral neuropathy, paraesthesia, hypoaesthesia, headache, dizziness
**Eye Disorders**	Very rare: Visual disturbances, including vision blurred and diplopia
**Ear and Labyrinth Disorder**	Very rare: Tinnitus, transient or permanent hearing loss

**Cardiac Disorders**	Rare: Chest pain<br>Very rare: Congestive heart failure
**Respiratory, Thoracic and Mediastinal Disorders**	Very rare: Pulmonary oedema, dyspnoea
**Gastrointestinal Disorders**	Very rare: Pancreatitis, abdominal pain, vomiting, dyspepsia, nausea, diarrhoea, constipation, dysgeusia
**Hepatobiliary Disorders**	Very rare: Serious hepatotoxicity (including some cases of fatal acute liver failure), hepatitis, reversible increases in hepatic enzymes
**Skin and subcutaneous tissue disorders**	Very rare: Toxic epidermal necrolysis, Stevens-Johnson syndrome, actue generalised exanthematous pustulosis, erythema multiforme, exfoliative dermatitis, leukocytoclastic vasculitis, urticaria, alopecia, photosensitivity, rash, pruritus
**Musculoskeletal and Connective Tissue Disorders**	Very rare: Myalgia, arthralgia
**Renal and Urinary Disorders**	Very rare: Pollakiuria, urinary incontinence
**Reproductive System and Breast Disorders**	Very rare: Menstrual disorders, erectile dysfunction
**General Disorders**	Very rare: Oedema, pyrexia

7.1.1.2 Expected Adverse Events using Hydroxychloroquine

**Blood and lymphatic system disorders**	Bone marrow depression, anemia, aplastic anemia, agranulocytosis, leucopenia, thrombocytopenia.
**Cardiac disorders**	Cardiomyopathy, which may result in cardiac failure and in some cases a fatal outcome. Chronic toxicity should be considered when conduction disorders (bundle branch block/ atrioventricular heart block) as well as biventricular hypertrophy are found. Drug discontinuation may lead to recovery.
**Ear and labyrinth disorders**	Vertigo, tinnitus. Hearing loss including cases of irreversible hearing loss.
**Eye disorders**	Common: Blurring of vision due to a

	disturbance of accommodation which is dose dependent and reversible.<br><br>Uncommon: Maculopathies which may be irreversible. Retinopathy with changes in pigmentation and visual field defects. In its early form it appears reversible upon discontinuation of the drug. If allowed to develop however, there may be a risk of progression even after treatment withdrawal. Patients with retinal changes may be asymptomatic initially, or may have scotomatous vision with paracentral, pericentral ring types, temporal scotomas, abnormal colour visions, reduction in visual acuity, night blindness, difficulty reading and skipping words. Corneal changes including edema and opacities. They are either symptomless or may cause disturbances such as halos around lights especially at night, blurring of vision or photophobia. They may be transient or are reversible upon discontinuation of therapy. Macular degeneration which may be irreversible.
**Gastrointestinal disorders**	Very common: Abdominal pain, nausea<br><br>Common: Diarrhea, vomiting. These symptoms usually resolve immediately upon reducing the dose or upon stopping the treatment.
**Hepatobiliary disorders**	Uncommon: Abnormal liver function tests, fulminant hepatic failure.
**Immune system disorders**	Urticaria, angioedema, bronchospasm.
**Metabolism and nutrition disorders**	Common: Anorexia (usually resolves immediately upon reducing

	the dose or upon stopping the treatment). Hypoglycemia. May exacerbate porphyria.
**Musculoskeletal and connective tissue**	Uncommon: Sensory motor disorders.<br><br>Not known: Skeletal muscle palsies or skeletal muscle myopathy. Depression of tendon reflexes, abnormal results of nerve conduction tests. Myopathy may be reversible after drug discontinuation, but recovery may take many months.
**Nervous system disorders**	Common: Headache<br><br>Uncommon: Dizziness.<br><br>Not known: Convulsions. Extrapyramidal reactions such as: akathisia, dystonia, dyskinesia, gait disturbance, tremor.
**Psychiatric disorders**	Common: Affect lability.<br><br>Uncommon: Nervousness Not known: Psychosis, suicidal behavior.
**Skin and subcutaneous tissue disorders**	Common: Skin rash, pruritus Uncommon: Pigmentary changes in skin and mucous membranes, bleaching of hair, alopecia. These usually resolve readily upon cessation of therapy.<br><br>Not known: Bullous eruptions (including urticarial, morbilliform, lichenoid, maculopapular, purpuric, erythema annulare centrifugum), toxic epidermal necrolysis, erythema multiforme, Stevens-Johnson syndrome, Drug Rash with Eosinophilia and Systemic Symptoms (DRESS syndrome), photosensitivity, exfoliative dermatitis, acute generalized exanthematous pustulosis (AGEP).

	AGEP has to be distinguished from psoriasis, although hydroxychlorquine may precipitate attacks of psoriasis. It may be associated with fever and hyperleukocytosis. Outcome is usually favorable after discontinuation of drug.

7.2 Adverse Event Characteristics

- **CTCAE term (AE description) and grade:** The descriptions and grading scales found in the revised NCI Common Terminology Criteria for Adverse Events (CTCAE) version 4.0 will be utilized for AE reporting. All appropriate treatment areas should have access to a copy of the CTCAE version 4.0. A copy of the CTCAE version 4.0 can be downloaded from the CTEP web site http://ctep.cancer.gov/protocolDevelopment/electronic_applications/ctc.htm.

- **For expedited reporting purposes only:**
  - AEs for the agent that do not require expedited reporting are defined in Section 7.1.

- **Attribution** of the AE:
  - Definite – The AE *is clearly related* to the study treatment.
  - Probable – The AE *is likely related* to the study treatment.
  - Possible – The AE *may be related* to the study treatment.
  - Unlikely – The AE *is doubtfully related* to the study treatment.
  - Unrelated – The AE *is clearly NOT related* to the study treatment.

Associated with the use of the *drug/biologic:* There is a reasonable possibility that the experience may have been caused by the drug/biologic.

Life threatening adverse *drug/biologic* experience: Any adverse drug/biologic experience that places the subject, in the view of the investigator, at immediate risk of death from the reaction as it occurred.

Serious adverse *drug/biologic* experience: Any event is an AE occurring at any dose that results in any of the following outcomes:

- Death
- A life-threatening adverse event (The patient was, in the view of the Investigator, at immediate risk of death from the event as it occurred. It does not mean that the event, had it occurred in a more severe form, might have caused death)Inpatient hospitalization or prolongation of existing hospitalization for ≥ 24 hours. If a complication prolongs hospitalization or fulfills any other serious criteria, the event is serious. Hospitalization for elective treatment of a pre-existing condition that did not worsen from baseline is not

considered to be an AE)

- A persistent or significant incapacity or substantial disruption of the ability to conduct normal life functions. This definition is not intended to include experiences of relatively minor medical significance such as uncomplicated headache, nausea, vomiting, diarrhea, influenza, accidental trauma (i.e., sprained ankle) that may interfere or prevent everyday life functions but do not constitute a substantial disruption).
- A congenital anomaly/birth defect
- Important Medical Events (IME) that may not result in death, be life threatening, or require hospitalization may be considered serious when, based upon medical judgment, they may jeopardize the patient or subject and may require medical or surgical intervention to prevent one of the outcomes listed in this definition. (Examples include allergic bronchospasm requiring intensive treatment in an emergency room or at home, blood dyscrasias or convulsions that do not result in hospitalization or the development of drug dependency or drug abuse)

Any SAE occurring after the patient has provided informed consent and until 4 weeks after the patient has stopped study participation must be reported. This includes the phase in which the study protocol interferes with the standard medical treatment given to a patient (e.g. treatment withdrawal during screening phase, change in treatment to a fixed dose of concomitant medication). Serious adverse events occurring more than 4 weeks after study discontinuation need only be reported if a relationship to study drug [is suspected.

Unexpected adverse *drug/biologic* experience: Any adverse drug/biologic experience, the nature, frequency, or severity of which is not consistent with the product monograph, or not consistent with the risk information described above as a protocol-specific expected adverse event (see Section 7.1.1 "Expected Adverse Events and Protocol-Specific Expedited Adverse Event Reporting Exclusions", above).

7.3 Serious Adverse Event Reporting

7.3.1 Sponsor Notification

Any serious adverse event must be reported to the Central Office within 24 hours of the Investigator at the site learning of the event by a completed SAE form. The adverse event must be completely described in the case report form.

The Princess Margaret Cancer Centre DDP Clinical Trials Group will provide expedited reports of on-study SAEs to Health Canada for those events which meet regulatory requirements for expedited reporting, i.e. events which are BOTH serious AND unexpected (as determined by reference to the Investigator Brochure), AND which are thought to be related to protocol treatment (or for which a causal relationship with protocol treatment cannot be ruled out).

All adverse signs and symptoms which occur during or following the course of drug administration must be reported in detail on the subject's case report form. This description is to include the nature of the sign or symptom, time of onset in relation to drug application, duration, severity, and

possible relationship to drug, required therapy, and outcome. The subject should be followed until the adverse reaction is resolved, or until in the opinion of the Principal Investigator, reversal of the reaction is not likely to occur.

7.3.2 SAE Follow-up

Follow-up SAE reports is subject to the same timelines as the initial report, and is sent to the same parties to whom the original Serious Adverse Event Form was sent. A new serious adverse event form is completed for the follow-up, stating that this is a follow-up to the previously reported serious adverse event and giving the date of the original report. Each re-occurrence, complication or progression of the original event should be reported as a follow-up to that event. The follow-up information should describe whether the event has resolved or continues, if and how it was treated, and whether the patient continued or discontinued study participation.

7.3.3 REB Notification of SAEs

Investigators must notify their Research Ethics Boards (according to their local REB policies) and file the report in their study files. Documentation as outlined below must be maintained for reportable SAEs. Documentation that serious adverse events (SAEs) have been reported to REB must be forwarded to the DDP and kept on file at the Centre. Documentation can be any of the following:

- letter or email from the REB acknowledging receipt
- stamp from the REB, signed and dated by REB chair, acknowledging receipt
- letter or email demonstrating the SAE was sent to the REB

7.3.4 Health Canada SAE Reporting

All serious, unexpected adverse drug reactions must also be reported by the Central Office to Health Canada within 15 days if the reaction is neither fatal nor life threatening, and within 7 days if the reaction is fatal or life threatening.

As the sponsor of this study in Canada, the Principal Investigator for this study will be responsible for reporting all serious unexpected adverse events to Health Canada.

7.4 Routine Adverse Event Reporting

For abnormal laboratory values, it is the responsibility of the Principal Investigator to assess the clinical significance of each abnormality. Only abnormal laboratory values that can be assessed for grade using CTCAE version 4 will be documented. The Investigator will determine whether abnormal laboratory values are considered clinically significant and represent AEs based on their medical judgment.

Data on all adverse experiences/toxicities regardless of seriousness or attribution must be collected in source and CRFs for documentation purposes only.

Clinical Laboratory Abnormalities:

All abnormal and clinically significant laboratory values should be captured on source documentation and assessed for clinical significance by the Investigator at the site. This assessment of clinical significance is to be documented in physician dictation. Only abnormal laboratory values deemed clinically significant and are gradeable per CTCAE version 4.0 should be listed as AEs in source and the CRFs. All clinically significant abnormal laboratory results will be followed up until the related AE resolves, returns to < grade 1 or baseline value in the follow-up period. Additionally, laboratory abnormalities resulting in an intervention or dose modification are considered to be clinically significant.

7.5 Documentation of Adverse Events

All AEs must be captured in the source documents, as well as reported in *electronic document capture (EDC) system*. AEs reported using SAE forms must also be reported in *EDC system*.

All serious and non-serious AEs occurring from the start of study medication administration to the end of study drug administration visit must be recorded as AEs on the CRF. Investigators, sub-investigators, and delegated individuals should review all documentation (e.g., hospital progress notes, laboratory, or diagnostic reports) relative to the event being reported.

7.6 Follow-Up of AEs and SAEs

SAEs and AEs should be followed for 12 weeks after the last dosing of study drug/biologic or until they are resolved (return to normal or baseline values), stabilized, improve to Grade 1, or the patient is lost to follow-up and cannot be contacted. Additional investigations (*e.g.*, laboratory tests, diagnostic procedures, or consultation with other healthcare professionals) may be required to completely investigate the nature and/or causality of an AE or SAE. If the patient dies during the study or within 12 weeks following the last dose of study medication, any postmortem findings (including histopathology) should be provided to the Sponsor. CRF data should be updated with any new information as appropriate.

7.7 Pregnancy

Any pregnancy of a study subject or of a study subject's partner that occurs during study participation should be reported for pregnancies that occur up to 30 days after the last dose of study medication. If the study subject is the father, then two SAE forms should be completed, one for the father and the other for the mother and neonate. The pregnancy should be followed up to determine outcome, including spontaneous or voluntary termination, details of birth, and the presence or absence of any birth defects, congenital abnormalities or maternal and newborn complications.

7.8 Investigator Notifications

As both drugs are available as commercial products, the Sponsor-Investigator will not be receiving any Investigator Notifications throughout the lifetime of this study.

7.9 Data Safety and Monitoring Board

A Data Safety and Monitoring Board, an independent group of experts, will be reviewing the data from this research throughout the study to see if there are unexpected or more serious side effects than described in the consent. For this trial, the Drug Development Program DSMB will be responsible for these tasks.

8. PHARMACEUTICAL INFORMATION

8.1 Investigational Agents

8.1.1 Itraconazole

Intraconazole Manufacturing

Janssen Inc. manufactures itraconazole for clinical use.

Intraconazole Packaging and Labeling

Itraconazole will be packaged as per commercial standards. Labels will be affixed to the packaging containing the following information in English and French:

Protocol Code:

Investigational Drug name and formulation (mg)

Lot Number:

Contents: Number of capsules (XX capsules)

Store at XX Temperature (XX °C)

Investigational drug. To be used by qualified investigators only.

Sponsor-Investigator: Dr. Stephanie Lheureux

Investigational agent Handling and Storage

Investigational agent will be stored under secure (with limited access), and temperature-controlled conditions for the duration of the study. Investigational agent bottles will be stored at ambient temperature (i.e.15-30°C) in an area accessible only to authorized staff. Patients will be asked to store their one-month supply of drug at ambient temperature. Study drug inventory forms will be kept by the Investigator, or designee.

Intraconazole Ordering and Shipping

Itraconazole will be purchased by each site from commercial supply.

Study medication should only be dispensed once a patient has (1) signed an informed consent form (ICF), (2) met all eligibility criteria for entry into the study, (3) completed all screening and continuing eligibility requirements, and (4) been assigned a patient identification number.

Agent Accountability

The Investigator is responsible for study medication accountability, reconciliation, and record maintenance. In accordance with all applicable regulatory requirements, the Investigator or designated study personnel must maintain study medication accountability records throughout the study.

The accountability records maintained during the study will be used to support patient dosing data. Site personnel are responsible for reconciling and resolving discrepancies in study medication accountability.

Missing study medication must be recorded along with an explanation of the discrepancy.

Agent Inventory Records

The investigator, or a responsible party designated by the investigator, must maintain a careful record of the inventory and disposition of all agents received using their site-specific Drug Accountability Record Form (DARF).

Description: Itraconazole is a synthetic triazole antifungal agent.

Physical Characteristics: A white or almost white powder, practically insoluble in water, freely soluble in methylene chloride, sparingly soluble in tetrahydrofuran, very slightly soluble in alcohol.

Product description*:* Itraconazole is available as capsules containing 100 mg of itraconazole, with a blue opaque cap and pink transparent body. They are supplied in unit-dose blister packs or HDPE bottles.

Storage requirements: This medicinal product does not require any special temperature storage conditions. Store in the original package in order to protect from light and moisture.
Shelf life: 30 months.

Route of administration: Oral administration and should be taken with food. Capsules should be swallowed whole.

8.1.2 Hydroxychloroquine

Hydroxychloroquine Manufacturing

Apotex Inc. manufactures hydroxychloroquine for clinical use.

Hydroxychloroquine Packaging and Labeling

Hydroxychloroquine will be packaged as per commercial standards. Labels will be affixed to the packaging. Study drug labeling will contain the following information in English and French:

Protocol Code:

Investigational Drug name and formulation (mg)

Lot Number:

Contents: Number of tablets (XX tablets)

Store at XX Temperature (XX °C)

Investigational drug. To be used by qualified investigators only.

Sponsor-Investigator: Dr. Stephanie Lheureux

Hydroxychloroquine Handling and Storage

Investigational agent will be stored under secure (with limited access), and temperature-controlled conditions for the duration of the study. Investigational agent bottles will be stored at ambient temperature (i.e.15-25°C) in an area accessible only to authorized staff. Patients will be asked to store their one-month supply of drug at ambient temperature. Study drug inventory forms will be kept by the Investigator, or designee.

Hydroxychloroquine Ordering and Shipping

Hydroxychloroquine will be purchased by each study site from commercial supply., and shipped directly to each site.

Study medication should only be dispensed once a patient has (1) signed an informed consent form (ICF), (2) met all eligibility criteria for entry into the study, (3) completed all screening and continuing eligibility requirements, and (4) been assigned a patient identification number.

Agent Accountability
The Investigator is responsible for study medication accountability, reconciliation, and record maintenance. In accordance with all applicable regulatory requirements, the Investigator or designated study personnel must maintain study medication accountability records throughout the study.

The accountability records maintained during the study will be used to support patient dosing data. Site personnel are responsible for reconciling and resolving discrepancies in study medication accountability.

Missing study medication must be recorded along with an explanation of the discrepancy.

Agent Inventory Records
The investigator, or a responsible party designated by the investigator, must maintain a careful record of the inventory and disposition of all agents received using their site-specific Drug Accountability Record Form (DARF).

Description: Hydroxychloroquine is an antimalarial drug derived from chloroquine.

Product description:. Each white, capsule-shaped, biconvex film-coated tablet engraved ‘APO’ on one side and ‘HCQ 200’ on the other contains hydroxychloroquine sulfate 200 mg. Available in bottles of 100 and 500 tablets, unit dose packages of 30 and 100.

Storage requirements: Store at room temperature (15°C -30°C).

Route of administration: Oral administration should be taken with food or milk. Do not crush pills.

9. CORRELATIVE, AND SPECIAL STUDIES

9.1 Laboratory Correlative Studies

Clinical response to treatment as defined by the main drug treatment clinical protocol will be compiled and results of biomarker analysis from “responders” and “non-responders” will be compared. These correlative studies are exploratory in nature and will be used in the design of future larger studies.

Laboratories from the following institutions will be involved in correlatives analysis:

- Princess Margaret Cancer Centre Labs, Toronto, Ontario;
- University Health Network Lab, Toronto, Ontario;
- Ontario Institute for Cancer Research (OICR), Toronto, Ontario;
- Princess Margaret, OICR Translational Genomic Labs, Toronto, Ontario.

9.1.1 Tumour Biopsy Assessment

Pre-treatment biopsies will be performed up to 28 days prior to the start of treatment and on-treatment biopsies will be obtained between days 8 and 14 of cycle 1 of treatment.

Immunofluorescence (IF) assays will be employed to examine co-localization of free cholesterol (FILIPIN stain) with late endosomes/ lysosomes (LAMP1 stain). IHC will also be employed to

assess the presence of NPC1, SCP2 and STARD3 as markers of cholesterol trafficking. Bodipy lipid staining will be performed to detect levels of free and esterified cholesterol.

RNA will be extracted for a custom PCR array of genes involved in lipoprotein signaling, cholesterol transport, synthesis and metabolism, and autophagy.

IHC LC3B, p62, caspase 3 cleavage products, gamma-H2AX and Ki67 as markers of autophagy (LC3B/p62), apoptosis (caspase 3 cleavage products and gamma-H2AX) and proliferation (Ki67). IHC for CD31 and HIF-1α will be done to investigate the role of angiogenesis.

Next generation sequencing for *mTOR, AKT-PI3K* pathway genes will be performed to investigate their role in resistance and authophagy.

If there are is any tissue remaining after the above mentioned investigations are completed, it will be banked for future use to validate and investigate as yet unknown biomarker targets or mechanisms of action.

9.1.1.1 Opportunistic tissue collection

Patients undergoing a biopsy or drainage of pleural fluid or ascites for diagnostic or therapeutic purposes may have a sample stored for future research on as yet unknown biomarkers or mechanisms of action. This collection is optional.

9.1.1.2 Collection of Specimens

Archival slides and tumor biopsies will be obtained. Refer to the study-specific Laboratory Manual for detailed collection and processing procedures.

9.1.1.3 Handling of Specimens

Refer to the study-specific Laboratory Manual for detailed collection and processing procedures.

9.1.1.4 Shipping of Specimens

Correlative Studies Program
Princess Margaret Cancer Centre
610 University Avenue 7- 420
Toronto, Ontario M5G 2M9
Tel: (416) 946-4501 ext 5047
Fax: (416) 946-4431
Email: CCRUcorrelativestudies@uhn.ca

9.1.1.5 Site Performing Correlative Study

Princess Margaret Cancer Centre will be the site performing correlative studies for archival tissue.

9.1.2 Peripheral Blood Assessment

Peripheral blood cells, plasma, and serum will be isolated from phlebotomy specimens. These samples will be used fresh or stored for future analysis. Blood will be analyzed colorimetric/flow cytometry to characterize/quantify free and esterified cholesterol accumulation.

If there are is any tissue remaining after the above mentioned investigations are completed, it will be banked for future use to validate and investigate as yet unknown biomarker targets or mechanisms of action.

9.1.2.1 Shipping of Specimens

Heparinized blood must be kept at room temperature after collection. Blood for sera should be kept upright at room temperature and clot for 30-60 minutes. Centrifuge blood at 1500g for 15 minutes. Divide serum evenly into two aliquots. Freeze samples at -70°C and store until shipping.

Refer to the study-specific Laboratory Manual for detailed collection and processing procedures.

9.1.2.2 Handling of Specimens

Blood specimens will be sent to the Correlative Studies Program (PM Cancer Centre):
Correlative Studies Program
Princess Margaret Cancer Centre
610 University Avenue 7-420
Toronto, Ontario M5G 2M9
Tel: (416) 946-4501 ext 5047
Fax: (416) 946-4431
Email: CCRUcorrelativestudies@uhn.ca

Specimens must be shipped Monday through **Wednesday**. For any samples collected on a Friday, the samples should be **processed on site**, and shipped out the following Monday. If sites are unable to process the PBMC locally, they MUST ship the sample Mon-Wed to the Correlative Studies Program. PBMC samples should not be collected on a Thursday or Friday unless the site is able to complete the processing.

9.1.2.3 Site Performing Correlative Study

Princess Margaret Cancer Centre will be the site performing correlative studies for peripheral blood.

9.1.2.4 Collection of Specimens

Bloods include 3 x 10 ml tubes for heparinized blood, and one 5 ml tubes for sera (SST tube). Blood will be obtained at the following study visits: Pre-study, Day 1 and 8 on study.

10. STUDY CALENDAR

Baseline (pre-study) evaluations are to be conducted within 28 days prior to start of protocol therapy, unless specified differently in the study calendar. Scans and x-rays must be done within 28 days prior to the start of therapy. In the event that the patient's condition is deteriorating, laboratory evaluations should be repeated within 48 hours prior to initiation of the next cycle of therapy. The following schedule of assessments applies to all subjects. More frequent assessments should be obtained if clinically indicated. For subsequent cycles 2+, day 1 assessments can be completed up to 2 days prior to day 1. A cycle is 28 days long for the purposes of this protocol.

	Pre-Study (within 28 days)	Pre study (within 10 days)	Cycle 1				Cycle 2+				Off Treatment (30-37days after last dose)[c] + Follow-Up[f]
			D 1	D 8 (+/- 2 days)	D 15 (+/- 2 days)	D 22 (+/- 2 days)	D 1 (+/- 2 days)	D 8 (+/- 2 days)	D 15 (+/- 2 days)	D 22 (+/- 2 days)	
Itraconazole			A---A								
Hydroxychloroquine			B--B								
Informed consent	X										
Demographics	X										
Medical history	X										
Concurrent meds	X		X---X								
Physical exam	X		X	X	X	X	X				X
Vital signs (Blood pressure, pulse, respiratory rate, temperature)	X		X	X	X	X	X				X
Height	X										
Weight	X		X				X				X
Performance status	X		X	X	X	X	X				X
CBC w/differential	X	X	X	X	X	X	X				X
Serum chemistry[a]	X	X	X	X	X	X	X				X
Ca 125	X		X				X				
EKG	X		X				X				
MUGA or echocardiogram	X										
Eye Assessment[j]	X		to be performed every 2 cycles								
Adverse event evaluation			X--X								X
Tumor measurements	X		Tumor measurements are done every 8 weeks post-initiation of treatment +/- 7 days. Documentation (radiologic) must be								X[g]

			provided for patients removed from study for progressive disease.								
Radiologic evaluation	X		Radiologic measurements should be performed every 8_weeks, regardless of any delay in treatment. Documentation must be provided for patients removed from study for progressive disease.								X[g]
B-HCG	X[b]										
Tumour biopsy	X[d]			X[d]							
PBMC & Plasma[h]	X[i]			X							
Serum[h]	X[i]		X	X							
Opportunistic tissue/fluid collection [e]			X---X								

A: Itraconazole: Dose as assigned; twice daily for 28 days
B: Hydroxychloroquine: Dose as assigned; twice daily for 28 days
a: Albumin, alkaline phosphatase, total bilirubin, bicarbonate, BUN, calcium, chloride, creatinine, glucose, LDH, magnesium, phosphorus, potassium, total protein, SGOT [AST], SGPT [ALT], sodium LDL, HDL, total cholesterol, Apolipoprotein B and triglycerides.
b: Serum pregnancy test (women of childbearing potential).
c: Off-study evaluation and follow up as described in section 5.5
d: Pre-treatment biopsy to be done up to 28 days prior to start of therapy. On-treatment biopsy to be done on day 8-14.
e: Patients undergoing a biopsy or fluid collection for diagnostic or therapeutic reasons may have a sample of their tissue or fluid collected for the purposes of this study.
f: Patients will be followed every 4 weeks from the End of Treatment visit for 12 weeks or until resolution of adverse events for patients who will come off study due to toxicity.
g: Only patients who come off study treatment for reasons other than progressive disease (radiological or clinical) need to have scans and RECIST measurements performed. This will occur every 12 weeks from the previous scan until documented progression (radiological or clinical).
h: Three 10 mL tubes of heparinized blood will be collected for PBMC and plasma isolation, and one 5 mL SST tube for sera.
i: Samples must be drawn prior to MUGA.
j. The following eye tests will be performed: Ocular Symptom Assessment, Visual Acuity, Slit Lamp Examination, Intraocular Pressure, Dilated Fundoscopic Examination, Schirmer test and any other diagnostic test.

11. MEASUREMENT OF EFFECT

11.1 Antitumor Effect – Solid Tumors

Patients with measurable disease will be assessed by standard criteria. For the purposes of this study, patients should be re-evaluated for response every 8 weeks. In addition to a baseline scan, confirmatory scans should also be obtained at least 4 weeks following initial documentation of objective response.

In the expansion cohort, tumours will be re-evaluated every 8 weeks during treatment, and at least 4 weeks after the first observation of a complete or partial response. After discontinuation of protocol treatment, patients who have discontinued treatment for reasons other than disease progression will still be re-evaluated every 12 weeks.

Response and progression will be evaluated in this study using the new international criteria proposed by the revised Response Evaluation Criteria in Solid Tumors (RECIST) guideline (version 1.1) [*Eur J Ca* 45:228-247, 2009]. The published RECIST document is available at http://www.eortc.be/RECIST. Changes in the largest diameter (unidimensional measurement) of the tumor lesions and the shortest diameter in the case of malignant lymph nodes are used in the RECIST criteria.

11.1.1 Definitions

Evaluable for toxicity. All patients will be evaluable for toxicity from the time of their first treatment with itraconazole and hydroxychloroquine.

Evaluable for objective response. Only those patients who have measurable disease present at baseline, have received at least one cycle of therapy, and have had their disease re-evaluated will be considered evaluable for response. These patients will have their response classified according to the definitions stated below. (Note: Patients who exhibit objective disease progression prior to the end of cycle 1 will also be considered evaluable.)

Evaluable Non-Target Disease Response. Patients who have lesions present at baseline that are evaluable but do not meet the definitions of measurable disease, have received at least one cycle of therapy, and have had their disease re-evaluated will be considered evaluable for non-target disease. The response assessment is based on the presence, absence, or unequivocal progression of the lesions.

11.1.2 Disease Parameters

Measurable disease. Measurable lesions are defined as those that can be accurately measured in at least one dimension (longest diameter to be recorded) as ≥20 mm by chest x-ray or as ≥10 mm with CT scan, MRI, or calipers by clinical exam. All tumor measurements must be recorded in millimeters (or decimal fractions of centimeters).

Note: Tumor lesions that are situated in a previously irradiated area might or might not be considered measurable. This will be determined by the investigator, and documented in source.

Malignant lymph nodes. To be considered pathologically enlarged and measurable, a lymph node must be ≥15 mm in short axis when assessed by CT scan (CT scan slice thickness recommended to be no greater than 5 mm). At baseline and in follow-up, only the short axis will be measured and followed.

Non-measurable disease. All other lesions (or sites of disease), including small lesions (longest diameter <10 mm or pathological lymph nodes with ≥10 to <15 mm short axis), are considered non-measurable disease. Bone lesions, leptomeningeal disease, ascites, pleural/pericardial effusions, lymphangitis cutis/pulmonitis, inflammatory breast disease, and abdominal masses (not followed by CT or MRI), are considered as non-measurable.

Note: Cystic lesions that meet the criteria for radiographically defined simple cysts should not be considered as malignant lesions (neither measurable nor non-measurable) since they are, by definition, simple cysts.

'Cystic lesions' thought to represent cystic metastases can be considered as measurable lesions, if they meet the definition of measurability described above. However, if non-cystic lesions are present in the same patient, these are preferred for selection as target lesions.

Target lesions. All measurable lesions up to a maximum of 2 lesions per organ and 5 lesions in total, representative of all involved organs, should be identified as **target lesions** and recorded and measured at baseline. Target lesions should be selected on the basis of their size (lesions with the longest diameter), be representative of all involved organs, but in addition should be those that lend themselves to reproducible repeated measurements. It may be the case that, on occasion, the largest lesion does not lend itself to reproducible measurement in which circumstance the next largest lesion which can be measured reproducibly should be selected. A sum of the diameters (longest for non-nodal lesions, short axis for nodal lesions) for all target lesions will be calculated and reported as the baseline sum diameters. If lymph nodes are to be included in the sum, then only the short axis is added into the sum. The baseline sum diameters will be used as reference to further characterize any objective tumor regression in the measurable dimension of the disease.

Non-target lesions. All other lesions (or sites of disease) including any measurable lesions over and above the 5 target lesions should be identified as **non-target lesions** and should also be recorded at baseline. Measurements of these lesions are not required, but the presence, absence, or in rare cases unequivocal progression of each should be noted throughout follow-up.

11.1.3 Methods for Evaluation of Measurable Disease

All measurements should be taken and recorded in metric notation using a ruler or calipers. All baseline evaluations should be performed as closely as possible to the beginning of treatment and never more than 4 weeks before the beginning of the treatment.

The same method of assessment and the same technique should be used to characterize each identified and reported lesion at baseline and during follow-up. Imaging-based evaluation is preferred to evaluation by clinical examination unless the lesion(s) being followed cannot be imaged but are assessable by clinical exam.

Clinical lesions Clinical lesions will only be considered measurable when they are superficial (*e.g.,* skin nodules and palpable lymph nodes) and ≥10 mm diameter as assessed using calipers (*e.g.,* skin nodules). In the case of skin lesions, documentation by color photography, including a ruler to estimate the size of the lesion, is recommended.

Chest x-ray Lesions on chest x-ray are acceptable as measurable lesions when they are clearly defined and surrounded by aerated lung. However, CT is preferable.

Conventional CT and MRI This guideline has defined measurability of lesions on CT scan based on the assumption that CT slice thickness is 5 mm or less. If CT scans have slice thickness greater than 5 mm, the minimum size for a measurable lesion should be twice the slice thickness. MRI is also acceptable in certain situations (*e.g.* for body scans).

Use of MRI remains a complex issue. MRI has excellent contrast, spatial, and temporal resolution; however, there are many image acquisition variables involved in MRI, which greatly impact image quality, lesion conspicuity, and measurement. Furthermore, the availability of MRI is variable globally. As with CT, if an MRI is performed, the technical specifications of the scanning sequences used should be optimized for the evaluation of the type and site of disease. Furthermore, as with CT, the modality used at follow-up should be the same as was used at baseline and the lesions should be measured/assessed on the same pulse sequence. It is beyond the scope of the RECIST guidelines to prescribe specific MRI pulse sequence parameters for all scanners, body parts, and diseases. Ideally, the same type of scanner should be used and the image acquisition protocol should be followed as closely as possible to prior scans. Body scans should be performed with breath-hold scanning techniques, if possible.

Ultrasound Ultrasound is not useful in assessment of lesion size and should not be used as a method of measurement. Ultrasound examinations cannot be reproduced in their entirety for independent review at a later date and, because they are operator dependent, it cannot be guaranteed that the same technique and measurements will be taken from one assessment to the next. If new lesions are identified by ultrasound in the course of the study, confirmation by CT or MRI is advised. If there is concern about radiation exposure at CT, MRI may be used instead of CT in selected instances.

Endoscopy, Laparoscopy The utilization of these techniques for objective tumor evaluation is not advised. However, such techniques may be useful to confirm complete pathological response when biopsies are obtained or to determine relapse in trials where recurrence following complete response (CR) or surgical resection is an endpoint.

Tumor markers Tumor markers alone cannot be used to assess response. If markers are initially above the upper normal limit, they must normalize for a patient to be considered in complete clinical response.

Cytology, Histology These techniques can be used to differentiate between partial responses (PR) and complete responses (CR) in rare cases (*e.g.*, residual lesions in tumor types, such as germ cell tumors, where known residual benign tumors can remain).

The cytological confirmation of the neoplastic origin of any effusion that appears or worsens during treatment when the measurable tumor has met criteria for response or stable disease is mandatory to differentiate between response or stable disease (an effusion may be a side effect of the treatment) and progressive disease.

11.1.4 Response Criteria

11.1.4.1 Evaluation of Target Lesions

Complete Response (CR): Disappearance of all target lesions. Any pathological lymph nodes (whether target or non-target) must have reduction in short axis to <10 mm.

Partial Response (PR): At least a 30% decrease in the sum of the diameters of target lesions, taking as reference the baseline sum diameters.

Progressive Disease (PD): At least a 20% increase in the sum of the diameters of target lesions, taking as reference the smallest sum on study (this includes the baseline sum if that is the smallest on study). In addition to the relative increase of 20%, the sum must also demonstrate an absolute increase of at least 5 mm. (Note: the appearance of one or more new lesions is also considered progressions).

Stable Disease (SD): Neither sufficient shrinkage to qualify for PR nor sufficient increase to qualify for PD, taking as reference the smallest sum diameters while on study, including baseline. Note that patients are not able to go from PR to SD.

11.1.4.2 Evaluation of Non-Target Lesions

Complete Response (CR): Disappearance of all non-target lesions and normalization of tumor marker level. All lymph nodes must be non-pathological in size (<10 mm short axis).

Note: If tumor markers are initially above the upper normal limit, they must normalize for a patient to be considered in complete clinical response.

Non-CR/Non-PD: Persistence of one or more non-target lesion(s) and/or maintenance of tumor marker level above the normal limits.

Progressive Disease (PD): Appearance of one or more new lesions and/or *unequivocal progression* of existing non-target lesions. *Unequivocal progression* should not normally trump target lesion status. It must be representative of overall disease status change, not a single lesion increase.

Although a clear progression of "non-target" lesions only is exceptional, the opinion of the treating physician should prevail in such circumstances, and the progression status should be confirmed at a later time by the review panel (or Principal Investigator).

11.1.4.3 Evaluation of Best Overall Response

The best overall response is the best response recorded from the start of the treatment until disease progression/recurrence (taking as reference for progressive disease the smallest measurements recorded since the treatment started). The patient's best response assignment will depend on the achievement of both measurement and confirmation criteria.

For Patients with Measurable Disease (*i.e.*, Target Disease)

Target Lesions	Non-Target Lesions	New Lesions	Overall Response
CR	CR	No	CR
CR	Non-CR/Non-PD	No	PR
CR	Not evaluated	No	PR
PR	Non-CR/Non-PD/not evaluated	No	PR
SD	Non-CR/Non-PD/not evaluated	No	SD
PD	Any	Yes or No	PD
Any	PD***	Yes or No	PD
Any	Any	Yes	PD

*** In exceptional circumstances, unequivocal progression in non-target lesions may be accepted as disease progression.

Note: Patients with a global deterioration of health status requiring discontinuation of treatment without objective evidence of disease progression at that time should be reported as "*symptomatic deterioration.*" Every effort should be made to document the objective progression even after discontinuation of treatment.

For Patients with Non-Measurable Disease (*i.e.*, Non-Target Disease)

Non-Target Lesions	New Lesions	Overall Response
CR	No	CR
Non-CR/non-PD	No	Non-CR/non-PD*
Not all evaluated	No	not evaluated
Unequivocal PD	Yes or No	PD
Any	Yes	PD
* 'Non-CR/non-PD' is preferred over 'stable disease' for non-target disease since SD is increasingly used as an endpoint for assessment of efficacy in some trials so to assign this category when no lesions can be measured is not advised		

11.1.5 Duration of Response

Duration of overall response: The duration of overall response is measured from the time measurement criteria are met for CR or PR (whichever is first recorded) until the first date that recurrent or progressive disease is objectively documented (taking as reference for progressive disease the smallest measurements recorded since the treatment started).

The duration of overall CR is measured from the time measurement criteria are first met for CR until the first date that progressive disease is objectively documented.

Duration of stable disease: Stable disease is measured from the start of the treatment until the criteria for progression are met, taking as reference the smallest measurements recorded since the treatment started, including the baseline measurements.

11.1.6 Progression-Free Survival

PFS is defined as the duration of time from start of treatment to time of progression or death, whichever occurs first.

11.1.7 Overall Response Rate

The ORR is defined as the proportion of patients achieving complete response (CR) or PR as assessed by the Investigator per RECIST (v.1.1).

11.1.8 Response Review

Response review will be done by central review at the Princess Margaret Cancer Centre by a trained radiologist.

12. DATA REPORTING / REGULATORY REQUIREMENTS

Adverse event lists, guidelines, and instructions for AE reporting can be found in Section 7.0 (Adverse Events).

12.1 Data Collection and Reporting

All data obtained in the clinical trial described in this protocol will be reported on eCRFs in the Medidata Electronic Document Capture system (Medidata). Data reported on eCRFs should be consistent with the source documents and verifiable. All data for the primary and secondary endpoints will source verified prior to publication. The Investigator will review the data and electronically sign the eCRFs to acknowledge agreement with the data entered. Data will be entered into Medidata will be used for developing tables and listings for the final study report.

Prior to the start of the study, the Investigator will complete a Site Particpant's Log showing the signatures and handwritten initials of all individuals who are authorized to make or change entries on source documents and eCRFs.

12.2 Source Documents

Source documents refer to the original documents, data, and records where the first recording of a data point occurred. Examples of source documentation include, but are not limited to:

> Hospital records, clinical and office charts, laboratory notes, memoranda, subjects' diaries or evaluation checklists, pharmacy dispensing records, recorded data from automated instruments, copies or transcriptions certified after verification as being accurate copies, microfiches, photographic negatives, microfilm or magnetic media, x-rays, subject files, and records kept at the pharmacy, at the laboratories and at medico-technical departments involved in the clinical trial)

Please ensure that source document entries are attributable, legible, contemporaneous, original, and accurate. Note that sign-off of source documents should be attributable to a single record and "bracketing" multiple entries on source document pages for a single signature is not allowed. Corrections to source document entries should only be completed by drawing a single line through the previous entry and then recording the corrected data, initialing the change, and dating the change. Only the individual that initially recorded the data should make any corrections.

12.3 Retention of Patient Records and Study Files

The ICH guidance document, Good Clinical Practice: Consolidated Guidelines (ICH Guidance Document E6) (1997) states that the investigator and sponsor shall retain study records relating to the study until at least 2 years after the last approval of a marketing application and until there are no pending or contemplated marketing applications, or at least 2 years have elapsed since the

formal discontinuation of clinical development of the investigational product. In the event of a trial discontinuation, sponsor records should also be kept for a minimum of 2 years. Per Health Canada, all original records should be maintained for 25 years after the above requirements are satisfied and the final report has been issued. Records contained in the Clinical Trial Application should be maintained on file for at least 25 years. We will comply with these regulations. The Sponsor will notify sites when documents are to be destroyed.

12.4 Site and Study Closure

Upon completion of the study, the following activities, when applicable, will be completed by the Central Office in conjunction with the Investigator, as appropriate:

- Collection of study materials (i.e., specimen collection kits, drug shippers, etc.)
- Data clarifications and/or resolutions
- Accounting, reconciliation, and final disposition of used and unused study medication
- Review of site study records for completeness

If the Sponsor- Investigator or appropriate regulatory officials identify conditions arising during the study that indicate that the study should be halted or that the study center should be terminated, this action may be taken after appropriate consultation among the Sponsor and Investigator. Conditions that may warrant termination of the study include, but are not limited to, the following:

- The discovery of an unexpected, serious, or unacceptable risk to the patients enrolled in the study
- A decision on the part of the Sponsor to suspend or discontinue testing, evaluation, or development of the product
- Failure of the Investigator to enroll patients into the study at an acceptable rate
- Failure of the Investigator to comply with pertinent regulations of appropriate regulatory authorities
- Submission of knowingly false information to the Sponsor, or appropriate regulatory authority
- Insufficient adherence to protocol requirements
- Refusal of the Investigator to supply source documentation of work performed in this clinical trial

Study termination and follow-up will be performed in compliance with the conditions set forth in the International Conference on Harmonisation (ICH) sixth efficacy publication (E6) on Good Clinical Practice, Section 4.12, ICH E6 4.13, ICH E6 5.20, and ICH E6 5.21.

13. STATISTICAL CONSIDERATIONS

13.1 Study Design/Endpoints

The rate of subject entry and escalation to the next dose level will depend upon assessment of the safety profile of patients entered at the previous dose level. Toxicity will be evaluated according to the NCI Common Terminology Criteria for Adverse Events (CTCAE), Version 4.0.

Up to six evaluable patients will be treated at a given dose level combination and observed for at least 4 weeks from start of treatment to assess toxicity for purposes of determining dose limiting toxicities (DLTs). When exactly 6 patients have been enrolled on a given dose level and it is unknown whether the MTD has been exceeded or not, accrual will be suspended until enough toxicity data are available to decide (i.e. to determine if the dose level has at least 2 DLTs versus less than 2).

13.1.1 Phase I Study Design

A rolling six phase I design [47] will be used to accelerate the process of finding the maximum tolerated dose of hydroxychloroquine in combination with itraconazole given that these two drugs have been extensively studied and registered for other indications and that no drug-drug interactions are known to exist.

The starting dose of hydroxychloroquine of 200mg PO BID has been chosen as this is a clinically active dose in other disease entities and the maximum dose we wish to safely achieve is 600mg PO BID as studied in other cancer sites in combination with other anti-cancer therapies such as temsirolimus [33].

Dose-limiting toxicities have been defined in section 5.2. The rules for dose assignment and determination of MTD and RP2D are described in section 5.3.

13.1.2 Phase II study design

Once the dose level for hydroxychloroquine and combination itraconazole has been determined, the cohort will be expanded in a single-centre, single-arm, Simon two-stage design.

The phase II cohort will consist of a maximum of additional 34 patients. If 1 response is observed after 9 patients have been treated at RP2D, then an additional 25 patients will be enrolled.

CT scans will be performed every 8 weeks regardless of dosing delays.

The phase II cohort will serve to investigate ORR based on RECIST criteria (refer to).

13.1.3 Study Endpoints

The primary endpoint will be the maximum tolerated dose (or maximum administered dose) of hydroxychloroquine in combination with -300 mg PO BID of itraconazole for the recommended phase II dose.

The secondary endpoint of PFS and ORR have been defined in section 11.1.6 and 11.1.7 respectively.

13.2 Sample Size/Accrual Rate

The sample size will be a maximum of 52 patients. This will encompass between 6 and 18 on phase I and 34 on for the phase II component.

Phase II sample size*:*

Stage 1 of accrual: 9 response evaluable patients will be entered in the first stage. Using response hypotheses of H_0 <15 % and H_a >35%, we would reject the drug at the end of the first stage of accrual if no objective responses were seen. Otherwise, an additional 25 patients will be accrued. Patients treated at RP2D in the phase 1 part of the study will be considered for the statistical analysis of the phase 2 stage 1. A total (between phase 1 and phase 2 stage 1) of 9 response evaluable patients treated at RP2D are required.

Stage 2 of accrual: An additional 25 patients will be accrued. We would accept the drug as active if 8 or more objective responses are observed from 25 patients accrued.

Significance level and power: The procedure described above test the null hypothesis that the response rate is 15% versus alternating hypotheses that the response rate is 35%. The significance level (*i.e.*, the probability of rejecting H0 when it is true) is α=0.05 and the power (*i.e.*, the probability of deciding the regimen is active) is 0.81 when true response rate is 20%. The expected sample size with this design is 19 when the null hypothesis is true and 25 when the alternative hypothesis is true.

Accrual and Duration of Study
The estimated accrual for this study is 2-4 patients a month. Thus, patient accrual is expected to be completed within 12-18 months. Additional time is required to allow the response data to mature.

All of the patients registered in the study will be accounted for. The number of patients who were not evaluable, who died or withdrew before treatment began will be specified. The distribution of follow-up time will be described and the number of patients lost to follow-up will be given.

13.2.1 Patient Replacement

If a patient is withdrawn from the study prior to completing 28 days of therapy without experiencing a DLT prior to withdrawal, an additional patient may be added to that dose level. Patients missing 7 or more days due to toxicity will not be replaced since these patients will be considered to have experienced a dose limiting toxicity.

13.3 Stratification Factors

No stratification will be used for this trial.

13.4 Analysis of Secondary Endpoints

Summary statistics, such as mean, median, counts and proportion, will be used to summarize the patients. Survival estimates will be computed using the Kaplan-Meier method. Potential association between variables will be measured using Pearson correlation coefficients, chi-square tests, one- or two-sample t-tests or logistic regression analyses as appropriate. Non-parametric tests such as Spearman correlation coefficients, Fisher's exact tests and Wilcoxon rank sum test may be substituted if necessary. Ninety-five percent confidence intervals will be constructed and selected results will be illustrated using figures and plots.

Frequency and severity of adverse events will be tabulated using counts and proportions detailing frequently occurring, serious and severe events of interest.

13.5 Reporting and Exclusions

13.5.1 Evaluation of Toxicity

All patients will be evaluable for toxicity from the time of their first treatment with itraconazole and hydroxychloroquine.

13.5.2 Evaluation of Response

All patients included in the study must be assessed for response to treatment, even if there are major protocol treatment deviations or if they are ineligible. Each patient will be assigned one of the following categories: 1) complete response, 2) partial response, 3) stable disease, 4) progressive disease, 5) early death from malignant disease, 6) early death from toxicity, 7) early death because of other cause, or 9) unknown (not assessable, insufficient data). [Note: By arbitrary convention, category 9 usually designates the "unknown" status of any type of data in a clinical database.]

All of the patients who met the eligibility criteria (with the possible exception of those who received no study medication) should be included in the main analysis of the response rate. Patients in response categories 4-9 should be considered to have a treatment failure (disease progression). Thus, an incorrect treatment schedule or drug administration does not result in exclusion from the analysis of the response rate. Precise definitions for categories 4-9 will be protocol specific.

All conclusions should be based on all eligible patients. Subanalyses may then be performed on the basis of a subset of patients, excluding those for whom major protocol deviations have been identified (*e.g.*, early death due to other reasons, early discontinuation of treatment, major protocol violations, etc.). However, these subanalyses may not serve as the basis for drawing conclusions concerning treatment efficacy, and the reasons for excluding patients from the analysis should be clearly reported. The 95% confidence intervals should also be provided.

REFERENCES

1. Navaneelan, T., *Trends in the incidence and mortality of female reproductive system cancers. Health at a Glance.* Statistics Canada catalogue 201. **no. 82-624-X.**
2. Fung-Kee-Fung, M., et al., *Optimal chemotherapy treatment for women with recurrent ovarian cancer.* Curr Oncol, 2007. **14**(5): p. 195-208.
3. Tania, M., M.A. Khan, and Y. Song, *Association of lipid metabolism with ovarian cancer.* Curr Oncol, 2010. **17**(5): p. 6-11.
4. Pyragius, C.E., et al., *Aberrant lipid metabolism: an emerging diagnostic and therapeutic target in ovarian cancer.* Int J Mol Sci, 2013. **14**(4): p. 7742-56.
5. Hedditch, E.L., et al., *ABCA transporter gene expression and poor outcome in epithelial ovarian cancer.* J Natl Cancer Inst, 2014. **106**(7).
6. Cheong, H., et al., *Therapeutic targets in cancer cell metabolism and autophagy.* Nat Biotechnol, 2012. **30**(7): p. 671-8.
7. Yang, Z.J., et al., *The role of autophagy in cancer: therapeutic implications.* Mol Cancer Ther, 2011. **10**(9): p. 1533-41.
8. Orfanelli, T., et al., *Involvement of autophagy in cervical, endometrial and ovarian cancer.* Int J Cancer, 2014. **135**(3): p. 519-28.
9. Bao, L., et al., *Induction of autophagy contributes to cisplatin resistance in human ovarian cancer cells.* Mol Med Rep, 2015. **11**(1): p. 91-8.
10. Manic, G., et al., *Chloroquine and hydroxychloroquine for cancer therapy.* Molecular & Cellular Oncology, 2014. **1**(1): p. e29911.
11. Xu, J., et al., *Cholesterol trafficking is required for mTOR activation in endothelial cells.* Proc Natl Acad Sci U S A, 2010. **107**(10): p. 4764-9.
12. Strating, J.R., et al., *Itraconazole inhibits enterovirus replication by targeting the oxysterol-binding protein.* Cell Rep, 2015. **10**(4): p. 600-15.
13. Liu, R., et al., *Itraconazole suppresses the growth of glioblastoma through induction of autophagy: involvement of abnormal cholesterol trafficking.* Autophagy, 2014. **10**(7): p. 1241-55.
14. Ikonen, E., *Cellular cholesterol trafficking and compartmentalization.* Nat Rev Mol Cell Biol, 2008. **9**(2): p. 125-38.
15. Aftab, B.T., et al., *Itraconazole inhibits angiogenesis and tumor growth in non-small cell lung cancer.* Cancer Res, 2011. **71**(21): p. 6764-72.
16. Kim, J., et al., *Itraconazole, a commonly used antifungal that inhibits Hedgehog pathway activity and cancer growth.* Cancer Cell, 2010. **17**(4): p. 388-99.
17. Chong, C.R., et al., *Inhibition of angiogenesis by the antifungal drug itraconazole.* ACS Chem Biol, 2007. **2**(4): p. 263-70.
18. Pantziarka, P., et al., *Repurposing Drugs in Oncology (ReDO)-itraconazole as an anti-cancer agent.* Ecancermedicalscience, 2015. **9**: p. 521.
19. Tsubamoto, H., et al., *Impact of combination chemotherapy with itraconazole on survival of patients with refractory ovarian cancer.* Anticancer Res, 2014. **34**(5): p. 2481-7.
20. Vanden Bossche, H., L. Koymans, and H. Moereels, *P450 inhibitors of use in medical treatment: focus on mechanisms of action.* Pharmacol Ther, 1995. **67**(1): p. 79-100.
21. Vanden Bossche, H., P. Marichal, and F.C. Odds, *Molecular mechanisms of drug resistance in fungi.* Trends Microbiol, 1994. **2**(10): p. 393-400.

22. Antonarakis, E.S., et al., *Repurposing itraconazole as a treatment for advanced prostate cancer: a noncomparative randomized phase II trial in men with metastatic castration-resistant prostate cancer.* Oncologist, 2013. **18**(2): p. 163-73.
23. Rudin, C.M., et al., *Phase 2 study of pemetrexed and itraconazole as second-line therapy for metastatic nonsquamous non-small-cell lung cancer.* J Thorac Oncol, 2013. **8**(5): p. 619-23.
24. Kim, D.J., et al., *Open-label, exploratory phase II trial of oral itraconazole for the treatment of basal cell carcinoma.* J Clin Oncol, 2014. **32**(8): p. 745-51.
25. Mudge, S.J., D. Hayes, and D.H. Ellis, *ingle-dose phase 1 studies to evaluate the more predictable pharmacokinetics of itraconazole in a novel capsule formulation (Lozanoc™) relative to conventional itraconazole capsule formulation.* 19th Congress of the International Society for Human and Animal Mycology
Conference abstracts, 2015.
26. Caputo, R., *Itraconazole (Sporanox) in superficial and systemic fungal infections.* Expert Rev Anti Infect Ther, 2003. **1**(4): p. 531-42.
27. Matthieu, L., et al., *Itraconazole penetrates the nail via the nail matrix and the nail bed--an investigation in onychomycosis.* Clin Exp Dermatol, 1991. **16**(5): p. 374-6.
28. Heykants, J., et al., *The clinical pharmacokinetics of itraconazole: an overview.* Mycoses, 1989. **32 Suppl 1**: p. 67-87.
29. Haria, M., H.M. Bryson, and K.L. Goa, *Itraconazole. A reappraisal of its pharmacological properties and therapeutic use in the management of superficial fungal infections.* Drugs, 1996. **51**(4): p. 585-620.
30. Poirier, J.M. and G. Cheymol, *Optimisation of itraconazole therapy using target drug concentrations.* Clin Pharmacokinet, 1998. **35**(6): p. 461-73.
31. Rangwala, R., et al., *Phase I trial of hydroxychloroquine with dose-intense temozolomide in patients with advanced solid tumors and melanoma.* Autophagy, 2014. **10**(8): p. 1369-79.
32. Briceno, E., S. Reyes, and J. Sotelo, *Therapy of glioblastoma multiforme improved by the antimutagenic chloroquine.* Neurosurg Focus, 2003. **14**(2): p. e3.
33. Rangwala, R., et al., *Combined MTOR and autophagy inhibition: phase I trial of hydroxychloroquine and temsirolimus in patients with advanced solid tumors and melanoma.* Autophagy, 2014. **10**(8): p. 1391-402.
34. Rosenfeld, M.R., et al., *A phase I/II trial of hydroxychloroquine in conjunction with radiation therapy and concurrent and adjuvant temozolomide in patients with newly diagnosed glioblastoma multiforme.* Autophagy, 2014. **10**(8): p. 1359-68.
35. Tett, S., et al., *Insights from pharmacokinetic and pharmacodynamic studies of hydroxychloroquine.* Agents Actions Suppl, 1993. **44**: p. 145-90.
36. Lee, J.Y., et al., *Association of Polymorphisms of Cytochrome P450 2D6 With Blood Hydroxychloroquine Levels in Patients With Systemic Lupus Erythematosus.* Arthritis Rheumatol, 2016. **68**(1): p. 184-90.
37. Head, S.A., et al., *Simultaneous Targeting of NPC1 and VDAC1 by Itraconazole Leads to Synergistic Inhibition of mTOR Signaling and Angiogenesis.* ACS Chem Biol, 2017. **12**(1): p. 174-182.
38. Adams, O., et al., *Prognostic relevance of autophagy markers LC3B and p62 in esophageal adenocarcinomas.* Oncotarget, 2016. **7**(26): p. 39241-39255.

39. Oza, A.M., et al., *Standard chemotherapy with or without bevacizumab for women with newly diagnosed ovarian cancer (ICON7): overall survival results of a phase 3 randomised trial.* Lancet Oncol, 2015. **16**(8): p. 928-36.
40. Pujade-Lauraine, E., et al., *Bevacizumab combined with chemotherapy for platinum-resistant recurrent ovarian cancer: The AURELIA open-label randomized phase III trial.* J Clin Oncol, 2014. **32**(13): p. 1302-8.
41. Sun, Y., et al., *Modulatory effects of Beclin 1 on expression of angiopoietin and Tie-2 receptor in human cervical cancer cells.* Asian Pac J Cancer Prev, 2011. **12**(11): p. 2985-90.
42. Tsubamoto, H., et al., *Impact of combination chemotherapy with itraconazole on survival for patients with recurrent or persistent ovarian clear cell carcinoma.* Anticancer Res, 2014. **34**(4): p. 2007-14.
43. DeLisser, H.M., et al., *Involvement of endothelial PECAM-1/CD31 in angiogenesis.* Am J Pathol, 1997. **151**(3): p. 671-7.
44. Bryant, C.S., et al., *Reduction of hypoxia-induced angiogenesis in ovarian cancer cells by inhibition of HIF-1 alpha gene expression.* Arch Gynecol Obstet, 2010. **282**(6): p. 677-83.
45. Holohan, C., et al., *Cancer drug resistance: an evolving paradigm.* Nat Rev Cancer, 2013. **13**(10): p. 714-26.
46. Eisenhauer, E.A., et al., *New response evaluation criteria in solid tumours: revised RECIST guideline (version 1.1).* Eur J Cancer, 2009. **45**(2): p. 228-47.
47. Skolnik, J.M., et al., *Shortening the timeline of pediatric phase I trials: the rolling six design.* J Clin Oncol, 2008. **26**(2): p. 190-5.

APPENDIX A PERFORMANCE STATUS CRITERIA

ECOG Performance Status Scale		Karnofsky Performance Scale	
Grade	Descriptions	Percent	Description
0	Normal activity. Fully active, able to carry on all pre-disease performance without restriction.	100	Normal, no complaints, no evidence of disease.
		90	Able to carry on normal activity; minor signs or symptoms of disease.
1	Symptoms, but ambulatory. Restricted in physically strenuous activity, but ambulatory and able to carry out work of a light or sedentary nature (*e.g.*, light housework, office work).	80	Normal activity with effort; some signs or symptoms of disease.
		70	Cares for self, unable to carry on normal activity or to do active work.
2	In bed <50% of the time. Ambulatory and capable of all self-care, but unable to carry out any work activities. Up and about more than 50% of waking hours.	60	Requires occasional assistance, but is able to care for most of his/her needs.
		50	Requires considerable assistance and frequent medical care.
3	In bed >50% of the time. Capable of only limited self-care, confined to bed or chair more than 50% of waking hours.	40	Disabled, requires special care and assistance.
		30	Severely disabled, hospitalization indicated. Death not imminent.
4	100% bedridden. Completely disabled. Cannot carry on any self-care. Totally confined to bed or chair.	20	Very sick, hospitalization indicated. Death not imminent.
		10	Moribund, fatal processes progressing rapidly.
5	Dead.	0	Dead.

APPENDIX B INFORMATION ON POSSIBLE DRUG INTERACTIONS

Information on Possible Interactions with Other Agents for Patients and Their Caregivers and Non-Study Healthcare Team

The patient **____________________________** *is enrolled on a clinical trial using the experimental agent* **itraconazole and hydroxychloroquine**.

Itraconazole and hydroxychloroquine interact with many drugs that are processed by your liver. Because of this, it is very important to tell your study doctors about all of your medicine before you start this study. It is also very important to tell them if you stop taking any regular medicine, or if you start taking a new medicine while you take part in this study. When you talk about your medicine with your study doctor, include medicine you buy without a prescription at the drug store (over-the-counter remedy), or herbal supplements such as St. John's wort.

Many health care prescribers can write prescriptions. You must also tell your other prescribers (doctors, physicians' assistants or nurse practitioners) that you are taking part in a clinical trial. **Bring this paper with you and keep the attached information card in your wallet**. These are the things that you and they need to know:

Itraconazole interact with certain specific enzymes in your liver.

- The enzyme is CYP3A4, that induce and break down this drug in the liver.

- **Itraconazole** must be used very carefully with other medicines that need these liver enzymes to be effective or to be cleared from your system.
- Other medicines may also affect the activity of the enzyme.
  - Substances that increase the enzyme's activity ("inducers") could reduce the effectiveness of the drug, while substances that decrease the enzyme's activity ("inhibitors") could result in high levels of the active drug, increasing the chance of harmful side effects.
  - **Itraconazole** are considered a inducers of the enzymes, meaning that they can affect the levels of other drugs that are processed by that enzyme. This can lead to harmful side effects and/or reduce the effectiveness of those medications.
- You and healthcare providers who prescribe drugs for you must be careful about adding or removing any drug in this category.
- Before you start the study, your study doctor will work with your regular prescriber to switch any medicines that are considered "strong inducers/inhibitors of CYP3A4 or substrates of **CYP 3A4** that are sensitive or have a narrow therapeutic window**.**
- Your prescribers should look at this web site http://medicine.iupui.edu/clinpharm/ddis/table.aspx or consult a medical reference to see if any medicine they want to prescribe is on a list of drugs to avoid.
- Please be very careful! Over-the-counter drugs have a brand name on the label—it's usually big and catches your eye. They also have a generic name—it's usually small and located above or below the brand name, and printed in the ingredient list. Find the

generic name and determine, with the pharmacist's help, whether there could be an adverse interaction.

- Be careful:
  - If you take acetaminophen regularly: You should not take more than 4 grams a day if you are an adult or 2.4 grams a day if you are older than 65 years of age. Read labels carefully! Acetaminophen is an ingredient in many medicines for pain, flu, and cold.
  - If you drink grapefruit juice or eat grapefruit: Avoid these until the study is over.
  - If you take herbal medicine regularly: You should not take St. John's wort while you are taking **itraconazole and hydroxychloroquine**.

Other medicines can be a problem with your study drugs.

- You should check with your doctor or pharmacist whenever you need to use an over-the-counter medicine or herbal supplement.

- Your regular prescriber should check a medical reference or call your study doctor before prescribing any new medicine for you. Your study doctor's name is Dr. Stephanie Lheureux

____

and he or she can be contacted at 416-946-2818

____

APPENDIX C PROHIBITED DRUGS

alfentanil
avanafil
budesonide
buspirone
conivaptan
dapsone
darifenacin
darunavir
dasatinib
dronedarone
ebastine
eletriptan
eplerenone
everolimus
felodipine
ibrutinib
indinavir
lomitapide
lovastatin
lurasidone
maraviroc
midazolam
naloxegol
nisoldipine
quetiapine
saquinavir
sildenafil
simvastatin
sirolimus
tacrolimus
ticagrelor
tipranavir
tolvaptan
triazolam
Vardenafi
Other antimalaria agents

Drugs with Known Torsades de Pointes risk	Drugs with Possible Torsades the Pointes risk
Amiodarone	Alfuzosin
Anagrelide	Apomorphine
Arsenic trioxide	Aripiprazole
Astemizole	Artenimol+piperaquine
Azithromycin	Asenapine
Bepridil	Atomoxetine
Chloroquine	Bedaquiline
Chlorpromazine	Bendamustine
Cilostazol	Bortezomib
Ciprofloxacin	Bosutinib
Cisapride	Buprenorphine
Citalopram	Capecitabine
Clarithromycin	Ceritinib
Cocaine	Clomipramine
Disopyramide	Clozapine
Dofetilide	Crizotinib
Domperidone	Cyamemazine (cyamepromazine)
Donepezil	Dabrafenib
Dronedarone	Dasatinib
Droperidol	Degarelix
Erythromycin	Delamanid
Escitalopram	Desipramine
Flecainide	Dexmedetomidine
Fluconazole	Dolasetron
Gatifloxacin	Efavirenz
Grepafloxacin	Eribulin mesylate
Halofantrine	Ezogabine (Retigabine)
Haloperidol	Famotidine
Ibogaine	Felbamate
Ibutilide	Fingolimod
Levofloxacin	Flupentixol
Levomepromazine	Gemifloxacin
Levomethadyl acetate	Granisetron
Levosulpiride	Hydrocodone
Mesoridazine	Iloperidone
Methadone	Imipramine
Moxifloxacin	Isradipine
Ondansetron	Ketanserin

Oxaliplatin	Lapatinib
Papaverine HCl (Intra-coronary)	Lenvatinib
Pentamidine	Leuprolide
Pimozide	Lithium
Probucol	Melperone
Procainamide	Midostaurin
Propofol	Mifepristone
Quinidine	Mirabegron
Roxithromycin	Mirtazapine
Sevoflurane	Moexipril/HCTZ
Sotalol	Necitumumab
Sparfloxacin	Nicardipine
Sulpiride	Nilotinib
Sultopride	Norfloxacin
Terfenadine	Nortriptyline
Terlipressin	Nusinersen
Terodiline	Ofloxacin
Thioridazine	Osimertinib
Vandetanib	Oxytocin
	Paliperidone
	Panobinostat
	Pasireotide
	Pazopanib
	Perflutren
	Perphenazine
	Pilsicainide
	Pimavanserin
	Pipamperone
	Promethazine
	Prothipendyl
	Ribociclib
	Rilpivirine
	Risperidone
	Romidepsin
	Saquinavir
	Sertindole
	Sorafenib
	Sunitinib
	Tacrolimus
	Tamoxifen
	Telavancin

	Telithromycin<br>Tetrabenazine<br>Tiapride<br>Tizanidine<br>Tolterodine<br>Toremifene<br>Trimipramine<br>Tropisetron<br>Vardenafil<br>Vemurafenib<br>Venlafaxine<br>Vorinostat<br>Zotepine

These lists are not exhaustive and the user should contact a frequently updated medical/drug information reference

APPENDIX D SAMPLE WALLET CARD

**INFORMATION ON POSSIBLE DRUG INTERACTIONS**<br>You are enrolled on a clinical trial using the experimental agents **itraconazole and hydroxychloroquine. Itraconazole and hydroxychloroquine** interact with drugs that are processed by your liver. Because of this, it is very important to:<br>➢ Tell your doctors if you stop taking regular medicine or if you start taking a new medicine.<br>➢ Tell all of your prescribers (doctor, physicians' assistant, nurse practitioner, pharmacist) that you are taking part in a clinical trial.<br>➢ Check with your doctor or pharmacist whenever you need to use an over-the-counter medicine or herbal supplement.	**Itraconazole** interacts with a specific liver enzyme called **CYP 3A4**, and hydroxychloroquine interacts with a specific liver enzyme called **CYP 2D6** and they must be used very carefully with other medicines that interact with these enzymes.<br>➢ Before you start the study, your study doctor will work with your regular prescriber to switch any medicines that are considered "strong inducers/inhibitors or substrates of **CYP 3A4 & CYP 2D6**."<br>➢ Before prescribing new medicines, your regular prescribers should go to http://medicine.iupui.edu/clinpharm/ddis/table.aspx for a list of drugs to avoid, or contact your study doctor.<br>➢ Your study doctor's name is ______________________________ and can be contacted at ________________________________.

APPENDIX E SAMPLE PILL DIARY

Protocol Number: HYDRA-1
Patient's Diary Card: Cycle _______

Date: _____________Patient Initials:_____________ Patient Study ID: _____________

INSTRUCTIONS

1. Complete one form for each cycle of treatment.
2. You will take ____ itraconazole tablets (____ mg) orally with food every 12 hours. Your total dose for the day will be ____ mg.
3. You will take _____ hydroxychloroquine tablets (_____ mg) orally with food every 12 hours. Your total dose for the day will be ____ mg.
4. Record the date and time you took the tablet, and number of tablets taken. Try to take the tablets at the same time each day.
5. If you vomit after taking a dose, do not take another tablet.
6. Swallow each tablet whole. Do not crush or chew the tablets.
7. If you have any comments or notice any side effects, please record them in the comments column.
8. Please bring this form and your bottles of itraconazole and hydroxychloroquine at the end of each cycle.
9. In case of errors, please place a single slash mark through the error and initial it. Please do not white out any error or scribble it out with ink. Please do not write the correct information directly over the error, but on a separate line next to the error.
10.

Day	Date	Time	Number of intraconazole tablets taken	Number of hydroxychloroquine tablets taken	Comments
**1**					
**2**					
**3**					
**4**					
**5**					
**6**					
**7**					
**8**					
**9**					
**10**					

**11**					
**12**					
**13**					
**14**					
**15**					
**16**					
**17**					
**18**					
**19**					
**20**					
**21**					
**22**					
**23**					
**24**					
**25**					
**26**					
**27**					
**28**					

Signature of Reviewer: ______________________________ Date of Review: _________________

APPENDIX F DATA MANAGEMENT GUIDELINES

Data Management Guidelines

Case Report Form Submission Schedule

The Registration Checklist will be a paper CRF that will be provided by the Drug Development Central Office and all other data required for the study will be collected in eCRFs in Medidata. The form submission schedule is outlined below.

Case Report Form	Submission Schedule
Registration Checklist	At the time of registration
Baseline Form	Within 3 weeks of on study date
On Treatment Form	Within 3 weeks of the end of each cycle of treatment
Off Treatment Form	Within 3 weeks of the patient coming off-study
Short Follow-up Form	Within 3 weeks of the patient coming to clinic. Required every 4 weeks until 12 weeks post off-treatment visit.
Final Report Form	Within 3 weeks of the patient's completion on study

Case Report Form Completion

The paper Registration Checklist must be completed using black or blue ink. Any errors must be crossed out so that the original entry is still visible, the correction clearly indicated and then initialed and dated by the individual making the correction. eCRFs will be completed according to the schedule noted above.

All patient names or other identifying information will be removed prior to being sent to the Central Office and the documents labeled with patient initials, study number and the protocol number if applicable.

Monitoring

This is an investigator initiated study and study monitoring will be performed by the Drug Development Program Central Office or its designate.

Data in the Medidata Rave eCRFs will be monitored on a regular basis and quality assurance measures will be performed. Electronic data queries as well as paper query letters may be issued to the site.

Regulatory Requirements

- Please submit all required documents to the DDP Central Office.

- Canadian Principal Investigators must submit a completed Qualified Investigator Undertaking. The original signed copy is to be housed by the DDP Central Office.
- All investigators must have an up-to-date CV (signed within 2 years) on file with the DDP Central Office.
- Laboratory certification/accreditation and normal ranges are required
- Confirmation of all investigators having undergone training in the Protection of Human Research Subjects is required. It is preferred that other staff involved in the trial also undergoes such training.
- Investigators and site staff are required to complete Medidata eCRF training modules depending on delegated tasks
- Consent forms must be reviewed by the Central Office before submission to the local ethics regulatory board (REB/IRB) and must include a statement that 1) information will be sent to and 2) medical records will be reviewed by the DDP Central Office.
- A Membership list of the local ethics board is required.
- A copy of the initial approval letter from the ethics board must be submitted to the DDP Central Office.
- A completed Site Participant List/Training Log is required and must be submitted to DDP
- Continuing approval will be obtained at least yearly until follow-up on patients is completed and no further data is being obtained for research purposes.
