# Supplementary material for: Repurposing Itraconazole and Hydroxychloroquine to Target Lysosomal Homeostasis in Epithelial Ovarian Cancer
Source: Cancer Res Commun. 2022 May 4;2(5):293–306. doi: 10.1158/2767-9764.CRC-22-0037 (PMC9981200; doi:10.1158/2767-9764.CRC-22-0037)
Supplement: Supplementary Figures 1-7 — Supplementary Figure 1: Itra dose response in ovarian cancer cell lines. - Supplementary Figure 2: Analysis on ovarian cancer cell lines used in the Itra-sensitizing CRISPR screen (OVCAR5 and TOV1946). - Supplementary Figure 3: c18orf8 and VPS54 Knockout effects on the TOV1946 cell line. - Supplementary Figure 4: Itra+CQ dose responses. - Supplementary Figure 5: 5/10 uM CQ response and apoptosis assay results. - Supplementary Figure 6: Itra+CQ dose response in c18orf8 and VPS54 knockout cells and calculation of synergy scores. - Supplementary Figure 7: Intratumoural Itra/CQ detection and Ki-67 analysis in patient samples. [file crc-22-0037-s03.pdf]

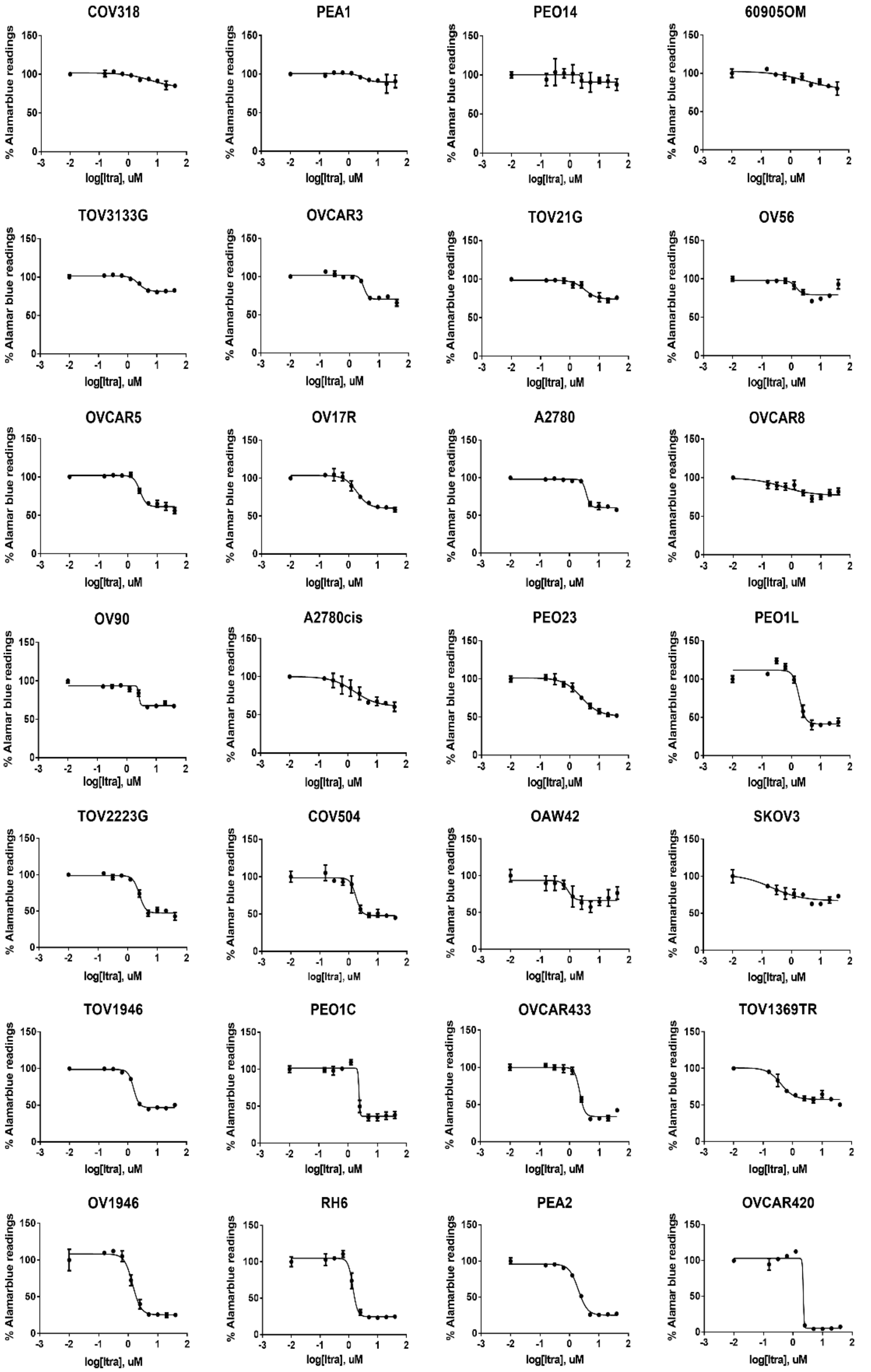

Supplementary Figure 1. Itraconazole (Itra) dose response in ovarian cancer cell lines. Graphs showing Alamar blue dose responses on cells treated with Itra at a concentration of 0-40μmol/L for 5 days. (N= 3 biological replicates).

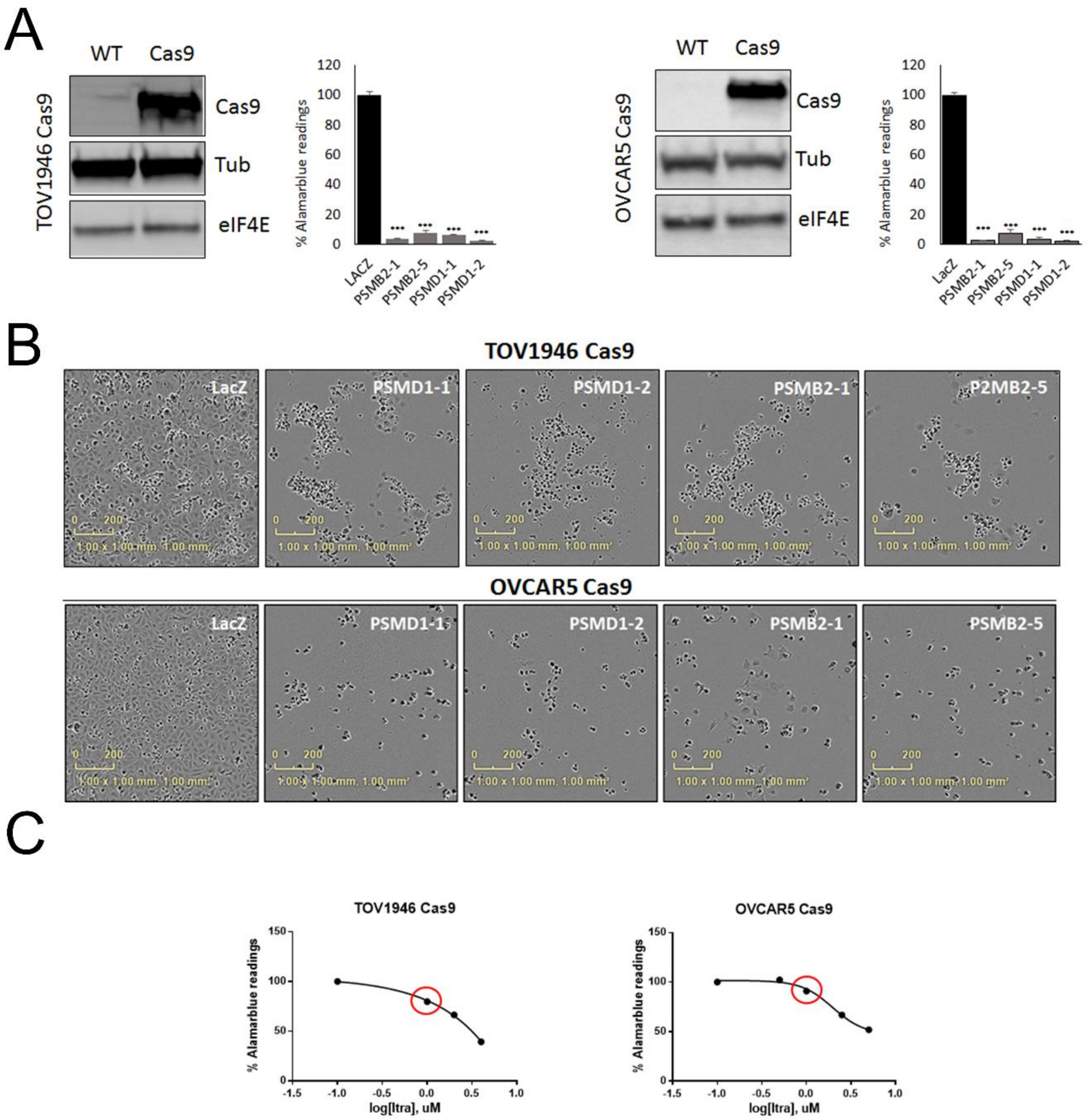

Supplementary Figure 2. Analysis on ovarian cancer cell lines used in the Itra-sensitizing CRISPR screen. **A,** Western blotting analysis showing Cas9 expression in stable TOV1946 and OVCAR5 cells overexpressing Cas9. B-tubulin and eIF4E were used as loading controls. Alamar blue results showing Cas9 activity in Cas9 overexpressing cells infected to express sgRNA targeting *LacZ* (control condition) or the proteasomal subunits *PSMD1* and *PSMD2* previously reported to be essential genes. 2 different sgRNA were used for *PSMD1* and *PSMD2*. **B**, Representative pictures of cells treated in the same conditions as **A**. **C**, Alamar blue results showing Itra activity in OVCAR5 and TOV1946 overexpressing Cas9. 1 μmol/L Itra condition is circled in red. *P < 0.05, **P < 0.01, ***P < 0.001, ****P < 0.0001; ns, not significant.

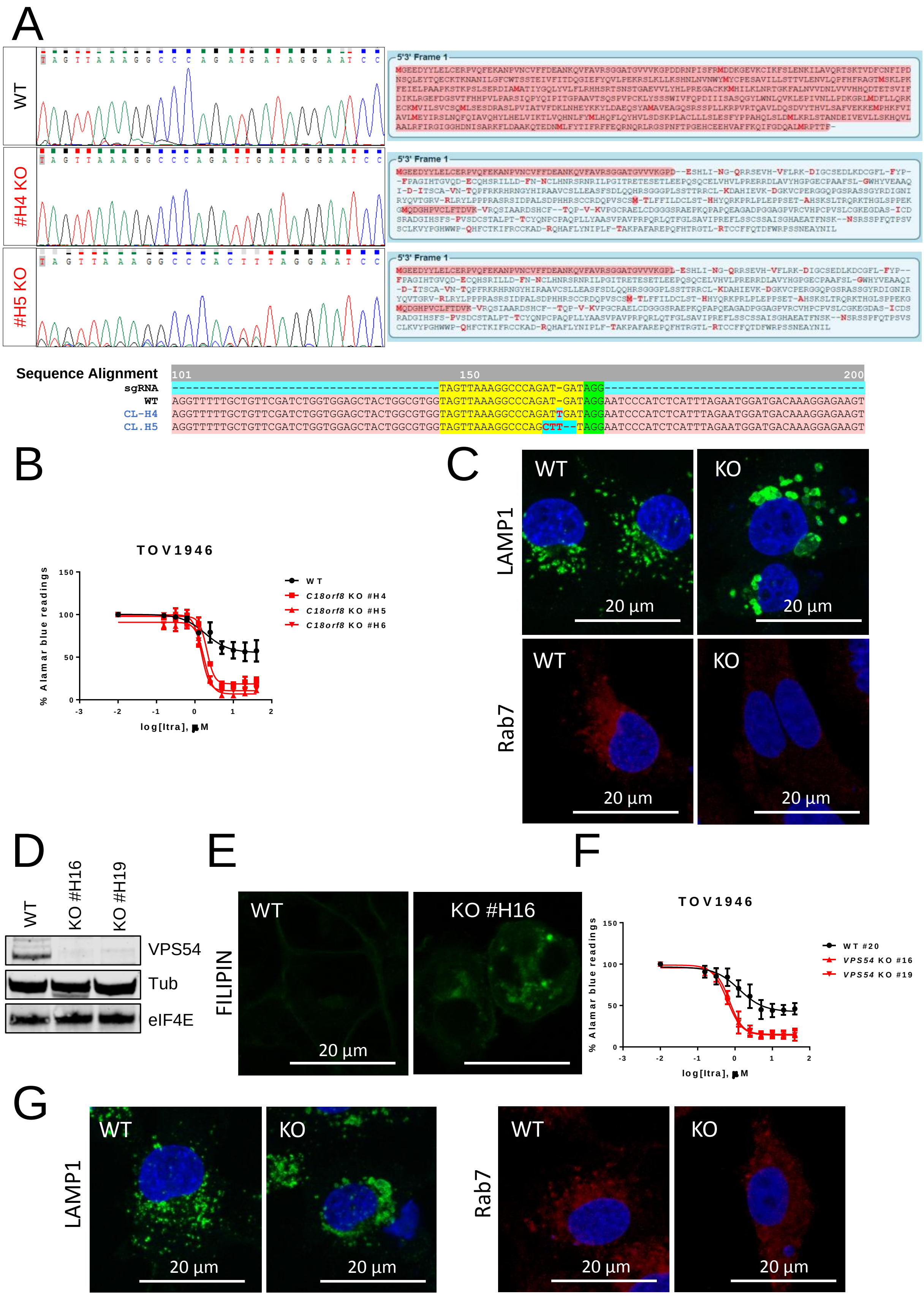
A
WT
#H4 KO
#H5 KO
5'3' Frame 1
Sequence Alignment
101
150
200
sgRNA ------------------------------------TAGTTAAAGGCCCAGAT-GATAGG------------------------------------
WT AGGTTTTTGCTGTTCGATCTGGTGGAGCTACTGGCGTGGTAGTTAAAGGCCCAGAT-GATAGGAATCCCATCTCATTTAGAATGGATGACAAAGGAGAAGT
CL-H4 AGGTTTTTGCTGTTCGATCTGGTGGAGCTACTGGCGTGGTAGTTAAAGGCCCAGATTGATAGGAATCCCATCTCATTTAGAATGGATGACAAAGGAGAAGT
CL.H5 AGGTTTTTGCTGTTCGATCTGGTGGAGCTACTGGCGTGGTAGTTAAAGGCCCAGCTT--TAGGAATCCCATCTCATTTAGAATGGATGACAAAGGAGAAGT
B
TOV1946
% Alamar blue readings
log[Itra], μM
WT
C18orf8 KO #H4
C18orf8 KO #H5
C18orf8 KO #H6
C
LAMP1
Rab7
WT
KO
20 μm
D
WT
KO #H16
KO #H19
VPS54
Tub
eIF4E
E
FILIPIN
WT
KO #H16
20 μm
F
TOV1946
% Alamar blue readings
log[Itra], μM
WT #20
VPS54 KO #16
VPS54 KO #19
G
LAMP1
Rab7
WT
KO
20 μm

Supplementary Figure 3. *C18orf8* and *VPS54* Knockout cells are more sensitive to Itra. **A,** Sequencing results (top-left) and alignment (bottom) of PCR amplicons reporting a homozygous CRISPR-induced indels and base editing in the exon2 of *C18orf8* gene upstream the AGG PAM sequence (reported in green). H4 and H5 represent two *C18orf8* knockout TOV1946 clones. Top right, protein translation of *C18orf8* in wild type versus H4 and H5 knockout clones. The knockout protein sequences (#H4 and H5) showed the insertion of a premature stop codon. **B,** Alamar blue results showing increased sensitivity to Itra in *C18orf8* knockout cells (red) compared to control (black) (N= 3 biological replicates). **C,** LAMP1 and Rab7 staining of wild type and *C18orf8* knockout cells (H5 was used as representative clone). **D,** Western blotting analysis showing knockout of *VPS54* in 2 independent TOV1946 clones. B-tubulin and eIF4E were used as a loading control. **E,** FILIPIN staining showing intracellular cholesterol accumulation in *VPS54* knockout cells compared to controls (H16 was used as representative clone). **F,** Alamar blue results showing increased sensitivity to Itra in *VPS54* knockout cells (red) compared to controls (black) (N= 3 biological replicates). **G,** LAMP1 and Rab7 staining of wild type and *C18orf8* knockout cells.

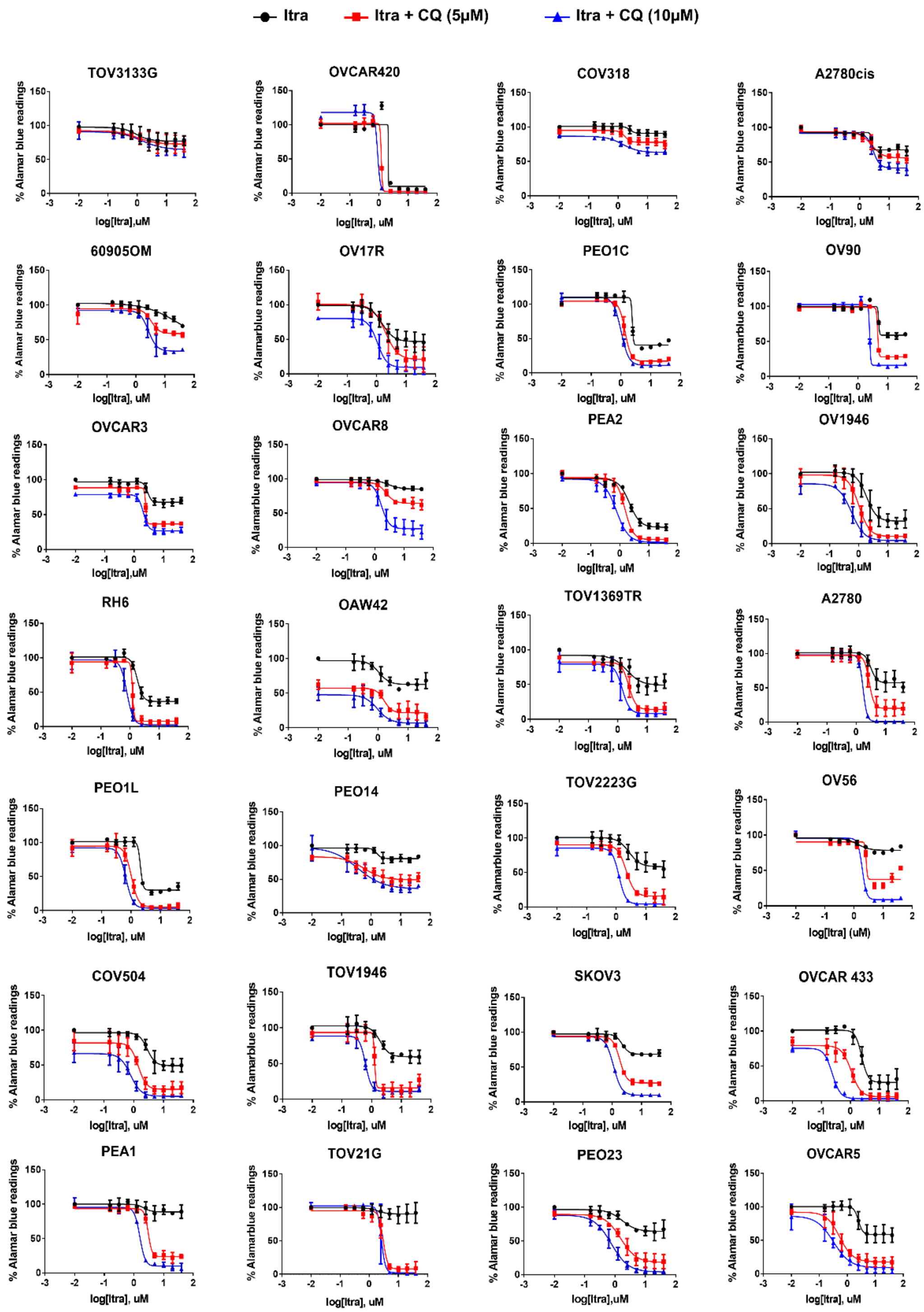

Supplementary Figure 4. Itra synergizes with chloroquine (CQ) in a panel of ovarian cancer cell lines. Alamar blue results showing the activity of Itra alone (black, 0-40μmol/L), or in combination with CQ 5 (red) and 10 (blue) μmol/L in a panel of 28 ovarian cancer cell lines. (N= 3 biological replicates).

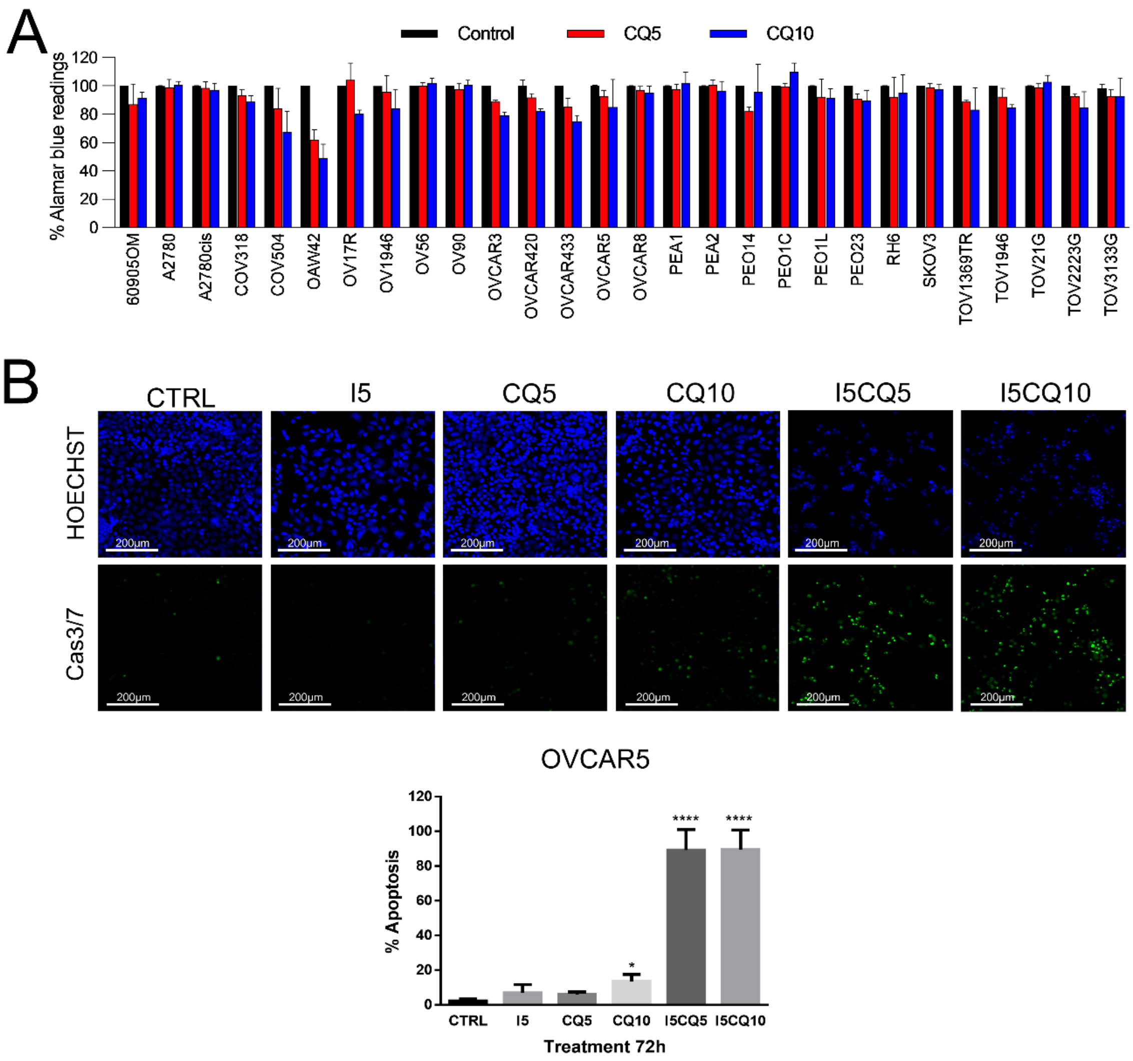

Supplementary Figure 5. Itra/CQ combination induce apoptosis in sensitive cells. **A,** Alamar blue results of cells treated with 5 (red) and 10 (blue) µmol/L CQ compared to control (black) (N= 3 biological replicates). **B,** Representative pictures (top) and quantification (bottom) of apoptotic rate in OVCAR5 cells treated with different combinations of Itra and CQ. *P < 0.05, **P < 0.01, ***P < 0.001, ****P < 0.0001; ns, not significant.

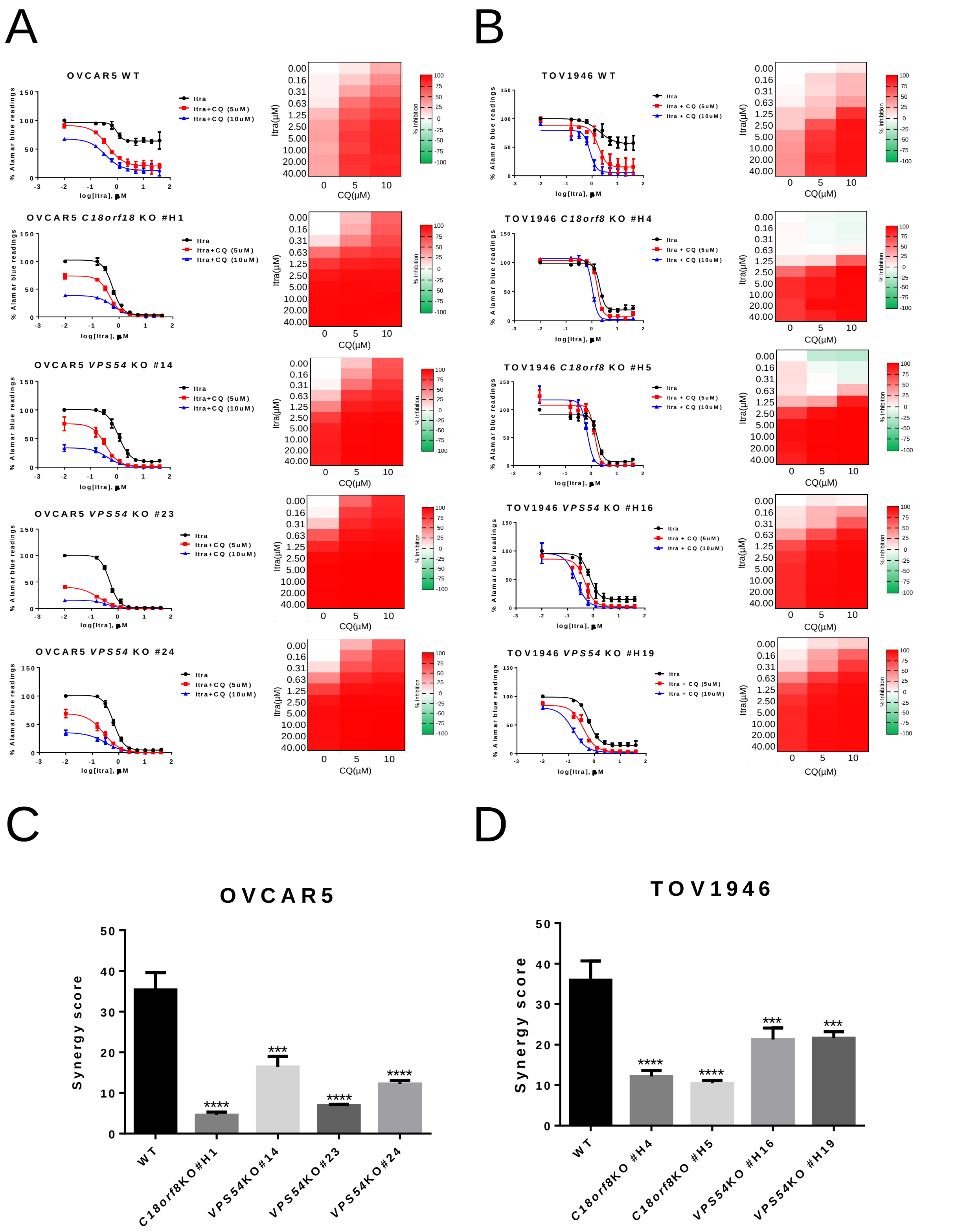

Supplementary Figure 6. Itra/CQ synergy scores are lower in *C18orf8/VPS54* knockout cells. **A-B,** Left, Alamar blue results of wild type and *C18orf8* and *VPS54* knockout cells (**A**, OVCAR5 and **B**, TOV1946) treated with Itra alone (black, 0-40 μmol/L) and Itra plus CQ 5 (red) and 10 (blue) μmol/L. Right, heat maps showing the inhibitory effect of the different drug combinations. **C-D,** Graph showing the Itra/CQ synergy score in wild type, *C18orf8* and *VPS54* knockout OVCAR5 (**C**) and TOV1946 cells (**D**). (N= 3 biological replicates). *P < 0.05, **P < 0.01, ***P < 0.001, ****P < 0.0001; ns, not significant.

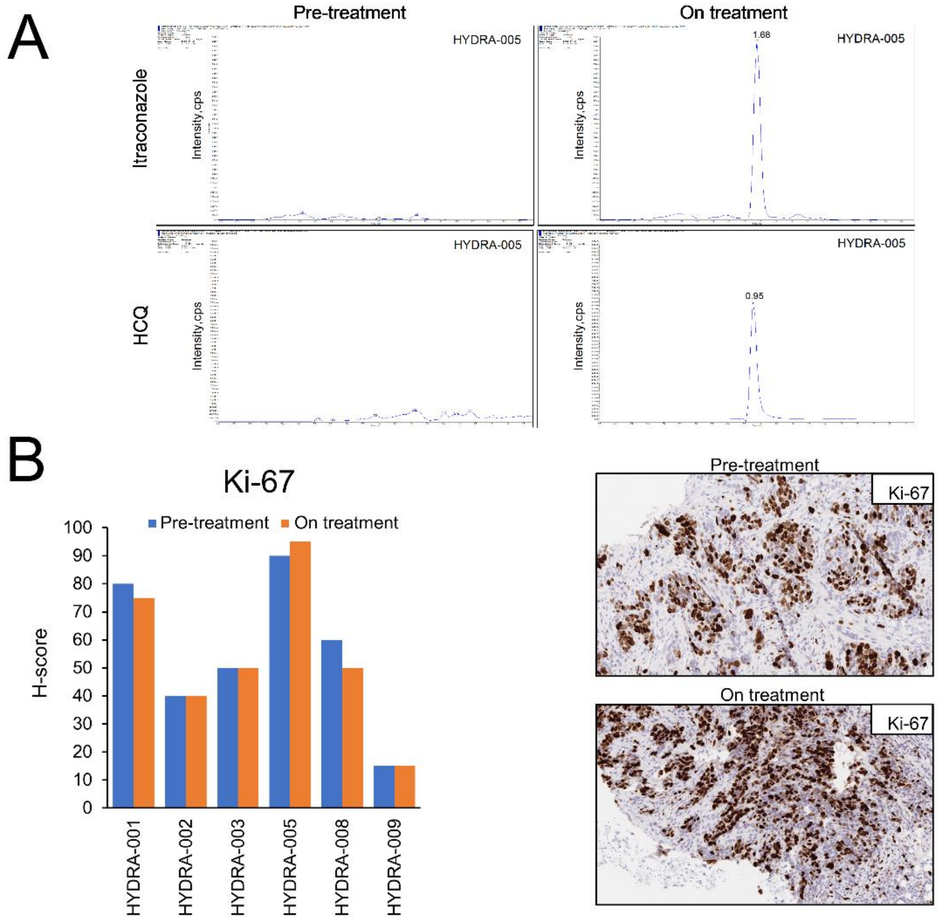

Supplementary Figure 7. Intratumoural Itra/hydroxychloroquine (HCQ) detection and Ki-67 analysis in patient samples. **A,** Chromatograms showing intra-tumuor detection of Itra (left) and HCQ (right) in HYDRA-005 patient pre- and post-treatment. Specific peaks showing retention for Itra (1.69 minutes) and HCQ (0.95 minutes) were observed in post-treatment samples. Measurements were done using HPLC-MS/MS method. **B,** Quantification of Ki-67 staining in HYDRA patients pre- and on treatment (graphs on the left) and representative picture (right) of patient HYDRA-005.
