# Supplementary material for: Repurposing Itraconazole and Hydroxychloroquine to Target Lysosomal Homeostasis in Epithelial Ovarian Cancer
Source: Cancer Res Commun. 2022 May 4;2(5):293–306. doi: 10.1158/2767-9764.CRC-22-0037 (PMC9981200; doi:10.1158/2767-9764.CRC-22-0037)
Supplement: Supplementary Tables 1-5 — Supplementary Tables 1 and 2 show respectively the fold change of lysosomal function and size in treated sensitive/resistant cells obtained by mixed effect modelling. Supplementary Table 3 shows patients enrolled per dose-level (DL) and dose limiting toxicities (DLTs). Supplementary Tables 4 and 5 show the summary of all and treatment related adverse effects. [file crc-22-0037-s04.pdf]

TABLE 1

Function

Comparison	RES	SENS	p
I5 vs. DMSO	1.05(1.04-1.07)	1.04(1.03-1.06)	0.3
CQ5 vs. DMSO	0.82(0.8-0.83)	0.69(0.68-0.69)	<0.0001
I5+CQ5 vs. DMSO	0.7(0.69-0.71)	0.29(0.29-0.3)	<0.0001

Supplementary Table 1. Fold change estimations of lysosomal function of Itra (I5), Chloroquine (CQ) and Itra/CQ combination (I5+CQ5) compared to control (DMSO) obtained by mixed effect modelling in Itra/CQ sensitive (SENS) and resistant (RES) cell lines.

TABLE 2

Diameter

Comparison	RES	SENS	p
I5 vs. DMSO	1.07(1.06-1.07)	1.16(1.15-1.17)	<0.0001
CQ5 vs.DMSO	1.32(1.31-1.33)	1.25(1.24-1.26)	<0.0001
I5+CQ5 vs. DMSO	1.36(1.34-1.37)	1.49(1.48-1.51)	<0.0001

Supplementary Table 2. Fold change estimations of lysosomal size (measured as diameter) of Itra (I5), Chloroquine (CQ) and Itra/CQ combination (I5+CQ5) compared to control (DMSO) obtained by mixed effect modelling in Itra/CQ sensitive (SENS) and resistant (RES) cell lines.

TABLE 3

	**N pts enrolled**	**N pts evaluable for efficacy**	**N of DLT**	**Type of DLT**
DL1<br>Itra 300mg BID<br>+<br>HCQ 200mg BID	5	5	0	-
DL2<br>Itra 300mg BID<br>+<br>HCQ 400mg BID	3	2	1	Grade 3 hypertension
DL3<br>Itra 300mg BID<br>+<br>HCQ 600mg BID	3	3	0	-

Supplementary Table 3. Patients enrolled per dose-level (DL) and dose limiting toxicities (DLTs).

TABLE 4

HYDRA_1:

Summary of all AEs:

AE term	Grade.1	Grade.2	Grade.3	Grade.4	Grade.5	Total
DIARRHEA	3 (27%)	3 (27%)	0 (0%)	0 (0%)	0 (0%)	6 (55%)
ABDOMINAL PAIN	0 (0%)	4 (36%)	1 (9%)	0 (0%)	0 (0%)	5 (45%)
FATIGUE	1 (9%)	3 (27%)	1 (9%)	0 (0%)	0 (0%)	5 (45%)
ASPARTATE AMINOTRANSFERASE INCREASED	4 (36%)	0 (0%)	0 (0%)	0 (0%)	0 (0%)	4 (36%)
BLOATING	2 (18%)	2 (18%)	0 (0%)	0 (0%)	0 (0%)	4 (36%)
DRY SKIN	3 (27%)	1 (9%)	0 (0%)	0 (0%)	0 (0%)	4 (36%)
NAUSEA	4 (36%)	0 (0%)	0 (0%)	0 (0%)	0 (0%)	4 (36%)
VOMITING	2 (18%)	1 (9%)	1 (9%)	0 (0%)	0 (0%)	4 (36%)
ALKALINE PHOSPHATASE INCREASED	1 (9%)	0 (0%)	2 (18%)	0 (0%)	0 (0%)	3 (27%)
ANEMIA	2 (18%)	1 (9%)	0 (0%)	0 (0%)	0 (0%)	3 (27%)
CONSTIPATION	1 (9%)	2 (18%)	0 (0%)	0 (0%)	0 (0%)	3 (27%)
EARLY SATIETY	3 (27%)	0 (0%)	0 (0%)	0 (0%)	0 (0%)	3 (27%)
PRURITUS	3 (27%)	0 (0%)	0 (0%)	0 (0%)	0 (0%)	3 (27%)
RIGHT LOWER QUADRANT PAIN	1 (9%)	1 (9%)	1 (9%)	0 (0%)	0 (0%)	3 (27%)
ALANINE AMINOTRANSFERASE INCREASED	2 (18%)	0 (0%)	0 (0%)	0 (0%)	0 (0%)	2 (18%)
ANOREXIA	1 (9%)	1 (9%)	0 (0%)	0 (0%)	0 (0%)	2 (18%)
BACK PAIN	1 (9%)	1 (9%)	0 (0%)	0 (0%)	0 (0%)	2 (18%)
BRUISING	2 (18%)	0 (0%)	0 (0%)	0 (0%)	0 (0%)	2 (18%)
COUGH	1 (9%)	1 (9%)	0 (0%)	0 (0%)	0 (0%)	2 (18%)

CREATININE INCREASED	2 (18%)	0 (0%)	0 (0%)	0 (0%)	0 (0%)	2 (18%)
DEHYDRATION	0 (0%)	1 (9%)	1 (9%)	0 (0%)	0 (0%)	2 (18%)
DIZZINESS	2 (18%)	0 (0%)	0 (0%)	0 (0%)	0 (0%)	2 (18%)
DYSPEPSIA	0 (0%)	2 (18%)	0 (0%)	0 (0%)	0 (0%)	2 (18%)
ELECTROCARDIOGRAM QT CORRECTED INTERVAL PROLONGED	1 (9%)	0 (0%)	0 (0%)	1 (9%)	0 (0%)	2 (18%)
HYPERTENSION	0 (0%)	0 (0%)	2 (18%)	0 (0%)	0 (0%)	2 (18%)
HYPONATREMIA	1 (9%)	0 (0%)	1 (9%)	0 (0%)	0 (0%)	2 (18%)
MUSCLE WEAKNESS LOWER LIMB	0 (0%)	2 (18%)	0 (0%)	0 (0%)	0 (0%)	2 (18%)
NEUTROPHIL COUNT DECREASED	1 (9%)	1 (9%)	0 (0%)	0 (0%)	0 (0%)	2 (18%)
WHITE BLOOD CELL DECREASED	0 (0%)	2 (18%)	0 (0%)	0 (0%)	0 (0%)	2 (18%)
"LUMP" LOWER RIGHT QUADRANT	1 (9%)	0 (0%)	0 (0%)	0 (0%)	0 (0%)	1 (9%)
ACID REFLUX	0 (0%)	1 (9%)	0 (0%)	0 (0%)	0 (0%)	1 (9%)
ARTHRALGIA	0 (0%)	1 (9%)	0 (0%)	0 (0%)	0 (0%)	1 (9%)
BOWEL OBSTRUCTION	0 (0%)	0 (0%)	1 (9%)	0 (0%)	0 (0%)	1 (9%)
CACHECTIC	0 (0%)	1 (9%)	0 (0%)	0 (0%)	0 (0%)	1 (9%)
CHANGE IN CALIBRE OF STOOL - THIN STOOL	1 (9%)	0 (0%)	0 (0%)	0 (0%)	0 (0%)	1 (9%)
CHILLS	1 (9%)	0 (0%)	0 (0%)	0 (0%)	0 (0%)	1 (9%)
DRY EYE	1 (9%)	0 (0%)	0 (0%)	0 (0%)	0 (0%)	1 (9%)
DYSGEUSIA	1 (9%)	0 (0%)	0 (0%)	0 (0%)	0 (0%)	1 (9%)
DYSPNEA	0 (0%)	0 (0%)	1 (9%)	0 (0%)	0 (0%)	1 (9%)
EDEMA AT BIOPSY SITE	1 (9%)	0 (0%)	0 (0%)	0 (0%)	0 (0%)	1 (9%)
EDEMA LIMBS	1 (9%)	0 (0%)	0 (0%)	0 (0%)	0 (0%)	1 (9%)
ESOPHAGITIS	0 (0%)	0 (0%)	1 (9%)	0 (0%)	0 (0%)	1 (9%)
FLATULENCE	1 (9%)	0 (0%)	0 (0%)	0 (0%)	0 (0%)	1 (9%)
GAIT DISTURBANCE	1 (9%)	0 (0%)	0 (0%)	0 (0%)	0 (0%)	1 (9%)
GASTRIC ULCER	0 (0%)	0 (0%)	1 (9%)	0 (0%)	0 (0%)	1 (9%)
GENERALIZED MUSCLE WEAKNESS	0 (0%)	1 (9%)	0 (0%)	0 (0%)	0 (0%)	1 (9%)
HEADACHE	1 (9%)	0 (0%)	0 (0%)	0 (0%)	0 (0%)	1 (9%)

HEMATOMA	1 (9%)	0 (0%)	0 (0%)	0 (0%)	0 (0%)	1 (9%)
HEMATURIA	1 (9%)	0 (0%)	0 (0%)	0 (0%)	0 (0%)	1 (9%)
HOARSENESS	1 (9%)	0 (0%)	0 (0%)	0 (0%)	0 (0%)	1 (9%)
HYPERCALCEMIA	1 (9%)	0 (0%)	0 (0%)	0 (0%)	0 (0%)	1 (9%)
HYPOCALCEMIA	1 (9%)	0 (0%)	0 (0%)	0 (0%)	0 (0%)	1 (9%)
HYPOGLYCEMIA	1 (9%)	0 (0%)	0 (0%)	0 (0%)	0 (0%)	1 (9%)
HYPOKALEMIA	0 (0%)	0 (0%)	1 (9%)	0 (0%)	0 (0%)	1 (9%)
INCREASED SENSE OF SMELL	1 (9%)	0 (0%)	0 (0%)	0 (0%)	0 (0%)	1 (9%)
INSOMNIA	1 (9%)	0 (0%)	0 (0%)	0 (0%)	0 (0%)	1 (9%)
LEFT LOWER QUADRANT PAIN	1 (9%)	0 (0%)	0 (0%)	0 (0%)	0 (0%)	1 (9%)
LUMP ON RIGHT SIDE OF CHEST	1 (9%)	0 (0%)	0 (0%)	0 (0%)	0 (0%)	1 (9%)
LUMP UNDER SKIN LOWER RIGHT QUADRANT	1 (9%)	0 (0%)	0 (0%)	0 (0%)	0 (0%)	1 (9%)
OXYGEN DESATURATION	0 (0%)	1 (9%)	0 (0%)	0 (0%)	0 (0%)	1 (9%)
PARTIAL BOWEL OBSTRUCTION	0 (0%)	0 (0%)	1 (9%)	0 (0%)	0 (0%)	1 (9%)
PERIPHERAL SENSORY NEUROPATHY	0 (0%)	1 (9%)	0 (0%)	0 (0%)	0 (0%)	1 (9%)
PNEUMONIA (SUSPECTED)	0 (0%)	1 (9%)	0 (0%)	0 (0%)	0 (0%)	1 (9%)
PROTEINURIA	1 (9%)	0 (0%)	0 (0%)	0 (0%)	0 (0%)	1 (9%)
RECTAL HEMORRHAGE	1 (9%)	0 (0%)	0 (0%)	0 (0%)	0 (0%)	1 (9%)
RIGHT GROIN PAIN	0 (0%)	0 (0%)	1 (9%)	0 (0%)	0 (0%)	1 (9%)
SUN SENSITIVITY	1 (9%)	0 (0%)	0 (0%)	0 (0%)	0 (0%)	1 (9%)
TACHYCARDIA	1 (9%)	0 (0%)	0 (0%)	0 (0%)	0 (0%)	1 (9%)
TOOTHACHE	1 (9%)	0 (0%)	0 (0%)	0 (0%)	0 (0%)	1 (9%)
UNSTEADY GAIT	0 (0%)	1 (9%)	0 (0%)	0 (0%)	0 (0%)	1 (9%)
UPPER RESPIRATORY INFECTION	0 (0%)	1 (9%)	0 (0%)	0 (0%)	0 (0%)	1 (9%)
URINARY FREQUENCY	1 (9%)	0 (0%)	0 (0%)	0 (0%)	0 (0%)	1 (9%)
WEIGHT LOSS	0 (0%)	1 (9%)	0 (0%)	0 (0%)	0 (0%)	1 (9%)
YELLOW SKIN ON PALMS OF HANDS	1 (9%)	0 (0%)	0 (0%)	0 (0%)	0 (0%)	1 (9%)

TABLE 5

Summary of treatment related AEs:

AE term	Grade.1	Grade.2	Grade.3	Grade.4	Grade.5	Total
NAUSEA	4 (36%)	0 (0%)	0 (0%)	0 (0%)	0 (0%)	4 (36%)
DIARRHEA	1 (9%)	2 (18%)	0 (0%)	0 (0%)	0 (0%)	3 (27%)
DRY SKIN	3 (27%)	0 (0%)	0 (0%)	0 (0%)	0 (0%)	3 (27%)
FATIGUE	1 (9%)	2 (18%)	0 (0%)	0 (0%)	0 (0%)	3 (27%)
VOMITING	2 (18%)	1 (9%)	0 (0%)	0 (0%)	0 (0%)	3 (27%)
ALANINE AMINOTRANSFERASE INCREASED	2 (18%)	0 (0%)	0 (0%)	0 (0%)	0 (0%)	2 (18%)
ANEMIA	1 (9%)	1 (9%)	0 (0%)	0 (0%)	0 (0%)	2 (18%)
ANOREXIA	2 (18%)	0 (0%)	0 (0%)	0 (0%)	0 (0%)	2 (18%)
ASPARTATE AMINOTRANSFERASE INCREASED	2 (18%)	0 (0%)	0 (0%)	0 (0%)	0 (0%)	2 (18%)
CONSTIPATION	1 (9%)	1 (9%)	0 (0%)	0 (0%)	0 (0%)	2 (18%)
ELECTROCARDIOGRAM QT CORRECTED INTERVAL PROLONGED	1 (9%)	0 (0%)	0 (0%)	1 (9%)	0 (0%)	2 (18%)
NEUTROPHIL COUNT DECREASED	1 (9%)	1 (9%)	0 (0%)	0 (0%)	0 (0%)	2 (18%)
PRURITUS	2 (18%)	0 (0%)	0 (0%)	0 (0%)	0 (0%)	2 (18%)
WHITE BLOOD CELL DECREASED	0 (0%)	2 (18%)	0 (0%)	0 (0%)	0 (0%)	2 (18%)
ARTHRALGIA	0 (0%)	1 (9%)	0 (0%)	0 (0%)	0 (0%)	1 (9%)
CREATININE INCREASED	1 (9%)	0 (0%)	0 (0%)	0 (0%)	0 (0%)	1 (9%)
DEHYDRATION	1 (9%)	0 (0%)	0 (0%)	0 (0%)	0 (0%)	1 (9%)
DIZZINESS	1 (9%)	0 (0%)	0 (0%)	0 (0%)	0 (0%)	1 (9%)
DRY EYE	1 (9%)	0 (0%)	0 (0%)	0 (0%)	0 (0%)	1 (9%)
DYSGEUSIA	1 (9%)	0 (0%)	0 (0%)	0 (0%)	0 (0%)	1 (9%)
GAIT DISTURBANCE	1 (9%)	0 (0%)	0 (0%)	0 (0%)	0 (0%)	1 (9%)
GENERALIZED MUSCLE WEAKNESS	0 (0%)	1 (9%)	0 (0%)	0 (0%)	0 (0%)	1 (9%)
HEMATOMA	1 (9%)	0 (0%)	0 (0%)	0 (0%)	0 (0%)	1 (9%)

HYPERTENSION	0 (0%)	0 (0%)	1 (9%)	0 (0%)	0 (0%)	1 (9%)
HYPOCALCEMIA	1 (9%)	0 (0%)	0 (0%)	0 (0%)	0 (0%)	1 (9%)
HYPOKALEMIA	0 (0%)	0 (0%)	1 (9%)	0 (0%)	0 (0%)	1 (9%)
HYPONATREMIA	1 (9%)	0 (0%)	0 (0%)	0 (0%)	0 (0%)	1 (9%)
INCREASED SENSE OF SMELL	1 (9%)	0 (0%)	0 (0%)	0 (0%)	0 (0%)	1 (9%)
MUSCLE WEAKNESS LOWER LIMB	0 (0%)	1 (9%)	0 (0%)	0 (0%)	0 (0%)	1 (9%)
PERIPHERAL SENSORY NEUROPATHY	0 (0%)	1 (9%)	0 (0%)	0 (0%)	0 (0%)	1 (9%)
PROTEINURIA	1 (9%)	0 (0%)	0 (0%)	0 (0%)	0 (0%)	1 (9%)
UNSTEADY GAIT	0 (0%)	1 (9%)	0 (0%)	0 (0%)	0 (0%)	1 (9%)
YELLOW SKIN ON PALMS OF HANDS	1 (9%)	0 (0%)	0 (0%)	0 (0%)	0 (0%)	1 (9%)

Supplementary Table 4. Summary of all adverse events. ALT: Alanine aminotransferase, AST: Aspartate aminotransferase

Supplementary Table 5. Summary of the treatment related adverse events.
